# Synthesis of Diarylamines via Nitrosonium-Initiated C–N Bond Formation

**DOI:** 10.1021/acs.joc.4c01220

**Published:** 2024-07-01

**Authors:** Pin-Hsien Chen, Shu-Jung Hsu, Cheng-Chun Chen, Jui-Chen Fu, Duen-Ren Hou

**Affiliations:** Department of Chemistry, National Central University, 300 Jhong-Da Rd., Jhong-Li, Taoyuan 320317, Taiwan

## Abstract

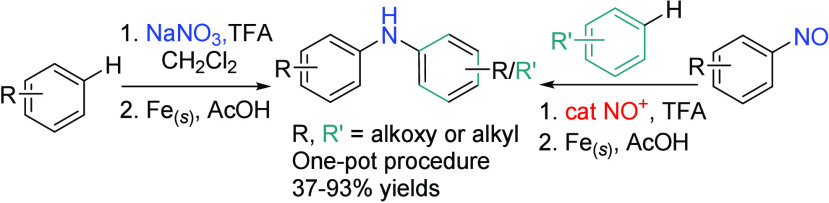

Electron-rich diarylamines, exemplified by anisole-derived amines, play pivotal roles in process chemistry, pharmaceuticals, and materials. In this study, homo-diarylamines were synthesized directly from the C–H activation of electron-rich arenes by sodium nitrate/trifluoroacetic acid and the successive treatment of iron powder. Mechanistic investigations reveal that nitrosoarene serves as the reaction intermediate, and the formation of the second C–N bond between the resulting nitrosoarene and electron-rich arene is catalyzed by the nitrosonium ion (NO^+^). Thus, hetero-diarylamines were synthesized using preformed nitrosoarenes and various electron-rich arenes. This reaction complements a range of cross-coupling reactions catalyzed by transition metal catalysts.

## Introduction

**Figure 1:**
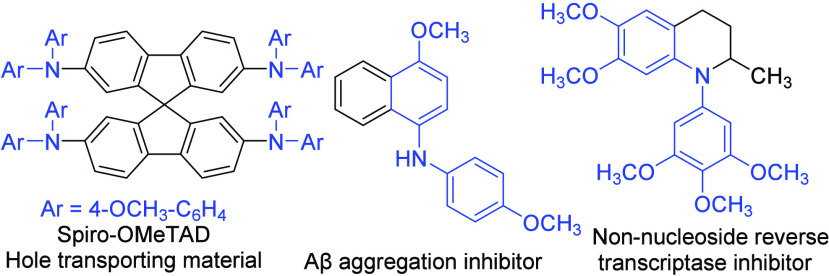
Representative anisole-derived diarylamines.

**Scheme 1:**
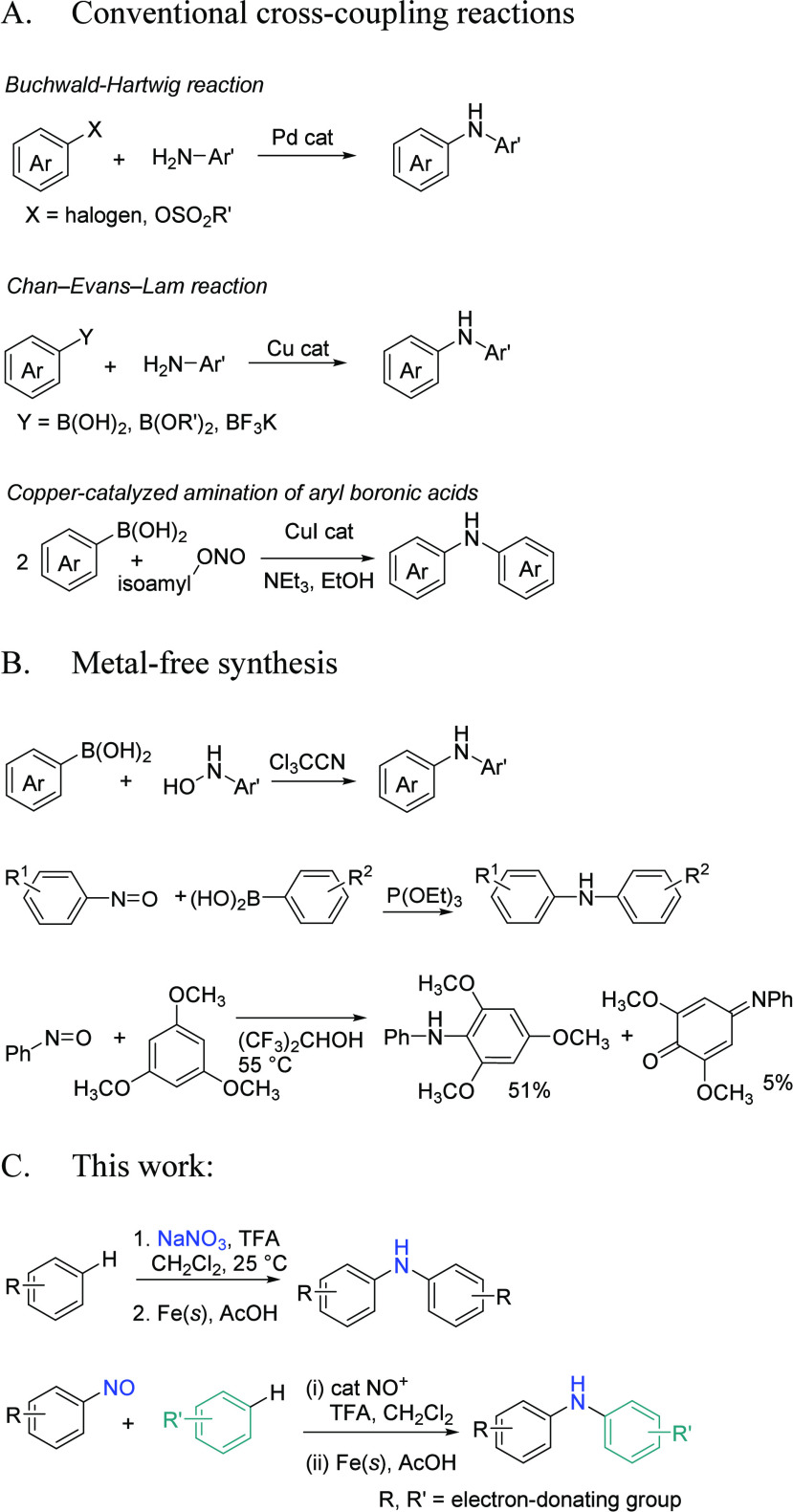
Methods for the Synthesis of Diarylamines

Diarylamines, particularly those based on the structure of anisole, serve as essential building blocks in the synthesis of photomaterials and electronic materials.^1^ Their electron-donating and hole-transporting properties are crucial for creating advanced materials used in optoelectronics and photovoltaics.^2^ The structural motif originating from anisole-derived diarylamines is also prevalent in many biologically active compounds. (Figure 1).^3^ Consequently, there is a strong demand for an efficient and environmentally friendly protocol for the synthesis of these diarylamines.^4^ Conventional cross-coupling reactions, including the palladium-catalyzed Buchwald–Hartwig reaction,^5^ copper-catalyzed Chan–Evans–Lam reaction,^6^ Ullmann-type reaction,^7^ and various cross coupling reactions^8^ facilitated by transition metal catalysts between arylamines and aryl halides/boronic acids,^9,10^ are commonly employed for the synthesis of diarylamines (Scheme 1A). Recently, the merger of photoredox catalysis and transition metal catalysis has also been utilized to form C(sp^2^)–N bonds.^11^ However, concerns arise from the use of costly and environmentally harmful transition metal catalysts in these coupling processes, along with the presence of their residual contaminants in the final products.^12^ Alternative methods to synthesize diaryl amines include nucleophilic aromatic substitution (S_N_Ar) reactions,^13^ the insertion of benzyne into amide,^14^ coupling reactions between aryl boronic acid and hydroxylamines,^15^ desulfinylative Smiles rearrangement,^16^ hypervalent-iodine-mediated cross-amination,^17^ and the reactions of nitrosoarenes with aryl boronic acids or 1,3,5-trimethoxybenzene (Scheme 1B).^18,19^ In this work, we found that both homo- and hetero-diarylamines can be prepared from electron-rich arenes by nitrosonium (NO^+^) ion-initiated C–H activation and C–N bond formation (Scheme 1C).

## Results and Discussion

**Figure 2:**
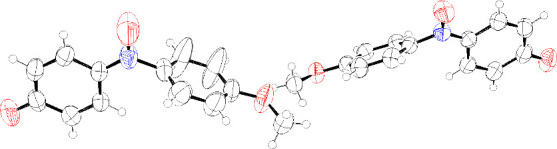
Oak Ridge thermal ellipsoid plot of **2** with ellipsoids set to 50% probability.

While studying the reaction of sodium nitrate and anisole (**1a**) with trifluoroacetic acid (TFA, eq 1),^20^ we accidentally isolated a red compound, whose structure was later identified as *N*-(4-methoxyphenyl)-4-oxocyclohexa-2,5-dien-1-imine oxide (**2**) by NMR spectroscopy^21^ and X-ray crystallography (CCDC 2322222, Figure 2).
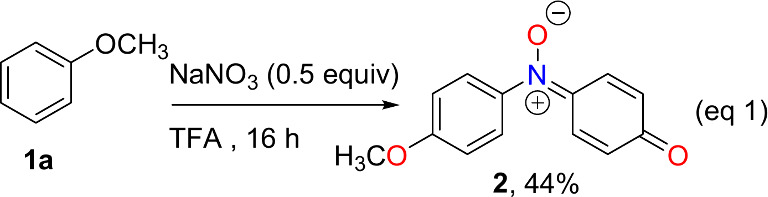
1

We suspected that product **2** formed from the demethylation of aminium *N*-oxide **2′** during the isolation step. Therefore, before proceeding with the workup, the reaction mixture underwent an additional treatment with iron powder (eq 2). Indeed, diarylamine **3a**, resulting from the reduction of aminium *N*-oxide **2′**, was harvested in a 54% yield.
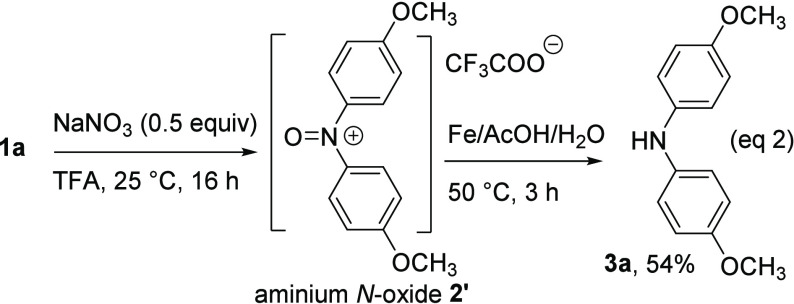
2

The reaction conditions were further optimized with *n*-hexyl phenyl ether (**1b**, Table 1). The elevated boiling point^22^ of **1b** is advantageous for facile tracking of **1b** and the products diarylamine **3b** and primary amine **4b**, both of which were generated in this reaction. Following the original conditions of eq 2, only a moderate yield (38%) of **3b** was obtained (entry 1). After futile attempts to modify the reaction time and the equivalents of reagents to improve the yields (see Table S1 in the Supporting Information), we conducted the reaction in a range of solvents (entries 2–9) and found that 1,2-dichloroethane and dichloromethane significantly enhanced the yield of **3b** to 68% and 70%, respectively (entries 2 and 3). When the reaction scale was increased to 10 mmol **1b**, the yield of **3b** was maintained (entry 4). While toluene and acetic acid-derived solvents yielded only modest results (entries 5–9), the reactions conducted in methanol, diethyl ether, THF, and DMF failed to produce any diarylamine (Table S1).

**Table 1:**
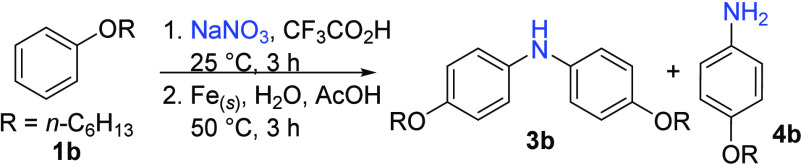
Optimization of Diarylamine Formation^a^

entry	solvent and reaction time for the first step	yield of **3b** (%)	yield of **4b** (%)
1	trifluoroacetic acid^b^	38	9
2	1,2-dichloroethane	68	10
3	dichloromethane	70	8
4^c^	dichloromethane	71	15
5	toluene, 16 h	38	6
6	acetic acid	40	12
7	acetonitrile, 5 h	44	22
8	acetone, 16 h	32	4
9	ethyl acetate, 16 h	8	1

a Sodium nitrate (1.0 mmol) was added to a solution of **1b** (178.3 mg, 1.0 mmol), TFA (0.5 mL, 0.74 g, 6.5 mmol), and a solvent (1.0 mL) at 0 °C. After stirring at rt for 3 h under an atmosphere of air (balloon), to the reaction mixture were added iron powder (0.56 g, 10 mmol), acetic acid (1 mL), and water (0.5 mL), and the resultant mixture was stirred at 50 °C for another 3 h. Yields were determined by ^1^H NMR using 1,4-dimethoxybenzene as an internal standard.

b TFA (total 1.5 mL), no other solvent.

c **1b** (1.78 g, 10.0 mmol) was applied; **3b** (1.32 g, 3.57 mmol, 71%) and **4b** (0.29 g, 1.53 mmol, 15%) were isolated.

The optimized reaction condition (25 °C, 3 h, and the treatment of iron at 50 °C for 3 h) was applied to prepare more diarylamines, and the reaction scope was examined (Table 2). In dichloromethane, the yield of **3a** was improved to 66% (entry 1). Compound **3a**, pivotal as a precursor to a range of estrogen receptor (ER) antagonists^3c^ and Spiro-OMeTAD,^23^ a crucial hole-transporting material, was traditionally synthesized via a Buchwald–Hartwig coupling reaction conducted at high temperatures (>100 °C).^3c,24^ All 2-alkyl substituted anisoles (**1c**–**1g**, entries 2–6) produced the corresponding diarylamines **3c**–**3g** in 56–68% yields. The structure of **3d** was also confirmed by X-ray crystallography (CCDC 2322240, see the Supporting Information). As a result, the reactions were slightly affected by the steric hindrance of these *ortho*-substituents. On the other hand, employing 2,6-dimethylanisole (**1h**) necessitated an extended reaction time (16 h) and resulted in a lower yield (32%, entry 7). Both 3-methyl- and 3-methoxy-substituted anisoles, **1i** and **1j**, respectively, were converted to **3i** and **3j** smoothly (entries 8 and 9). The substituents on the oxygen atom, such as phenyl and *n*-hexyl groups, did not affect this reaction, yielding diarylamines **3k**–**3m**, respectively (entries 10–12). 4-Methylanisole and toluene did not produce any diarylamines under the reaction condition.

**Table 2:**
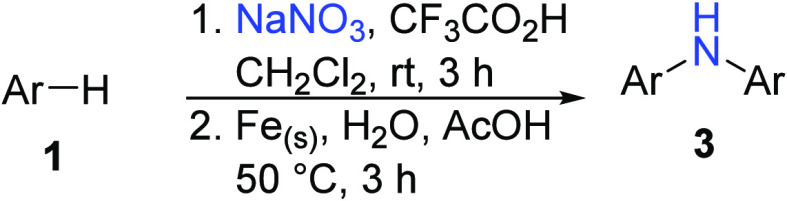

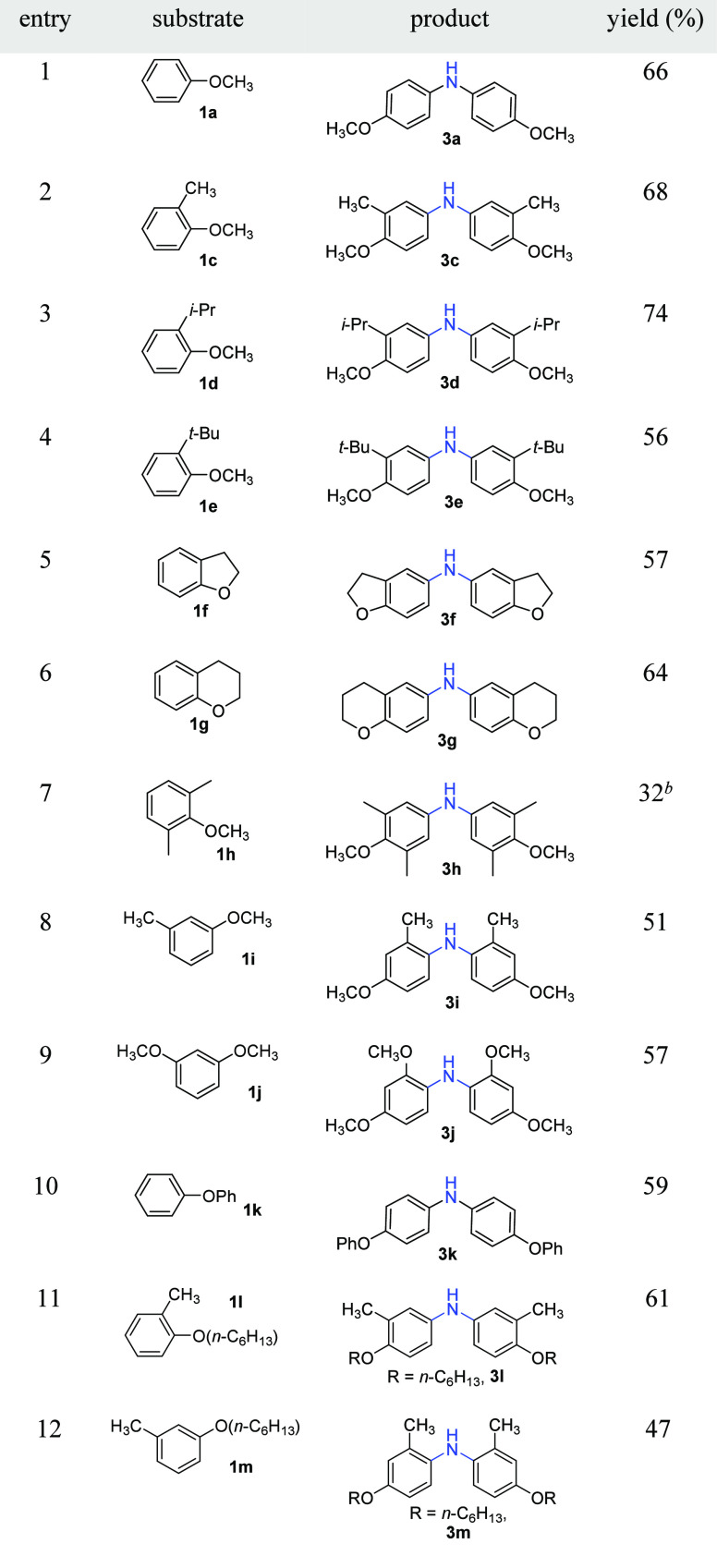
Study on the Substrate Scope^a^

a Sodium nitrate (1.0 mmol) was added to a solution of **1b** (178.3 mg, 1.0 mmol), TFA (0.5 mL, 0.74 g, 6.5 mmol), and CH_2_Cl_2_ (1.0 mL) at 0 °C. After stirring at rt for 3 h under an atmosphere of air (balloon), to the reaction mixture were added iron powder (0.56 g, 10 mmol), acetic acid (1 mL), and water (0.5 mL), and the resultant mixture was stirred at 50 °C for another 3 h.

b The reaction time for the first step was 16 h.

Due to the consistent presence of primary amine **4b** as a byproduct in Table 1, we initially suspected that 4-nitroanisole was the reaction intermediate, which further reacted with another anisole to yield secondary amine **3**. However, treating the mixture of 4-nitroanisole (**5a**) and anisole (**1a**) with TFA, a trace of NaNO_3_, and then iron powder yielded only primary amine **4a**, with 91% of anisole recovered (eq 3), indicating that nitro-**5a** is unlikely to be the reaction intermediate. On the other hand, subjecting separately prepared 1-methoxy-4-nitrosobenzene (**6a**) to the mixture of **1a**, TFA, and nitrosonium tetrafluoroborate (NOBF_4_), a source of nitrosonium (NO^+^) cation, yielded imine oxide **2** (87%, eq 4), the same product observed in eq 1 derived from the demethylation of **2′** in the absence of iron. It is known that the reaction between NO^+^ and anisole **1a** efficiently produces nitroso-**6a**.^25^ Additionally, under acidic conditions, sodium nitrate can generate NO^+^ similar to NOBF_4_.^26^ It is reasonable to propose that nitrosoarene **6a** serves as the reaction intermediate and its further reaction with another molecule of anisole to form **2** (through aminium *N*-oxide **2′** and subsequent demethylation). This speculation was further supported with this experiment: when **1a** was replaced with **1c** and the reducing workup procedure (iron powder) was applied, a new hetero-diarylamine (**3ac**) was harvested in 83% yield (eq 5). Clearly, product **3ac** was derived from the reaction between nitroso-**6a** and anisole **1c**. In contrast, the corresponding reaction between **1c** and nitro-**5a** only gave primary amine **4a** and homo-diarylamine **3c**, derived from the reduction of inactive nitro-**5a** and the sequential nitrosation and coupling reaction of **1c**, respectively (eq 6). In addition to NaNO_3_, both NOBF_4_ and *tert*-butyl nitrite, also precursors to generate NO^+^,^27^ could be the source of nitrogen atoms to produce diarylamine **3b** with similar yields (eq 7). In these reactions, the formation of primary amine **4b** was negligible (<3%). As little as 0.1 equiv of sodium azide, a NO^+^ scavenger,^28^ was able to inhibit this reaction completely (eq 48), consistent with a nitrosonium-catalyzed reaction.

The reaction conditions to generate hetero-diarylamine **3ac** using nitrosoarene **6a** and anisole **1c** were further optimized (Table 3). Without the addition of NOBF_4_, the formation of **3ac** was limited (18%, entry 1). The addition of catalytic amounts of NOBF_4_ (10–50 mol %) significantly increased the yields of **3ac** (entries 2–4), but the difference between 30 or 50 mol % of NOBF_4_ applied was very minor (entry 3 versus entry 4). The need for oxygen was shown in the yields of **3ac** derived from reactions carried out in atmospheres of oxygen, air, and nitrogen (entry 3 versus entries 5 and 6). The yields were also proportional to the amount of TFA applied (entries 7–9), indicating the requirement of strongly acidic conditions. The detailed study on the preparation of diarylamine **3b** with various NO^+^ precursors and the positive effect of oxygen are summarized in Table S2 (Supporting Information).
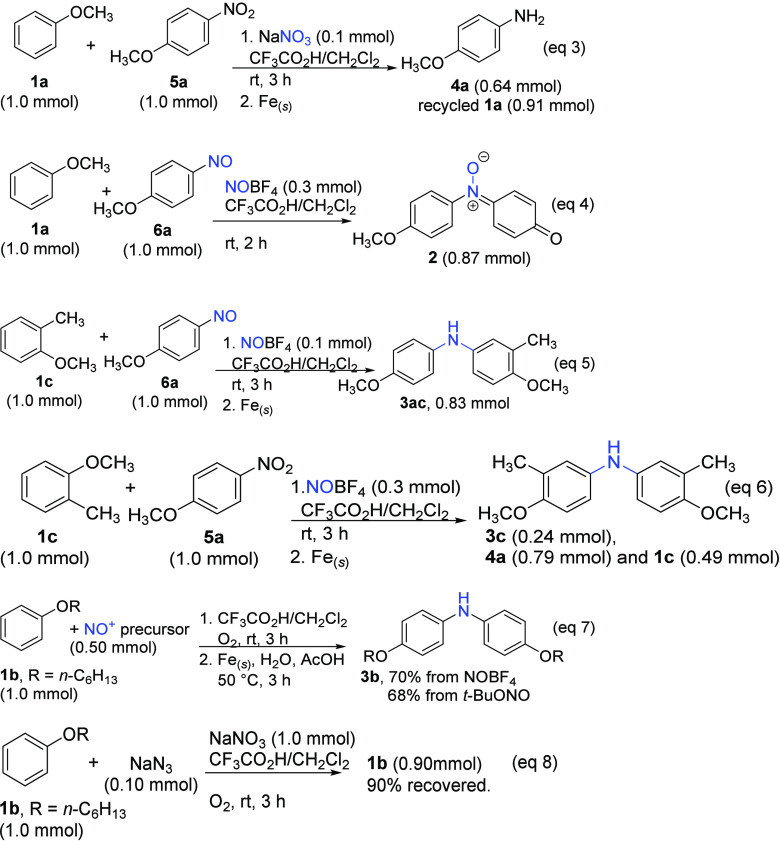
3

**Table 3:**
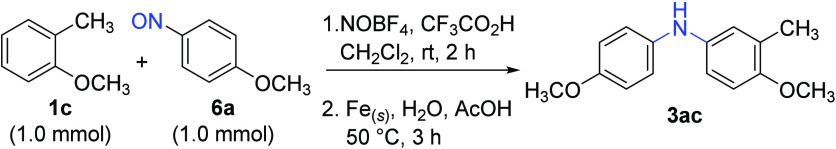
Formation of Hetero-Diarylamine **3ac**^a^

entry	NOBF_4_ (equiv)	TFA (equiv)	atmosphere	**3ac** (%)
1	0	6.5	O_2_	18
2	0.1	6.5	O_2_	83
3	0.3	6.5	O_2_	92
4	0.5	6.5	O_2_	93
5	0.3	6.5	air	81
6	0.3	6.5	N_2_	40
7	0.3	4.4	O_2_	58
8	0.3	2.2	O_2_	33
9	0.3	0	O_2_	17

a **1c** (1.0 mmol) was added to a solution of **6a** (1.0 mmol), NOBF_4_, TFA, and CH_2_Cl_2_ (1.0 mL) at 0 °C. After stirring at rt for 2 h, iron powder (0.56 g, 10 mmol), acetic acid (1 mL), and water (0.5 mL) were added to the reaction mixture, and the resultant mixture was stirred at 50 °C for another 3 h. Yields were determined by ^1^H NMR using 1,4-dimethoxybenzene as an internal standard.

More hetero-diarylamines were synthesized via the NO^+^-catalyzed C–N bond formation between nitrosoarenes and arenes (Table 4). For example, the reactions between nitroso-**6a** and various anisole derivatives gave **3ab**, **3ad–-3af**, **3ah**, **3ai**, and **3ak** in good yields. Although diarylamine cannot be prepared from toluene directly, hetero-diarylamines **3ba**, **3bc**, **3br**, and **3bs**, all containing a *para*-tolyl group, were synthesized using 4-nitrosotoluene (**6b**) and the corresponding anisole derivatives. Chlorinated and brominated anisoles **1r** and **1s**, respectively, were compatible substrates to give halogenated diarylamines **3br** and **3bs**. We were pleased to discover that the reactions of *m*-xylene (**1n**) with nitroso-**6a** and nitroso-**6b** were achieved with 5 equiv of **1n** and portion-wise additions of NOBF_4_ to give the respective diarylamines **3an** and **3bn**.

**Table 4:**
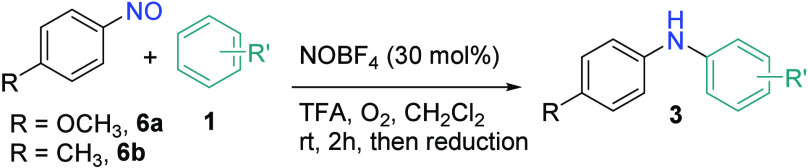

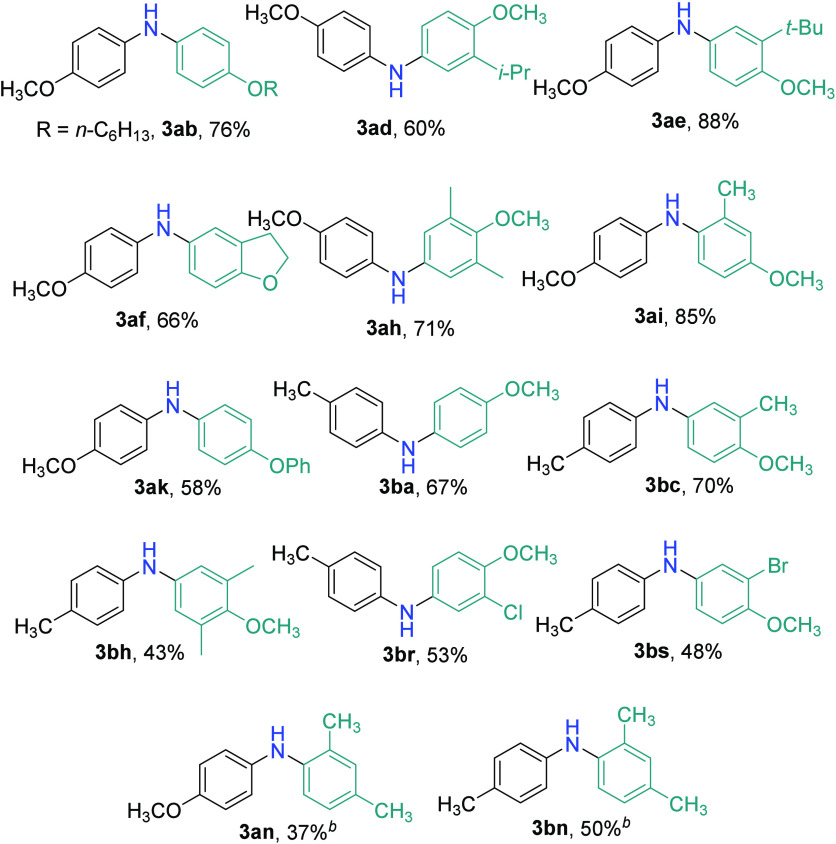
Synthesis of Hetero-Diarylamines^a^

a Anisole (1.0 mmol) was added to a solution of nitrosoarene (1.0 mmol), NOBF_4_ (0.3 mmol), TFA (0.5 mL, 6.5 mmol), and CH_2_Cl_2_ (1.0 mL). After stirring at rt for 2 h under an atmosphere of oxygen (balloon), to the mixture were added iron powder (0.56 g, 10 mmol), acetic acid (1 mL), and water (0.5 mL), and the resultant mixture was stirred at 50 °C for another 3 h.

b *m*-Xylene (2.5 mmol), nitrosoarene (0.5 mmol), NOBF_4_ (0.1 × 3 mmol), TFA (0.5 mL, 6.5 mmol), and dichloromethane (0.25 mL) were applied, and the reaction mixture was stirred at rt for 4 h. The reduction and workup procedures were the same as above.

**Scheme 2:**
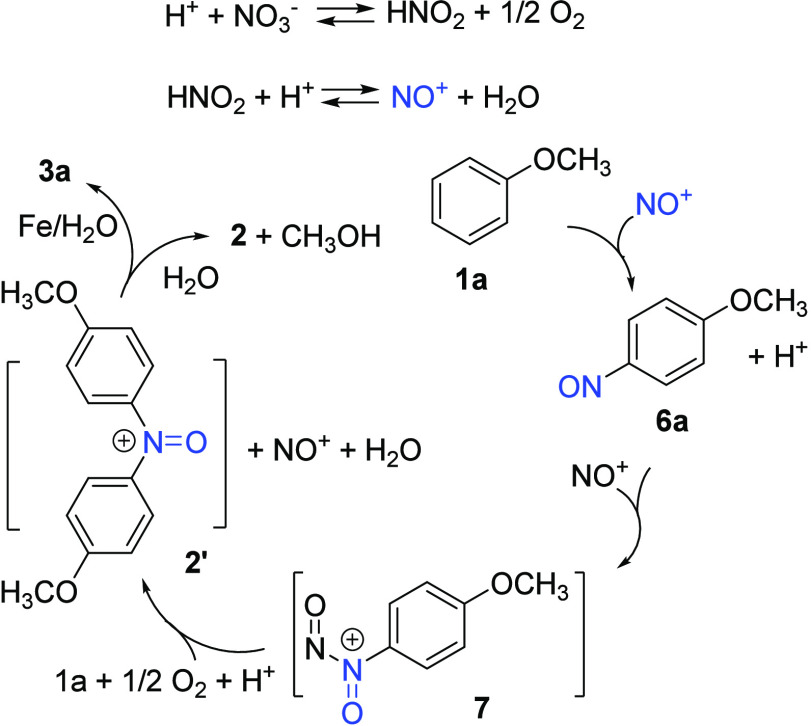
Proposed Reaction Mechanism

The reaction mechanism to account for the formation of diarylamine **3** is shown in Scheme 2. Under the acidic conditions, sodium nitrate, alkyl nitrite, or nitrogen oxides generated NO^+^, which reacted with anisoles to form nitrosoarene **6**.^25,29^ Kochi and Bosch have demonstrated that the affinity of NO^+^ for 4-nitrosoanisole was much higher than that for anisole (*K*_A_/*K*_EDA_ > 20 000) and the structure of adduct **7** was NO^+^ σ-bonded to the nitroso group of **6a**.^25,30^ In this work, adduct **7** further reacts with another anisole to yield diarylaminium *N*-oxide **2**′. The absence of homo-diarylamine **3c** as the product in eq 5 also aligns with the higher affinity of NO^+^ for **6a** than **1c**. To our knowledge, a chemical transformation utilizing the nitroso adduct **7** has not been reported, i.e., the C–N bond formation between nitrosoarenes **6** and arenes **1** is indeed catalyzed by the nitrosonium ion. We propose that NO^+^ is regenerated under an atmosphere of oxygen and acidic conditions. The formation of product **2** or **3a** from intermediate **2′** depended on the isolation/workup procedures. Specifically, in the presence of iron, **2′** was reduced to **3a**, whereas demethylation occurred in the absence of the reducing agent. The high *para*-selectivity relative to the methoxy substituent, as shown in products **3** of Table 2, is consistent with the observed formation of nitrosoarenes, and the unproductive substrates, such as 4-methylanisole and toluene, can be attributed to the lack of formation of the corresponding nitrosoarenes under these conditions.^25^ The low yields of some diarylamines, such as **3h**, in Table 2, could be due to the sluggish formation of the corresponding nitrosoarenes. This nitrosonium catalysis enables broader substrate compatibility and lowers the reaction temperature in the C–N bond formation between nitrosoarenes and arenes.^19c^

## Conclusion

In summary, we have developed a new method to prepare diarylamines via nitrosonium-initiated C–N bond formations. The utilization of dichloromethane as the reaction solvent notably enhanced the formation of aminium *N*-oxide **2′**, which was reduced with iron powder to yield diarylamine. Nitrosoarene is proposed as the reaction intermediate, and its activation by NO^+^ is essential to form the second C–N bond. Thus, the reactions of nitrosonium–nitrosoarene adducts with electron-rich arenes were observed. This protocol utilizes economical, easy to handle and eco-friendly reagents including nitrates/alkyl nitrites as the precursors to NO^+^, oxygen and iron powder as the oxidizing and reducing agents, respectively.^31^ Iron stands as an inexpensive reagent and essential nutrient in coastal seawaters, with its comparative toxicity potential (CTP) designated at zero.^32^ This reaction provides an alternative method to prepare diarylamines.

## Experimental Section

### General Information

Reagents, such as NaNO_3_, NOBF_4_, *tert*-butyl nitrite, sulfuric acid, TFA, and acetic acid, were purchased from commercial sources (ACS grade) and used without further purification. Thin-layer chromatography (TLC) was conducted using precoated silica gel 60 F_254_ plates containing a fluorescent indicator; spots were examined under UV light or revealed by KMnO4 solution. Purification by chromatography was conducted using silica gel (230–400 mesh). The ^1^H and ^13^C{^1^H} NMR spectra were recorded in a CDCl_3_ solution using a Bruker Ascend 600 NMR or Bruker Avance 500, 300 NMR spectrometer. Chemical shifts for ^1^H NMR and ^13^C{^1^H} NMR spectra are reported in δ units (parts per million) with reference to residual solvent peaks. High-resolution mass spectrometry (HRMS) data were recorded on a JMS-700 quadrupole mass spectrometer. The data for single X-ray crystallography were recorded on a Bruker D8 QUEST CCD diffractometer with Mo Kα radiation (*l* = 0.71073 Å), and the structures were solved with the APEX3 program. Nitroso compounds **6a** and **6b** were prepared according to the literature procedure.^33^

#### *N*-(4-Methoxyphenyl)-4-oxocyclohexa-2,5-dien-1-imine oxide (**2**)

Sodium nitrate (85.0 mg, 1.0 mmol) was added to a solution of anisole (216.3 mg, 2.0 mmol) and trifluoroacetic acid (3 mL) at 0 °C (ice–water bath). The reaction mixture was stirred at 0 °C for 5 min and then at rt for 15 h, during which the color of the mixture turned deep purple. Aqueous sodium bicarbonate solution (10% w/w, 30 mL) was added to the reaction mixture, and the organic layer was separated and concentrated. The crude product was purified by column chromatography (SiO_2_, EtOAc/hexanes, 1:1, *R*_*f*_ 0.38) to give compound **2** (57.1 mg, 0.25 mmol, 25%) as an orange solid. Compound **2** was also prepared from the addition of anisole (108.1 mg, 1.0 mmol) to the solution of nitrosoanisole **6a** (121.1 mg, 1.0 mmol), NOBF_4_ (35.0 mg, 0.3 mmol), TFA (trifluoroacetic acid, 0.5 mL), and dichloromethane (1.0 mL). The reaction mixture was stirred at rt for 2 h and diluted with CH_2_Cl_2_ (10 mL). The organic layer was separated, washed with NaOH(aq) (1 M, 15 mL), dried over sodium sulfate, filtered, and concentrated. The product **2** (199.1 mg, 0.87 mmol, 87%) was isolated after column chromatography. Mp 125.5–126.5 °C; ^1^H NMR (300 MHz, CDCl_3_) δ 8.00 (d, *J* = 9.9 Hz, 1H), 7.42 (d, *J* = 9.0 Hz, 2H), 7.25 (d, *J* = 9.9 Hz, 1H), 6.98 (d, *J* = 9.0 Hz, 2H), 6.62 (d, *J* = 9.9 Hz, 1H), 6.24 (d, *J* = 9.9 Hz, 1H), 3.87 (s, 3H); ^13^C{^1^H} NMR (75 MHz, CDCl_3_) δ 186.7, 161.9, 143.1, 138.8, 131.6, 130.9, 129.9, 128.9, 126.3, 114.4, 55.8; HRMS (ESI) *m*/*z* calcd for [M + H]^+^ (C_13_H_12_NO_3_) 230.0805, found 230.0812. Single crystals of **2** suitable for X-ray analysis were grown by slow diffusion between hexanes and a dichloromethane solution of **2**.

#### Bis(4-methoxyphenyl)amine (**3a**)

Sodium nitrate (85.0 mg, 1.0 mmol) was added to a solution of anisole (**1a**, 108.0 mg, 1.0 mmol), dichloromethane (1.0 mL), and trifluoroacetic acid (0.5 mL) at 0 °C. The reaction mixture was stirred at rt for 3 h, then supplemented with iron powder (0.56 g, 10.0 mmol), acetic acid (1.0 mL), and water (0.5 mL) and stirred at 50 °C (oil bath) for another 3 h. After being cooled to rt, the suspension was diluted with dichloromethane (10 mL) and water (10 mL). The organic layer was separated, washed with NaOH(aq) (1 *N*, 15 mL), dried over sodium sulfate, filtered, and concentrated. The crude product was purified by column chromatography (SiO_2_, EtOAc/hexanes, 1:1, *R*_*f*_ 0.83) to give compound **3a** (76.1 mg, 0.33 mmol, 66%) as a deep purple solid. Mp 101.0–103.0 °C; ^1^H NMR (300 MHz, CDCl_3_) δ 6.96 (d, *J* = 9.0 Hz, 4H), 6.84 (d, *J* = 9.0 Hz, 4H), 5.35 (br, 1H), 3.79 (s, 6H); ^13^C{^1^H} NMR (75 MHz, CDCl_3_) δ 154.1, 137.8, 119.4, 114.6, 55.5. The spectroscopic data were consistent with the reported values.^34^ 4-Methoxyaniline (**4a**, 18.4 mg, 0.15 mmol, 15%) was also isolated during column chromatography (SiO_2_, EtOAc/hexanes, 1:1, *R*_*f*_ 0.53) as a colorless solid. Mp 50.0–52.0 °C; ^1^H NMR (500 MHz, CDCl_3_) δ 6.75 (d, *J* = 8.7 Hz, 2H), 6.66 (d, *J* = 8.7 Hz, 4H), 3.75 (s, 3H), 3.42 (br, 2H); ^13^C{^1^H} NMR (126 MHz, CDCl_3_) δ 152.8, 139.9, 116.4, 114.8, 55.7.

#### Bis(4-(hexyloxy)phenyl)amine (**3b**)

The procedure to prepare **3a** was followed. Starting with (hexyloxy)benzene (**1b**, 178.3 mg, 1.0 mmol), compound **3b** (129.3 mg, 0.35 mmol, 70%) was isolated as an orange solid after column chromatography (SiO_2_, EtOAc/hexanes, 1:6, *R*_*f*_ 0.84). Mp 78.0–80.0 °C; ^1^H NMR (300 MHz, CDCl_3_) δ 6.94 (d, *J* = 9.0 Hz, 4H), 6.83 (d, *J* = 9.0 Hz, 4H), 5.28 (s, 1H), 3.93 (t, *J* = 6.7 Hz, 4H), 1.83–1.74 (m, 4H), 1.52–1.43 (m, 4H), 1.38–1.36 (m, 8H), 0.93 (t, *J* = 6.7 Hz, 6H); ^13^C{^1^H} NMR (75 MHz, CDCl_3_) δ 153.7, 137.8, 119.5, 115.4, 68.5, 31.6, 29.3, 25.7, 22.6, 14.0; HRMS (ESI) *m*/*z* calcd for C_24_H_36_NO_2_ (M + H)^+^ 370.2735, found 370.2741.

#### Bis(4-methoxy-3-methylphenyl)amine (**3c**)

The procedure to prepare **3a** was followed. Starting with 2-methylanisole (**1c**, 122.2 mg, 1.0 mmol), compound **3c** (87.5 mg, 0.340 mmol, 68%) was isolated as a yellow liquid after column chromatography (SiO_2_, EtOAc/hexanes, 1:3, *R*_*f*_ 0.72). Mp 66.0–68.0 °C; ^1^H NMR (300 MHz, CDCl_3_) δ 6.83–6.80 (m, 4H), 6.76–6.73 (m, 2H), 5.20 (br, 1H), 3.81 (s, 6H), 2.21 (s, 6H); ^13^C{^1^H} NMR (75 MHz, CDCl_3_) δ 152.4, 137.6, 127.5, 121.4, 116.2, 111.0, 55.8, 16.3. The spectroscopic data were consistent with the reported values.^19a^

#### Bis(3-isopropyl-4-methoxyphenyl)amine (**3d**)

The procedure to prepare **3a** was followed. Starting with 2-isopropylanisole (**1d**, 150.2 mg, 1.0 mmol), compound **3d** (115.9 mg, 0.37 mmol, 74%) was isolated as a colorless solid after column chromatography (SiO_2_, EtOAc/hexanes, 1:4, *R*_*f*_ 0.57). Mp 66.0–68.0 °C; ^1^H NMR (500 MHz, CDCl3) δ 6.94 (d, *J* = 2.3 Hz, 2H), 6.81–6.76 (m, 4H), 5.33 (s, 1H), 3.81 (s, 6H), 3.32 (sept, *J* = 7.0 Hz, 2H), 1.21 (s, 6H), 1.20 (s, 6H); ^13^C{^1^H} NMR (126 MHz, CDCl3) δ 151.3, 138.1, 137.9, 116.7, 115.7, 111.7, 56.0, 26.7, 22.8; HRMS (ESI) *m*/*z* calcd for C_20_H_28_NO_2_ (M + H)^+^ 314.2114, found 314.2115. Single crystals of **3d** suitable for X-ray analysis were grown by slow diffusion between hexanes and a dichloromethane solution of **3d**.

#### Bis(3-(*tert*-butyl)-4-methoxyphenyl)amine (**3e**)

The procedure to prepare **3a** was followed. Starting with 2-*tert*-butylanisole (**1e**, 164.2 mg, 1.0 mmol), compound **3e** (94.9 mg, 0.287 mmol, 56%) was isolated as a colorless solid after column chromatography (SiO_2_, EtOAc/hexanes, 1:10, *R*_*f*_ 0.50). Mp 84.0–86.0 °C; ^1^H NMR (500 MHz, CDCl_3_) δ 7.00 (d, *J* = 2.6 Hz, 2H), 6.86–6.80 (m, 4H), 5.33 (s, 1H), 3.83 (s, 6H), 1.39 (s, 18H); ^13^C{^1^H} NMR (126 MHz, CDCl_3_) δ 153.1, 139.3, 137.4, 117.6, 115.7, 112.7, 55.5, 34.9, 29.7; HRMS (ESI) *m*/*z* calcd for C_22_H_32_NO_2_ (M + H)^+^ 342.2425, found 342.2428.

#### Bis(2,3-dihydrobenzofuran-5-yl)amine (**3f**)

The procedure to prepare **3a** was followed. Starting with 2,3-dihydrobenzofuran (**1f**, 120.2 mg, 1.0 mmol), compound **3f** (72.1 mg, 0.29 mmol, 57%) was isolated as a colorless solid after column chromatography (SiO_2_, EtOAc/hexanes, 1:4, *R*_*f*_ 0.41). Mp 81.0–82.5 °C; ^1^H NMR (300 MHz, CDCl_3_) δ 6.86 (s, 2H), 6.75–6.66 (m, 4H), 5.20 (br, 1H), 4.54 (t, *J* = 8.6 Hz, 4H), 3.16 (t, *J* = 8.6 Hz, 4H); ^13^C{^1^H} NMR (75 MHz, CDCl_3_) δ 154.7, 138.4, 127.9, 118.4, 115.9, 109.4, 71.2, 30.1; HRMS (ESI) *m*/*z* calcd for C_16_H_16_NO_2_ (M + H)^+^ 254.1188, found 254.1176.

#### Di(6-chromanyl)amine (**3g**)

The procedure to prepare **3a** was followed. Starting with chromane (**1g**, 134.2 mg, 1.0 mmol), compound **3g** (90.1 mg, 0.32 mmol, 64%) was isolated as a colorless solid after column chromatography (SiO_2_, EtOAc/hexanes, 1:4, *R*_*f*_ 0.49). Mp 149.0–150.0 °C; ^1^H NMR (300 MHz, CDCl_3_) δ 6.77–6.68 (m, 6H), 5.15 (br, 1H), 4.15 (t, *J* = 5.0 Hz, 4H), 2.73 (t, *J* = 6.5 Hz, 4H), 2.02–1.95 (m, 4H); ^13^C{^1^H} NMR (75 MHz, CDCl_3_) δ 149.4, 137.4, 122.8, 119.4, 118.0, 117.2, 66.4, 25.0, 22.5; HRMS (ESI) *m*/*z* calcd for C_18_H_20_NO_2_ (M + H)^+^ 282.1481, found 282.1489.

#### Bis(4-methoxy-3,5-dimethylphenyl)amine (**3h**)

The procedure to prepare **3a** was followed. Starting with 2,6-dimethylanisole (**1h**, 136.2 mg, 1.0 mmol), compound **3h** (10.6 mg, 0.16 mmol, 32%) was isolated as a colorless solid after column chromatography (SiO_2_, EtOAc/hexanes, 1:4, *R*_*f*_ 0.52). Mp 57.0–59.0 °C; ^1^H NMR (300 MHz, CDCl_3_) δ 6.67 (s, 4H), 5.29 (br, 1H), 3.70 (s, 6H), 2.24 (s, 12H); ^13^C{^1^H} NMR (126 MHz, CDCl_3_) δ 151.2, 139.5, 131.5, 118.2, 59.9, 16.2. The spectroscopic data were consistent with the reported values.^35^

#### Bis(4-methoxy-2-methylphenyl)amine (**3i**)

The procedure to prepare **3a** was followed. Starting with 3-methylanisole (**1i**, 122.2 mg, 1.0 mmol), compound **3i** (65.6 mg, 0.26 mmol, 51%) was isolated as a yellow liquid after column chromatography (SiO_2_, EtOAc/hexanes, 1:6, *R*_*f*_ 0.52). ^1^H NMR (300 MHz, CDCl_3_) δ 6.80–6.66 (m, 6H), 4.77 (s, 1H), 3.79 (s, 6H), 2.24 (s, 6H); ^13^C{^1^H} NMR (75 MHz, CDCl_3_) δ 154.4, 136.5, 129.6, 119.9, 116.5, 111.7, 55.5, 18.0. The spectroscopic data were consistent with the reported values.^19a^

#### Bis(2,4-dimethoxyphenyl)amine (**3j**)

The procedure to prepare **3a** was followed. Starting with 1,3-dimethoxybenzene (**1j**, 138.2 mg, 1.0 mmol), compound **3j** (166.1 mg, 0.57 mmol, 57%) was isolated as a deep purple solid after column chromatography (SiO_2_, EtOAc/hexanes, 1:3, *R*_*f*_ 0.56). Mp 96.0–97.0 °C; ^1^H NMR (300 MHz, CDCl_3_) δ 7.08 (d, *J* = 8.6 Hz, 2H), 6.53 (d, *J* = 2.6 Hz, 2H), 6.42 (dd, *J* = 8.6 Hz, *J* = 2.6 Hz, 2H), 5.82 (br, 1H), 3.86 (s, 6H), 3.79 (s, 6H); ^13^C{^1^H} NMR (75 MHz, CDCl_3_) δ 154.1, 150.4, 127.3, 116.3, 103.6, 99.3, 55.6. The spectroscopic data were consistent with the reported values.^19a^

#### Bis(4-phenoxyphenyl)amine (**3k**)

The procedure to prepare **3a** was followed. Starting with diphenyl ether (**1k**, 170.2 mg, 1.0 mmol), compound **3k** (207.8 mg, 0.59 mmol, 59%) was isolated as a dark liquid after column chromatography (SiO_2_, EtOAc/hexanes, 1:4, *R*_*f*_ 0.53). Mp 100.0–101.0 °C; ^1^H NMR (300 MHz, CDCl_3_) δ 7.35–7.30 (m, 4H), 7.09–6.96 (m,14H), 5.56 (br, 1H); ^13^C{^1^H} NMR (75 MHz, CDCl_3_) δ 158.2, 150.7, 139.6, 129.6, 122.5, 120.6, 119.3, 117.8; HRMS (FAB) *m*/*z* calcd for C_24_H_19_NO_2_ (M)^+^ 353.1416, found 353.1424.

#### Bis(4-(hexyloxy)-3-methylphenyl)amine (**3l**)

The procedure to prepare **3a** was followed. Starting with 1-(hexyloxy)-2-methylbenzene (**1l**, 192.3 mg, 1.0 mmol), compound **3k** (121.3 mg, 0.31 mmol, 61%) was isolated as a black liquid after column chromatography (SiO_2_, EtOAc/hexanes, 1:9, *R*_*f*_ 0.84). ^1^H NMR (300 MHz, CDCl_3_) δ 6.82–6.72 (m, 6H), 5.19 (s, 1H), 3.93 (t, *J* = 6.0 Hz, 4H), 2.21 (s, 6H), 1.82–1.75 (m, 4H), 1.52–1.45 (m, 4H), 1.39–1.34 (m, 8H), 0.96–0.91 (m, 6H); ^13^C{^1^H} NMR (75 MHz, CDCl_3_) δ 151.9, 137.5, 127.9, 121.3, 116.2, 112.3, 68.6, 31.6, 29.4, 25.8, 22.6, 16.3, 14.0; HRMS (ESI) *m*/*z* calcd for C_26_H_40_NO_2_ (M + H)^+^ 398.3031, found 398.3054.

#### Bis(4-(hexyloxy)-2-methylphenyl)amine (**3m**)

The procedure to prepare **3a** was followed. Starting with 1-(hexyloxy)-3-methylbenzene (**1m**, 192.3 mg, 1.0 mmol), compound **3m** (94.3 mg, 0.24 mmol, 47%) was isolated as a red liquid after column chromatography (SiO_2_, EtOAc/hexanes, 1:6, *R*_*f*_ 0.64). ^1^H NMR (300 MHz, CDCl_3_) δ 6.79–6.63 (m, 6H), 4.73 (s, 1H), 3.92 (t, *J* = 6.6 Hz, 4H), 2.22 (s, 6H), 1.81–1.72 (m, 4H), 1.49–1.34 (m, 12H), 0.94–0.89 (m, 6H); ^13^C{^1^H} NMR (75 MHz, CDCl_3_) δ 153.9, 136.4, 129.5, 119.9, 117.2, 112.4, 68.3, 31.6, 29.4, 25.7, 22.6, 18.0, 14.0; HRMS (ESI) *m*/*z* calcd for C_26_H_40_NO_2_ (M + H)^+^ 398.3028, found 398.3053.

#### 4-(Hexyloxy)-*N*-(4-methoxyphenyl)aniline (**3ab**)

Hexyl phenyl ether (**1b**, 178.3 mg, 1.0 mmol) was added to a solution of 1-methoxy-4-nitrosobenzene (**6a**, 137.1 mg, 1.0 mmol), nitrosonium tetrafluoroborate (NOBF_4_, 35.0 mg, 0.30 mmol), TFA (0.5 mL), and dichloromethane (1.0 mL). The reaction was stirred at rt for 2 h under an atmosphere of oxygen, then supplemented with iron powder (0.56 g, 10.0 mmol), acetic acid (1.0 mL), and water (0.5 mL) and stirred at 50 °C (oil bath) for another 3 h. After cooling to rt, the suspension was diluted with dichloromethane (10 mL) and water (10 mL). The organic layer was separated, washed with NaOH(aq) (1 N, 15 mL), dried over sodium sulfate, filtered, and concentrated. The crude product was purified by column chromatography (SiO_2_, EtOAc/hexanes, 1:3, *R*_*f*_ 0.64) to give compound **3ab** (228.0 mg, 0.76 mmol, 76%) as a black solid. Mp 59.0–60.0 °C; ^1^H NMR (300 MHz, CDCl_3_) δ 6.94 (d, *J* = 7.6 Hz, 4H), 6.82 (d, *J* = 8.8 Hz, 4H), 5.27 (s, 1H), 3.92 (t, *J* = 6.5 Hz, 2H), 3.78 (s, 3H), 1.75 (m, 2H), 1.50–1.39 (m, 2H), 1.36–1.33 (m, 4H), 0.91 (t, *J* = 6.7 Hz, 3H); ^13^C{^1^H} NMR (75 MHz, CDCl_3_) δ 154.1, 153.8, 138.0, 137.7, 119.6, 119.4, 115.4, 114.7, 68.5, 55.6, 31.6, 29.3, 25.7, 22.6, 14.0. The spectroscopic data were consistent with the reported values.^36^

#### 4-Methoxy-*N*-(4-methoxyphenyl)-3-methylaniline (**3ac**)

The procedure to prepare **3ab** was followed. Starting with 1-methoxy-4-nitrosobenzene (**6a**, 137.1 mg, 1.0 mmol) and 2-methylanisole (**1c**, 122.2 mg, 1.0 mmol), compound **3ac** (223.6 mg, 0.92 mmol, 92%) was isolated as a dark liquid after column chromatography (SiO_2_, EtOAc/hexanes, 1:3, *R*_*f*_ 0.55). ^1^H NMR (300 MHz, CDCl_3_) δ 6.95 (d, *J* = 9.0 Hz, 2H), 6.85–6.80 (m, 4H), 6.75 (d, *J* = 9.0 Hz, 1H), 5.26 (s, 1H), 3.81 (s, 3H), 3.79 (s, 3H), 2.21 (s, 3H); ^13^C{^1^H} NMR (75 MHz, CDCl_3_) δ 154.1, 152.5, 138.1, 137.4, 127.6, 121.4, 119.4 (2C), 116.2, 114.7 (2C), 111.0, 55.8, 55.6, 16.3. The spectroscopic data were consistent with the reported values.^37^

#### 3-Isopropyl-4-methoxy-*N*-(4-methoxyphenyl)aniline (**3ad**)

The procedure to prepare **3ab** was followed. Starting with 1-methoxy-4-nitrosobenzene (**6a**, 137.1 mg, 1.0 mmol) and 2-isopropylanisole (**1d**, 150.2 mg, 1.0 mmol), compound **3ad** (162.8 mg, 0.60 mmol, 60%) was isolated as a brown liquid after column chromatography (SiO_2_, EtOAc/hexanes, 1:3, *R*_*f*_ 0.60). ^1^H NMR (300 MHz, CDCl_3_) δ 6.98–6.92 (m, 3H), 6.87–6.77 (m, 4H), 5.34 (s, 1H), 3.82 (s, 3H), 3.81 (s, 3H), 3.33 (sept, *J* = 6.9 Hz, 2H), 1.24 (s, 3H), 1.21 (s, 3H); ^13^C{^1^H} NMR (75 MHz, CDCl_3_) δ 153.9, 151.5, 138.2, 138.1, 137.6, 119.1, 117.3, 116.0, 114.7, 111.5, 55.9, 55.6, 26.7, 22.7; HRMS (ESI) *m*/*z* calcd for C_17_H_22_NO_2_ (M + H)^+^ 272.1637, found 272.1645.

#### 3-(*tert*-Butyl)-4-methoxy-*N*-(4-methoxyphenyl)aniline (**3ae**)

The procedure to prepare **3ab** was followed. Starting with 1-methoxy-4-nitrosobenzene (**6a**, 137.1 mg, 1.0 mmol) and 2-*tert*-butylanisole (**1e**, 164.3 mg, 1.0 mmol), compound **3ae** (251.4 mg, 0.88 mmol, 88%) was isolated as a purple liquid after column chromatography (SiO_2_, EtOAc/hexanes, 1:3, *R*_*f*_ 0.53). ^1^H NMR (300 MHz, CDCl_3_) δ 6.97–6.94 (m, 3H), 6.85–6.81 (m, 4H), 5.31 (brs, 1H), 3.82 (s, 3H), 3.79 (s, 3H), 1.37 (s, 9H); ^13^C{^1^H} NMR (75 MHz, CDCl_3_) δ 153.9, 153.3, 139.4, 138.2, 137.1, 119.1, 118.1, 116.1, 114.7, 112.6, 55.6, 55.5, 34.8, 29.7; HRMS (ESI) *m*/*z* calcd for C_18_H_24_NO_2_ (M + H)^+^ 286.1794, found 286.1802.

#### *N*-(4-Methoxyphenyl)-2,3-dihydrobenzofuran-5-amine (**3af**)

The procedure to prepare **3ab** was followed. Starting with 1-methoxy-4-nitrosobenzene (**6a**, 137.1 mg, 1.0 mmol) and 2,3-dihydrobenzofuran **1f** (120.2 mg, 1.0 mmol), compound **3af** (160.5 mg, 0.67 mmol, 67%) was isolated as a brown solid after column chromatography (SiO_2_, EtOAc/hexanes, 1:4, *R*_*f*_ 0.44). Mp 101.5–102.5 °C; ^1^H NMR (300 MHz, CDCl_3_) δ 6.93–6.90 (m, 3H), 6.83–6.68 (m, 4H), 5.25 (brs, 1H), 4.55 (t, *J* = 8.6 Hz, 3H), 3.78 (s, 3H), 3.16 (t, *J* = 8.6 Hz, 2 H); ^13^C{^1^H} NMR (75 MHz, CDCl_3_) δ 154.9, 153.9, 138.5, 137.7, 127.8, 119.0, 118.8, 116.3, 114.7, 109.4, 71.1, 55.6, 30.1; HRMS (ESI) *m*/*z* calcd for C_15_H_15_NO_2_ (M)^+^ 241.1091, found 241.1097.

#### 4-Methoxy-*N*-(4-methoxyphenyl)-2-methylaniline (**3ai**)

The procedure to prepare **3ab** was followed. Starting with 1-methoxy-4-nitrosobenzene (**6a**, 137.1 mg, 1.0 mmol) and 3-methylanisole (**1i**, 122.2 mg, 1.0 mmol), compound **3ai** (205.8 mg, 0.85 mmol, 85%) was isolated as a red liquid after column chromatography (SiO_2_, EtOAc/hexanes, 1:4, *R*_*f*_ 0.52). ^1^H NMR (300 MHz, CDCl_3_) δ 7.02 (d, *J* = 8.6 Hz, 2H), 6.80–6.76 (m, 5H), 5.01 (brs, 1H), 3.79 (s, 3H), 3.78 (s, 3H), 2.23 (s, 3H); ^13^C{^1^H} NMR (75 MHz, CDCl_3_) δ 155.1, 153.6, 139.1, 135.5, 131.2, 121.6, 118.3, 116.4, 114.7, 111.7, 55.6, 55.5, 18.1; HRMS (ESI) *m*/*z* calcd for C_15_H_17_NO_2_ (M + H)^+^ 243.1250, found 243.1254.

#### 4-Methoxy-*N*-(4-phenoxyphenyl)aniline (**3ak**)

The procedure to prepare **3ab** was followed. Starting with 1-methoxy-4-nitrosobenzene (**6a**, 137.1 mg, 1.0 mmol) and diphenylether (**1k**, 170.2 mg, 1.0 mmol), compound **3ak** (169.4 mg, 0.58 mmol, 58%) was isolated as a brown solid after column chromatography (SiO_2_, EtOAc/hexanes, 1:4, *R*_*f*_ 0.52). Mp 70.0–71.5 °C; ^1^H NMR (300 MHz, CDCl_3_) δ 7.35–7.30 (m, 2H), 7.08–7.04 (m, 3H), 7.01–6.86 (m, 8H), 5.45 (s, 1H), 3.81 (s, 3H); ^13^C{^1^H} NMR (75 MHz, CDCl_3_) δ 158.5, 154.9, 149.7, 141.1, 136.4, 129.6, 122.3, 121.2, 120.8, 117.6, 117.5, 114.7, 55.6; HRMS (ESI) *m*/*z* calcd for C_19_H_18_NO_2_ (M + H)^+^ 292.1337, found 292.1332.

#### *N*-(4-methoxyphenyl)-2,4-dimethylaniline (**3an**)

A solution of 1-methoxy-4-nitrosobenzene (**6a**, 137.1 mg, 1.0 mmol), NOBF_4_ (23.4 mg, 0.20 mmol), trifluoroacetic acid (1.0 mL), and dichloromethane (0.5 mL) was stirred at 25 °C for 5 min, then supplemented with *m*-xylene (**1n**, 530.6 mg, 5.0 mmol). The reaction mixture was stirred at 25 °C for 1.5 h, supplemented with another portion of NOBF_4_ (23.4 mg, 0.20 mmol), stirred for another 1.5 h, supplemented with NOBF_4_ (23.4 mg, 0.20 mmol), and stirred for another 1 h. The workup procedure was the sane for that to prepare **3ab**, and compound **3an** (84.1 mg, 0.37 mmol, 37%) was isolated as a yellow liquid after column chromatography (SiO_2_, EtOAc/hexanes, 1:9, *R*_*f*_ 0.47). ^1^H NMR (300 MHz, CDCl_3_) δ 7.00 (s, 1H), 6.95–6.93 (m, 4H), 6.85 (d, *J* = 8.5 Hz, 2H), 5.12 (s, 1H), 3.80 (s, 3H), 2.28 (s, 3H)), 2.23 (s, 3H); ^13^C{^1^H} NMR (75 MHz, CDCl_3_) δ 154.4, 140.4, 137.4, 131.5, 130.1, 127.2, 120.6, 117.0, 115.2, 114.6, 55.6, 20.5, 17.7. The spectroscopic data were consistent with the reported values.^38^

#### 4-Methoxy-*N*-(*p*-tolyl)aniline (**3ba**)

The procedure to prepare **3ab** was followed. Starting with 1-methyl-4-nitrosobenzene (**6b**, 121.0 mg, 1.0 mmol) and anisole (**1a**, 108.1 mg, 1.0 mmol), compound **3ba** (141.8 mg, 0.67 mmol, 67%) was isolated as a brown solid after column chromatography (SiO_2_, EtOAc/hexanes, 1:4, *R*_*f*_ 0.65). Mp 73.0–74.5 °C; ^1^H NMR (300 MHz, CDCl_3_) δ 7.05–7.01 (m, 4H), 6.87–6.83 (m, 4H), 5.40 (brs, 1H), 3.79 (s, 3H), 2.28 (s, 3H); ^13^C{^1^H} NMR (75 MHz, CDCl_3_) δ 154.7, 142.4, 136.6, 129.8, 129.3, 121.1, 116.5, 114.6, 55.6, 20.5. The spectroscopic data were consistent with the reported values.^39^

#### 4-Methoxy-3-methyl-*N*-(*p*-tolyl)aniline (**3bc**)

The procedure to prepare **3ab** was followed. Starting with 1-methyl-4-nitrosobenzene (**6b**, 121.0 mg, 1.0 mmol) and 2-methylanisole (**1c**, 122.1 mg, 1.0 mmol), compound **3bc** (160.0 mg, 0.70 mmol, 70%) was isolated as a dark liquid after column chromatography (SiO_2_, EtOAc/hexanes, 1:4, *R*_*f*_ 0.76). ^1^H NMR (300 MHz, CDCl_3_) δ 7.04 (d, *J* = 8.2 Hz, 2H), 6.91–6.84 (m, 4H), 6.77–6.74 (m, 1H), 5.36 (brs, 1H), 3.81 (s, 3H), 2.28 (s, 3H), 2.20 (s, 3H); ^13^C{^1^H} NMR (75 MHz, CDCl_3_) δ 153.0, 142.5, 136.1, 129.7, 129.1, 127.5, 122.9, 117.8, 116.5, 110.9, 55.7, 20.5, 16.3; HRMS (ESI) *m*/*z* calcd for C_15_H_18_NO (M + H)^+^ 228.1382, found 228.1383.

#### 4-Methoxy-3,5-dimethyl-*N*-(*p*-tolyl)aniline (**3bh**)

The procedure to prepare **3ab** was followed. Starting with 1-methyl-4-nitrosobenzene (**6b**, 121.0 mg, 1.0 mmol) and 2,6-dimethylanisole (**1h**, 136.2 mg, 1.0 mmol), compound **3bh** (102.6 mg, 0.43 mmol, 43%) was isolated as a red liquid after column chromatography (SiO_2_, EtOAc/hexanes, 1:4, *R*_*f*_ 0.77). ^1^H NMR (300 MHz, CDCl_3_) δ 7.08 (d, *J* = 8.3 Hz, 2H), 6.94 (d, *J* = 8.3 Hz, 2H), 6.71 (s, 2H), 5.41 (s, 1H), 3.71 (s, 3H), 2.31 (s, 3H), 2.25 (s, 6H); ^13^C{^1^H} NMR (75 MHz, CDCl_3_) δ 151.1, 141.2, 139.3, 131.5, 130.0, 129.8, 118.0, 117.9, 59.9, 20.6, 16.2; HRMS (EI) *m*/*z* calcd for C_16_H_19_NO (M)^+^ 241.1467, found 241.1468.

#### 3-Chloro-4-methoxy-*N*-(*p*-tolyl)aniline (**3br**)

The procedure to prepare **3ab** was followed. Starting with 1-methyl-4-nitrosobenzene (**6b**, 121.0 mg, 1.0 mmol) and 2-chloroanisole (**1r**, 142.6 mg, 1.0 mmol), compound **3br** (131.3 mg, 0.53 mmol, 53%) was isolated as a colorless solid after column chromatography (SiO_2_, EtOAc/hexanes, 1:4, *R*_*f*_ 0.63). Mp 68.0–69.0 °C; ^1^H NMR (300 MHz, CDCl_3_) δ 7.11–7.06 (m, 3H), 6.93–6.83 (m, 4H), 5.41 (brs, 1H), 3.87 (s, 3H), 2.30 (s, 3H); ^13^C{^1^H} NMR (75 MHz, CDCl_3_) δ 149.7, 141.1, 137.7, 130.4, 129.9, 123.0, 120.7, 117.8, 117.6, 113.3; HRMS (EI) *m*/*z* calcd for C_14_H_14_ClNO (M)^+^ 247.0764, found 247.0768.

#### 3-Bromo-4-methoxy-*N*-(*p*-tolyl)aniline (**3bs**)

The procedure to prepare **3ab** was followed. Starting with 1-methyl-4-nitrosobenzene (**6b**, 121.0 mg, 1.0 mmol) and 2-bromoanisole (**1s**, 187.0 mg, 1.0 mmol), compound **3bs** (139.0 mg, 0.48 mmol, 48%) was isolated as a dark liquid after column chromatography (SiO_2_, EtOAc/hexanes, 1:4, *R*_*f*_ 0.67). ^1^H NMR (300 MHz, CDCl_3_) δ 7.29–7.28 (m, 1H), 7.07 (d, *J* = 8.3 Hz, 2H), 6.97 (dd, *J* = 8.7 Hz, *J* = 2.6, Hz, 1H), 6.89 (d, *J* = 8.3 Hz, 2H), 6.84–6.81 (m, 1H), 5.41 (brs, 1H), 3.86 (s, 3H), 2.30 (s, 3H); ^13^C{^1^H} NMR (75 MHz, CDCl_3_) δ 150.7, 141.1, 138.0, 130.4, 129.9, 123.8, 118.7, 117.5, 113.0, 112.1, 56.7, 20.6; HRMS (FAB) *m*/*z* calcd for C_14_H_15_NOBr (M + H)^+^ 292.0332, found 292.0331.

#### 2,4-Dimethyl-*N*-(*p*-tolyl)aniline **(3bn)**

A solution of 1-methyl-4-nitrosobenzene (**6b**, 121.4 mg, 1.0 mmol), NOBF_4_ (23.4 mg, 0.20 mmol), trifluoroacetic acid (1.0 mL,) and dichloromethane (0.5 mL) was stirred at 25 °C for 5 min, then supplemented with *m*-xylene (**1n**, 530.6 mg, 5.0 mmol). The reaction mixture was stirred at 25 °C for 1.5 h, supplemented with another portion of NOBF_4_ (23.4 mg, 0.20 mmol), stirred for another 1.5 h, supplemented with NOBF_4_ (23.4 mg, 0.20 mmol) and stirred for another 1 h. The workup procedure was the same for that to prepare **3ab**, and compound **3bn** (105.7 mg, 0.50 mmol, 50%) was isolated as a yellow liquid after column chromatography (SiO_2_, EtOAc/hexanes, 1:9, *R*_*f*_ 0.57). ^1^H NMR (300 MHz, CDCl_3_) δ 7.11–7.03 (m, 4H), 6.96 (d, *J* = 8.0 Hz, 1H), 6.84 (d, *J* = 8.4 Hz, 1H), 5.22 (s, 1H), 2.31 (s, 6H), 2.23 (s, 3H); ^13^C{^1^H} NMR (75 MHz, CDCl_3_) δ 142.0, 139.0, 131.5, 131.3, 129.7, 129.4, 128.5, 127.2, 119.2, 117.3, 20.6, 20.6, 17.8. The spectroscopic data were consistent with the reported values.^40^

## Data Availability

The data underlying this study are available in the published article and its Supporting Information.

## Supporting Information Available

Diarylamine formation using NaNO_3_ and NO_*x*_, crystal data and structure refinement for **2** and **3d**, and NMR spectra for **2** and synthetic diarylamines (PDF)
FAIR data, including the primary NMR FID files, for compounds **2**, **3a**, **3ab**–**3af**, **3ah**, **3ai**, **3ak**, **3an**, **3b**, **3ba**, **3bc**, **3bh**, **3bn**, **3br**, **3bs**, **3c**–**3m**, and **4a** (ZIP)
