# Supplementary material for: Synthesis of Diarylamines via Nitrosonium-Initiated C–N Bond Formation
Source: J Org Chem. 2024 Jul 1;89(14):10316–26. doi: 10.1021/acs.joc.4c01220 (PMC11267615; doi:10.1021/acs.joc.4c01220)
Supplement: Supplementary file 1 — jo4c01220_si_001.pdf [file jo4c01220_si_001.pdf]

Supporting Information

Synthesis of Diarylamines *via* Nitrosonium Initiated C-N Bond Formation

Pin-Hsien Chen,[§] Shu-Jung Hsu,[§] Cheng-Chun Chen, Jui-Chen Fu and Duen-Ren Hou*

Department of Chemistry, National Central University, No. 300 Jhong-Da Rd., Jhong-li, Taoyuan, Taiwan, 320317.

Table of Contents

Table S1. Diarylamine Formation using $NaNO_3$……………………………….S5

Table S2. Diarylamine Formation using NO_x…………………………………..S6

Table S3. Crystal data and structure refinement for **2** (CCDC 2322222).......S7

Table S4. Crystal data and structure refinement for **3d** (CCDC 2322240).....S9

Figure S1. Oak Ridge thermal ellipsoid plot of **3d**..S11

^{1}H NMR of compound **2**…..S12

^{13}C{^{1}H} NMR of compound **2**…...…S13

^{1}H NMR of compound **3a**..S14

^{13}C{^{1}H} NMR of compound **3a**..S15

^{1}H NMR of compound **4a**..S16

^{13}C{^{1}H} NMR of compound **4a**..S17

^{1}H NMR of compound **3b**..S18

^{13}C{^{1}H} NMR of compound **3b**..S19

^{1}H NMR of compound **3c**..S20

^{13}C{^{1}H} NMR of compound **3c**..S21

^{1}H NMR of compound **3d**..S22

^{13}C{^{1}H} NMR of compound **3d**..S23

^{1}H NMR of compound **3e**..S24

^{13}C{^{1}H} NMR of compound **3e**..S25

^{1}H NMR of compound **3f**..S26

^{13}C{^{1}H} NMR of compound **3f**..S27

^{1}H NMR of compound **3g**..S28

^{13}C{^{1}H} NMR of compound **3g**..S29

^{1}H NMR of compound **3h**..S30
^{13}C{^{1}H} NMR of compound **3h**..S31
^{1}H NMR of compound **3i**..S32
^{13}C{^{1}H} NMR of compound **3i**..S33
^{1}H NMR of compound **3j**..S34
^{13}C{^{1}H} NMR of compound **3j**..S35
^{1}H NMR of compound **3k**..S36
^{13}C{^{1}H} NMR of compound **3k**..S37
^{1}H NMR of compound **3l**..S38
^{13}C{^{1}H} NMR of compound **3l**..S39
^{1}H NMR of compound **3m**..S40
^{13}C{^{1}H} NMR of compound **3m**..S41
^{1}H NMR of compound **3ab**..S42
^{13}C{^{1}H} NMR of compound **3ab**..S43
^{1}H NMR of compound **3ac**..S44
^{13}C{^{1}H} NMR of compound **3ac**..S45
^{1}H NMR of compound **3ad**..S46
^{13}C{^{1}H} NMR of compound **3ad**..S47
^{1}H NMR of compound **3ae**..S48
^{13}C{^{1}H} NMR of compound **3ae**..S49
^{1}H NMR of compound **3af**..S50
^{13}C{^{1}H} NMR of compound **3af**..S51
^{1}H NMR of compound **3ai**..S52
^{13}C{^{1}H} NMR of compound **3ai**..S53
^{1}H NMR of compound **3ak**..S54

$^{13}C\{^1H\}$ NMR of compound **3ak**..S55
^1H NMR of compound **3an**..S56
$^{13}C\{^1H\}$ NMR of compound **3an**..S57
^1H NMR of compound **3ba**..S58
$^{13}C\{^1H\}$ NMR of compound **3ba**..S59
^1H NMR of compound **3bc**..S60
$^{13}C\{^1H\}$ NMR of compound **3bc**..S61
^1H NMR of compound **3bh**..S62
$^{13}C\{^1H\}$ NMR of compound **3bh**..S63
^1H NMR of compound **3br**..S64
$^{13}C\{^1H\}$ NMR of compound **3br**..S65
^1H NMR of compound **3bs**..S66
$^{13}C\{^1H\}$ NMR of compound **3bs**..S67
^1H NMR of compound **3bn**..S68
$^{13}C\{^1H\}$ NMR of compound **3bn**..S69

Table S1. Diarylamine Formation using $NaNO_3$[a]

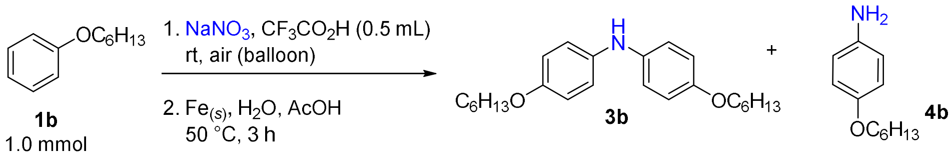

entry	equiv of $NaNO_3$	solvent	time (h)	**3b** (mmol)/ yield (%)	**4b** (mmol)	recovered **1b** (mmol)
1	0.5	TFA (1.5 mL)	1	0.18/ 36	0.10	0.20
2	0.5	TFA (1.5 mL)	3	0.19/ 38	0.09	0.13
3	0.5	TFA (1.5 mL)	12	0.16/ 32	0.05	0.12
4	0.5	TFA (1.5 mL)	40	0.11/ 22	0.09	0.11
4	0.5[b]	TFA (1.5 mL)	2	0.21/ 42	0.12	N.D.[c]
5	0.7	TFA (1.5 mL)	3	0.15/ 30	0.10	N.D.[c]
6	1.0	TFA (1.5 mL)	3	0.18/ 36	0.15	0.03
7	0.5	CH_2Cl_2 (1.0 mL)	3	0.29/ 58	0.08	0.30
8	0.75	CH_2Cl_2 (1.0 mL)	3	0.28/ 56	0.09	N.D.[c]
9	1.0	CH_2Cl_2 (1.0 mL)	3	0.35/ 70	0.12	N.D.[c]
10	1.0	CH_2Cl_2 (1.0 mL)	1	0.30/ 60	0.11	N.D.[c]
11	1.25	CH_2Cl_2 (1.0 mL)	3	0.23/ 46	0.12	N.D.[c]
12	1.0	1,2-dichloroethane (1.0 mL)	3	0.34/ 68	0.10	N.D.[c]
13	1.0	CH_3CO_2H (1.0 mL)	3	0.20/ 40	0.12	N.D.[c]
14	1.0	CH_3CN (1.0 mL)	5	0.22/ 44	0.22	0.13
15	1.0	toluene (1.0 mL)	16	0.19/ 38	0.06	N.D.
16	1.0	acetone (1.0 mL)	16	0.16/ 32	0.04	0.42
17	1.0	EA (1.0 mL)	16	0.04/ 8	0.01	0.68
18	1.0	methanol (1.0 mL)	16	N.D.[c]	N.D.[c]	0.96
19	1.0	Et_2O (1.0 mL)	16	N.D.[c]	N.D.[c]	0.94
20	1.0	THF (1.0 mL)	16	N.D.[c]	N.D.[c]	0.95
21	1.0	DMF (1.0 mL)	16	N.D.[c]	N.D.[c]	0.88

[a] Sodium nitrate (1.0 mmol) was added to a solution of **1b** (178.3 mg, 1.0 mmol), TFA (0.5 mL, 0.74 g, 6.5 mmol) and a solvent (1.0 mL) at 0 °C. After stirred at rt under an atmosphere of air (balloon), the reaction mixture was added with iron powder (0.56 g, 10 mmol), acetic acid (1 mL), water (0.5 mL), and stirred at 50 °C for another 3 h. The amounts of **1b**, **3b** and **4b** were determined by ^{1}H NMR using 1,4-dimethoxybenzene as an internal standard. [b]$NaNO_3$ was added in five separate portions at 0 °C, with a 15-minute interval between each addition. [c]Not dectected.

Table S2. Diarylamine Formation using NO_x[a]

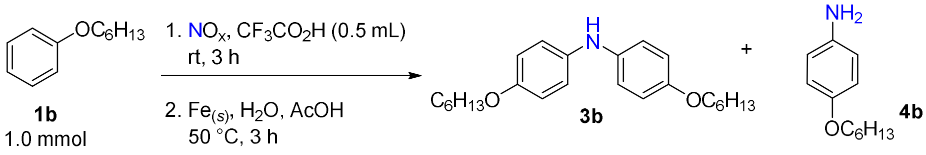

Entry	NO_x	**1b** (mmol) : NO_x (mmol)	atmosphere (balloon)	**3b** (mmol)/ yield (%)[b]	**4b** (mmol)	**1b** (mmol)
1	$NOBF_4$	1.5 : 0.5	Air	0.25/ 50	0.03	0.87
2	$NOBF_4$	1.5 : 0.5	N_2	0.08/ 16	0.03	0.71
3	$NOBF_4$	1.5 : 0.5	O_2	0.31/ 62	0.04	0.68
4	$NOBF_4$	1.5 : 0.5	N_2 (3 h), then O_2 (3 h)	0.21/ 42	0.04	0.66
5	$NOBF_4$	1.0 : 0.5	O_2	0.35/ 70	0.08	0.03
6	$NOBF_4$	1.0 : 1.0	O_2	0.32/ 64	0.03	N. D.
7	$NOBF_4$[c]	1.5 : 0.5	O_2	0.02/ 4	N.D.	0.88
8	*tert*-butyl nitrite	1.5 : 0.5	O_2	0.34/ 68	0.03	0.69
9	NO_2BF_4	1.5 : 0.5	Air	0.16/ 32	0.12	0.75
10	NO_2BF_4	1.5 : 0.5	N_2	0.08/ 16	0.10	0.67
11	NO_2BF_4	1.5 : 0.5	O_2	0.30/ 60	0.13	0.39
12	$NaNO_3$	1.5 : 0.5	O_2	0.13/ 25	0.06	0.77

[a]The amounts of **1b**, **3b** and **4b** were determined by ^{1}H NMR using 1,4-dimethoxybenzene as an internal standard. [b]Yield based on NO_x. [c]Without TFA.

Table S3. Crystal structure and refinement data for compound **2 (CCDC 2322222)**.

Identification code	22MAR02_1_0m	
Empirical formula	C26 H22 N2 O6	
Formula weight	458.45	
Temperature	296(2) K	
Wavelength	0.71073 Å	
Crystal system	Monoclinic	
Space group	$P2_1/c$	
Unit cell dimensions	a = 15.0661(4) Å	α= 90°.
	b = 7.0003(2) Å	β= 93.7100(10)°.
	c = 21.6200(7) Å	γ = 90°.
Volume	2275.42(12) $Å^3$	
Z	4	
Density (calculated)	1.338 Mg/m^3	
Absorption coefficient	0.096 mm^{-1}	
F(000)	960	
Crystal size	0.256 x 0.124 x 0.062 mm^3	
Theta range for data collection	2.251 to 28.365°.	
Index ranges	-20<=h<=20, -9<=k<=9, -28<=l<=28	
Reflections collected	42321	
Independent reflections	5673 [R(int) = 0.0987]	
Completeness to theta = 25.242°	99.9 %	
Absorption correction	Numerical Mu Calculated	
Max. and min. transmission	0.7457 and 0.5632	
Refinement method	Full-matrix least-squares on F^2	

Data / restraints / parameters	5673 / 0 / 309
Goodness-of-fit on F^2	1.025
Final R indices [I>2sigma(I)]	R_1 = 0.0725, wR_2 = 0.1750
R indices (all data)	R_1 = 0.1481, wR_2 = 0.2187
Extinction coefficient	n/a
Largest diff. peak and hole	0.329 and -0.319 e.Å^{-3}

Table S4. Crystal data and structure refinement for **3d (CCDC 2322240)**.

Crystal data and structure refinement for	22DEC03_1_0m.	
Identification code	22DEC03_1_0m	
Empirical formula	C20 H27 N O2	
Formula weight	313.42	
Temperature	296(2) K	
Wavelength	0.71073 Å	
Crystal system	Monoclinic	
Space group	$P2_1/c$	
Unit cell dimensions	a = 8.30370(10) Å	α= 90°.
	b = 15.9230(3) Å	β= 90.8810(10)°.
	c = 13.8948(2) Å	γ = 90°.
Volume	1836.95(5) Å^3	
Z	4	
Density (calculated)	1.133 Mg/m^3	
Absorption coefficient	0.072 mm^{-1}	
F(000)	680	
Crystal size	0.249 x 0.160 x 0.093 mm^3	
Theta range for data collection	2.453 to 28.323°.	
Index ranges	-11<=h<=11, -21<=k<=21, -18<=l<=18	
Reflections collected	45952	
Independent reflections	4559 [R(int) = 0.0697]	
Completeness to theta = 25.242°	99.6 %	
Absorption correction	Numerical Mu Calculated	

Max. and min. transmission	0.7457 and 0.6654
Refinement method	Full-matrix least-squares on F^2
Data / restraints / parameters	4559 / 0 / 214
Goodness-of-fit on F^2	1.036
Final R indices [I>2sigma(I)]	R_1 = 0.0546, wR_2 = 0.1442
R indices (all data)	R_1 = 0.0831, wR_2 = 0.1654
Extinction coefficient	n/a
Largest diff. peak and hole	0.274 and -0.201 e.Å^{-3}

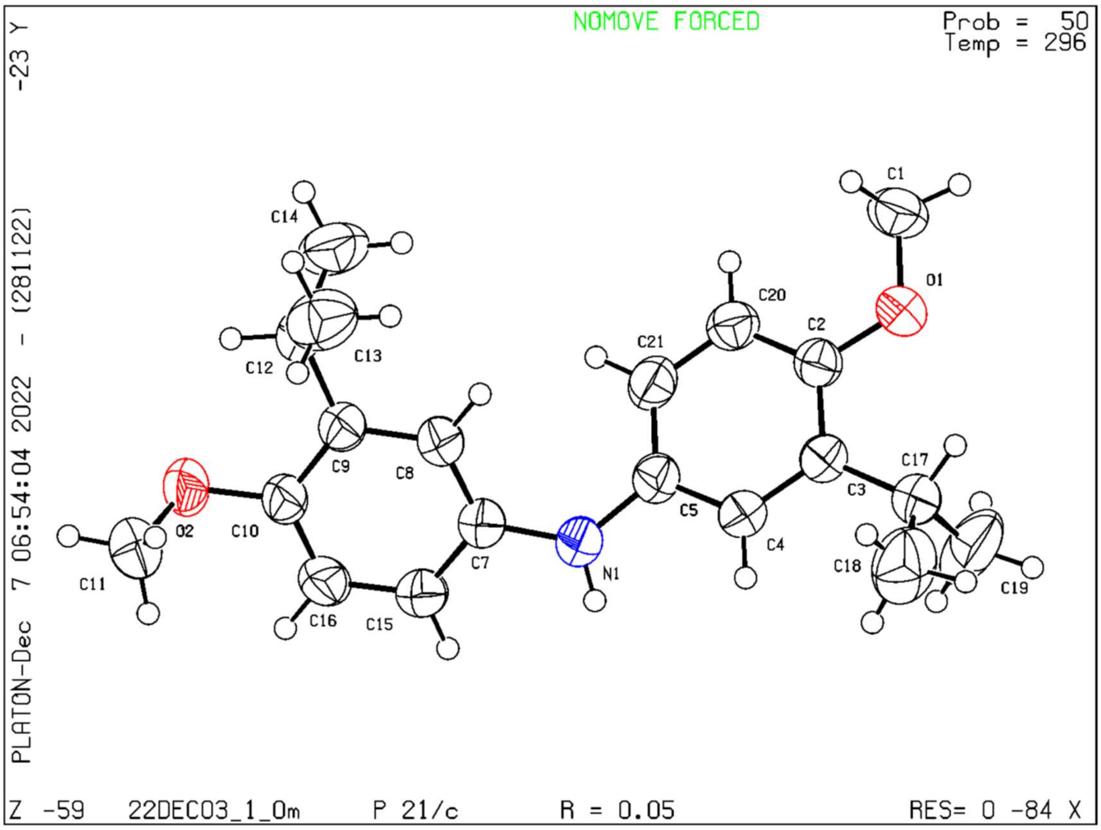

Figure S1. Oak Ridge thermal ellipsoid plot of **3d** with ellipsoids set to 50% probability.

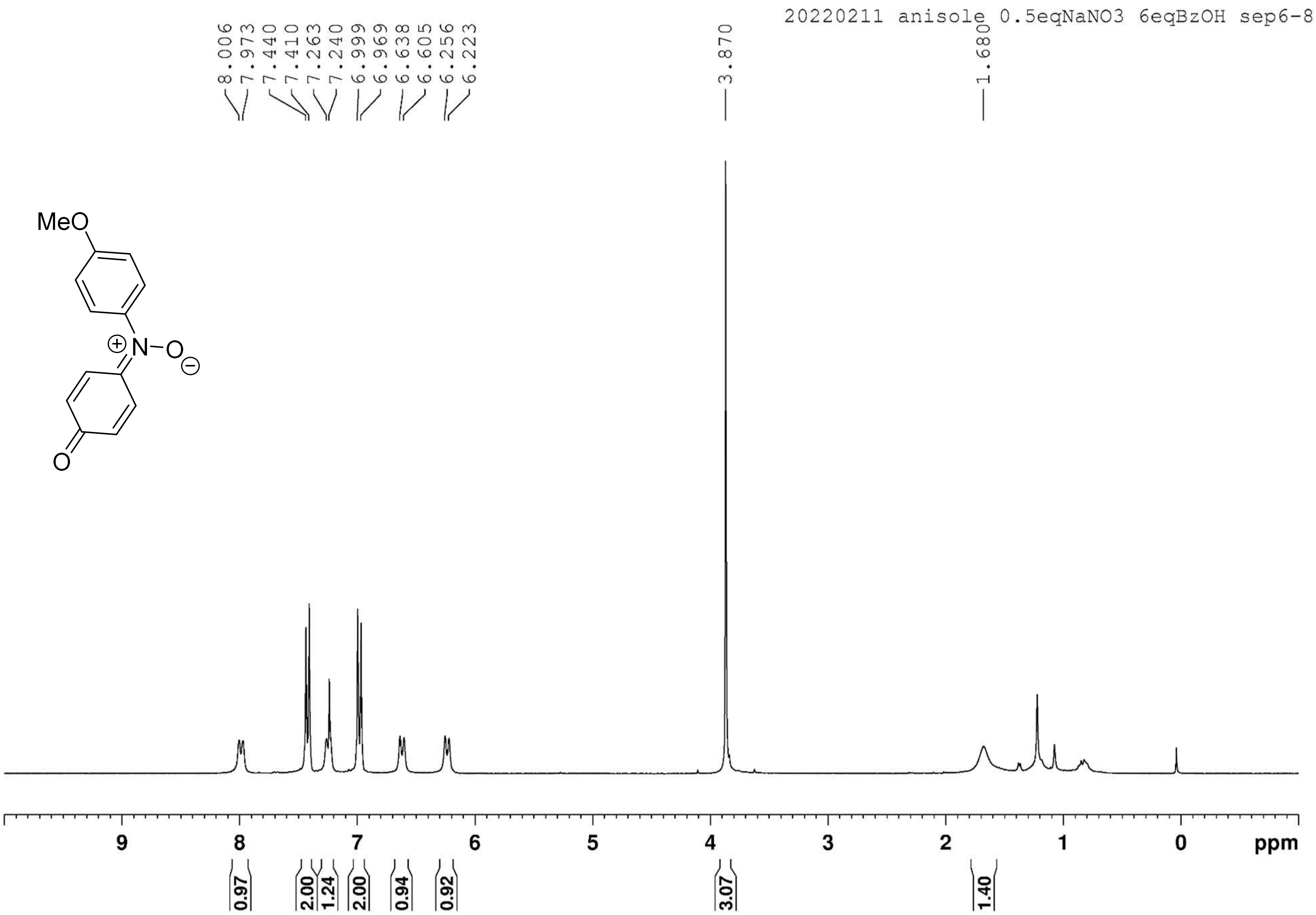

^{1}H NMR of compound **2** (300 MHz, $CDCl_3$)

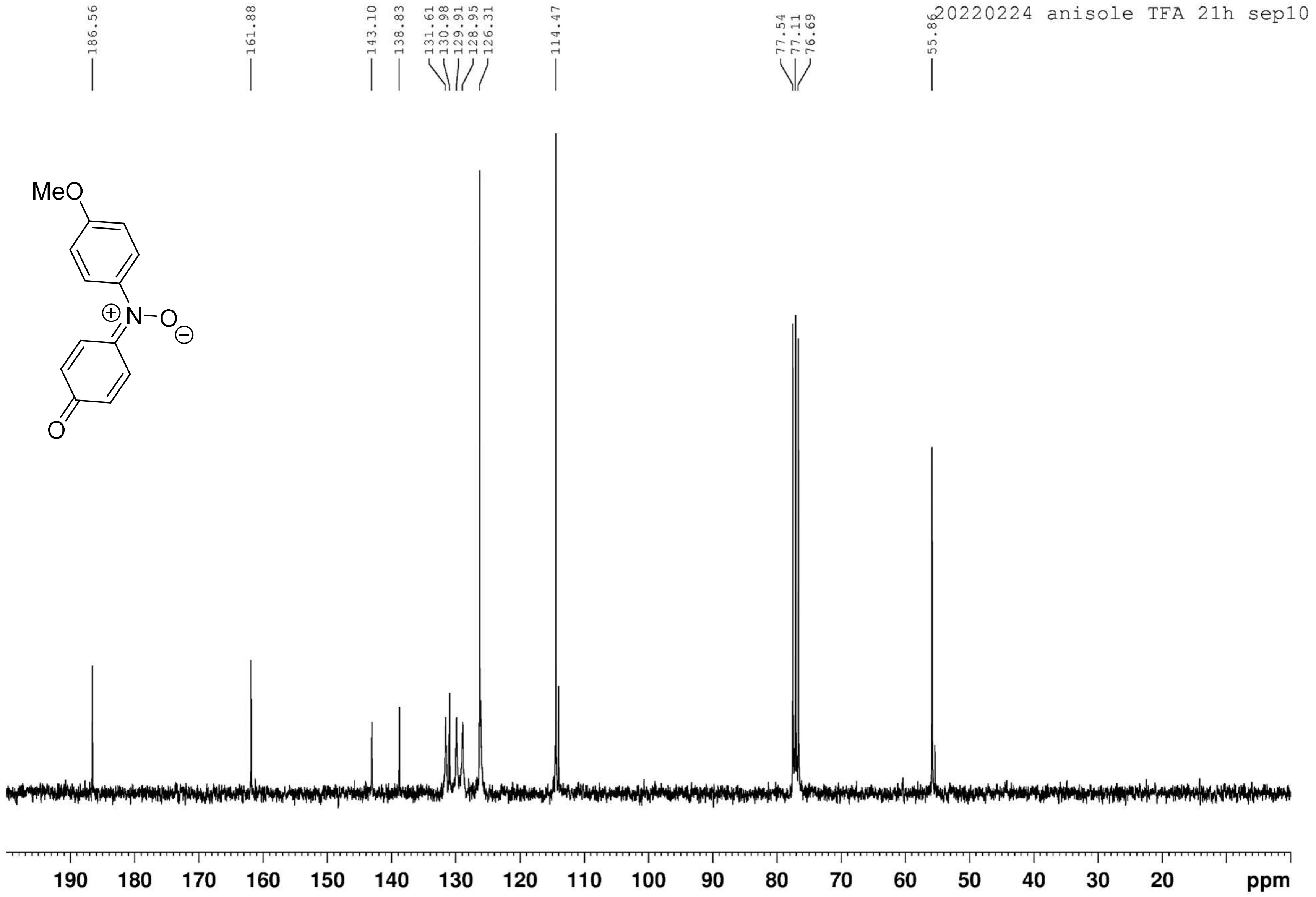

$^{13}C\{^1H\}$ NMR of compound **2** (75 MHz, $CDCl_3$)

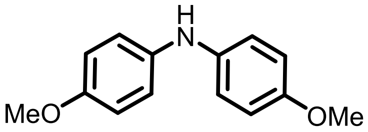

3a

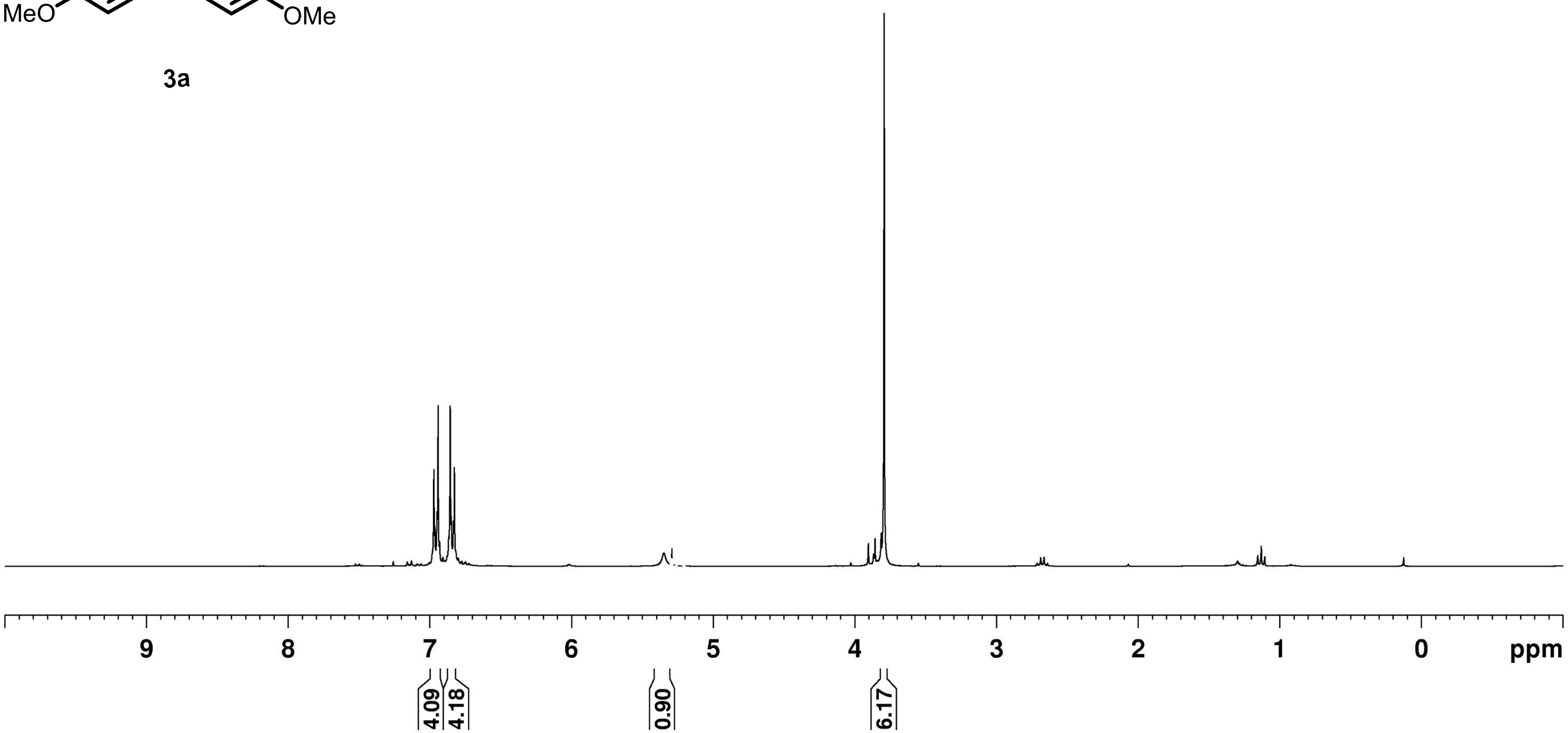

^{1}H NMR of compound **3a** (300 MHz, $CDCl_3$)

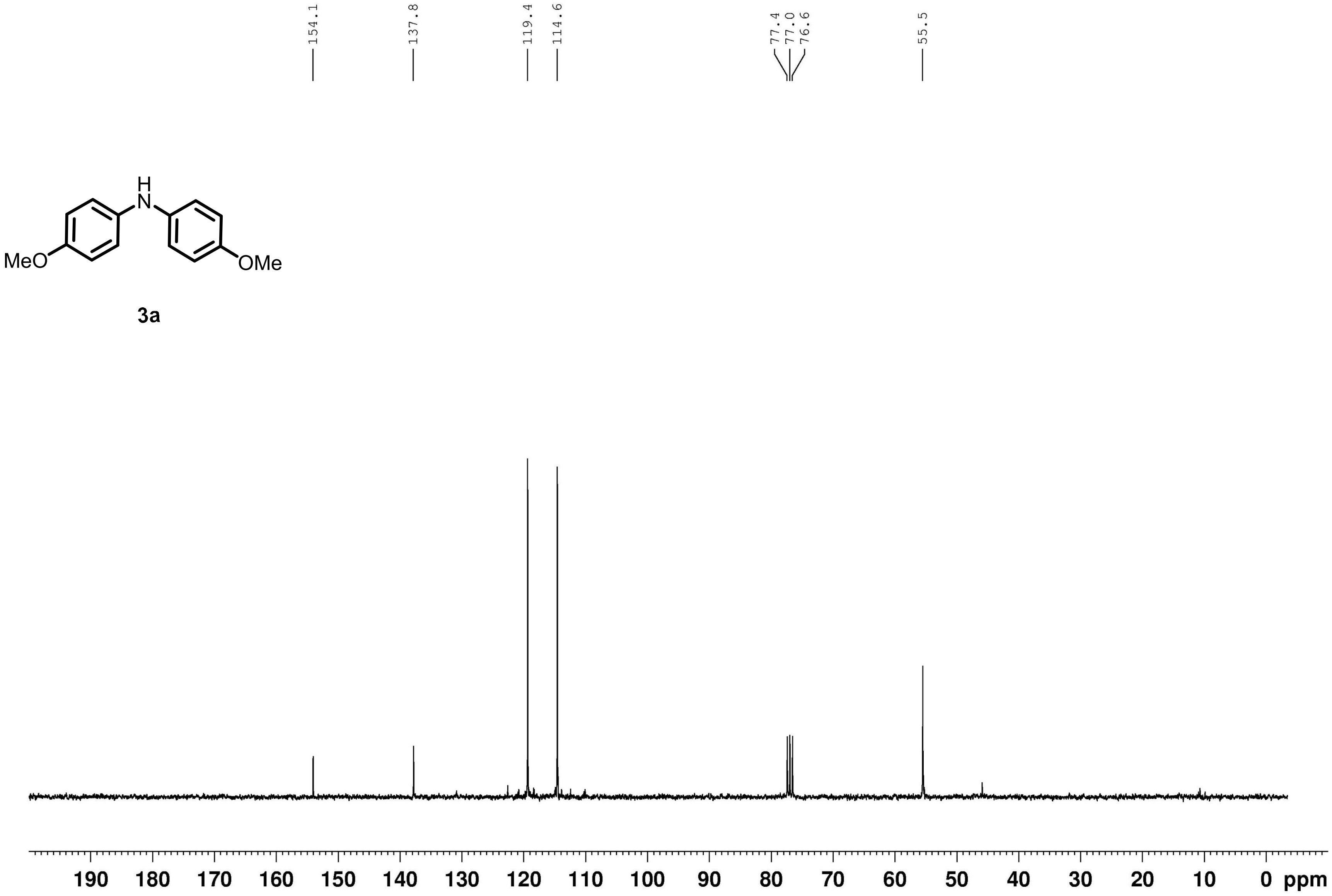

$^{13}C\{^1H\}$ NMR of compound **3a** (75 MHz, $CDCl_3$)

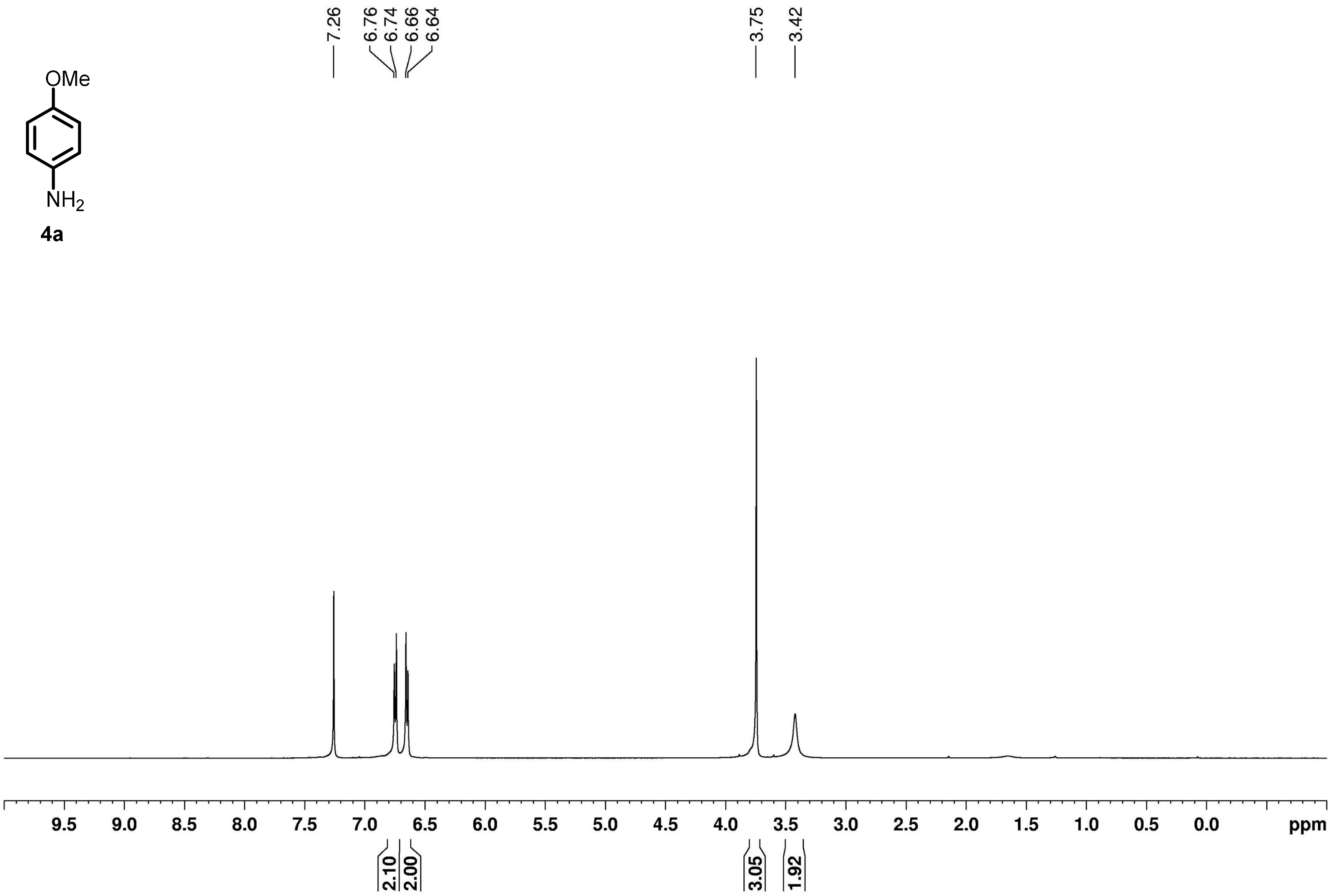

^{1}H NMR of compound **4a** (500 MHz, $CDCl_3$)

5

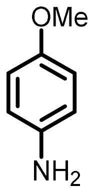

4a

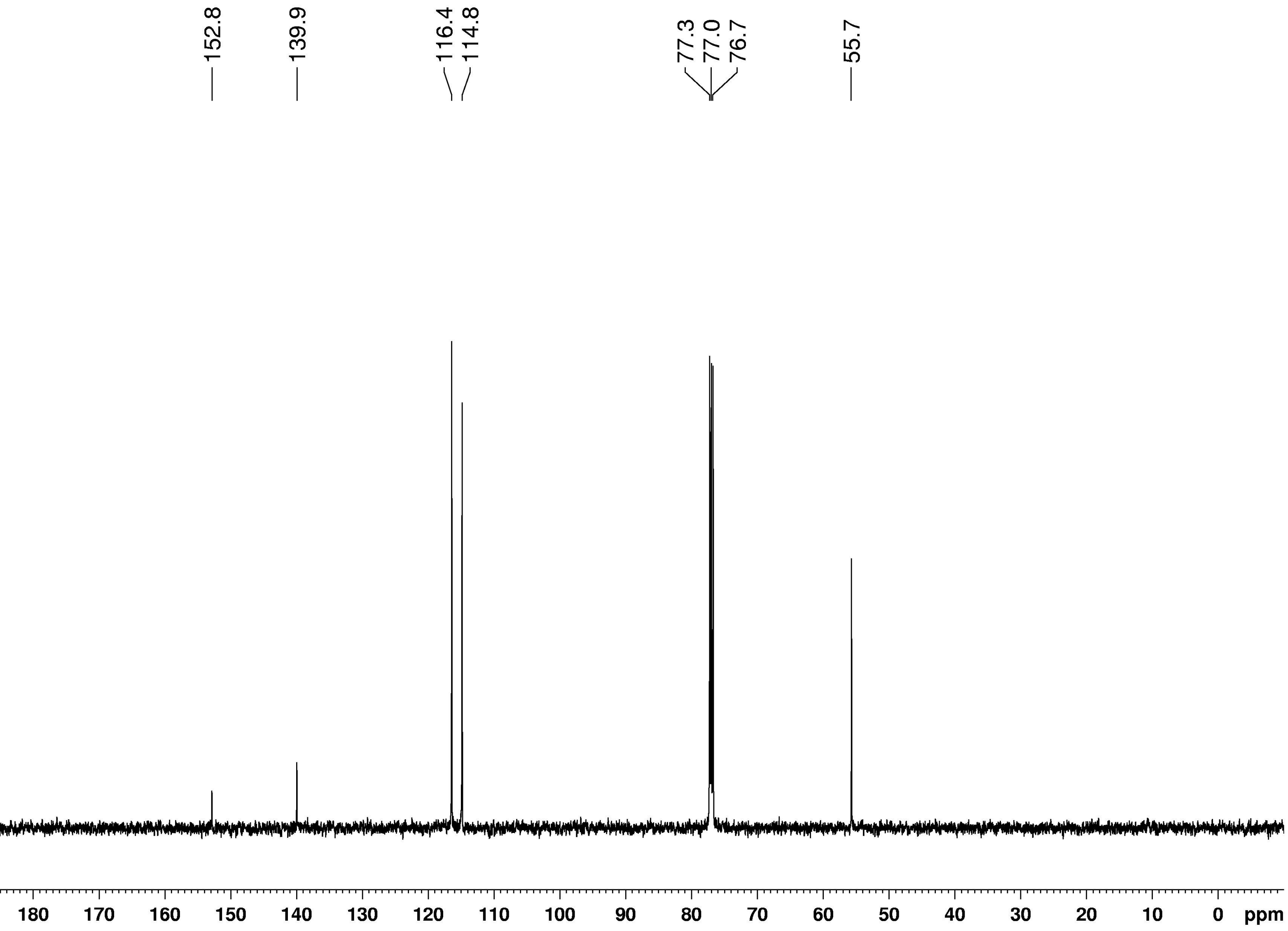

$^{13}C\{^{1}H\}$ NMR of compound **4a** (126 MHz, $CDCl_3$)

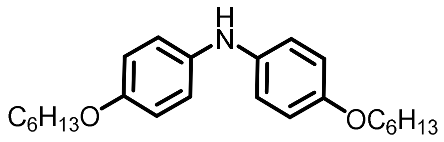

3b

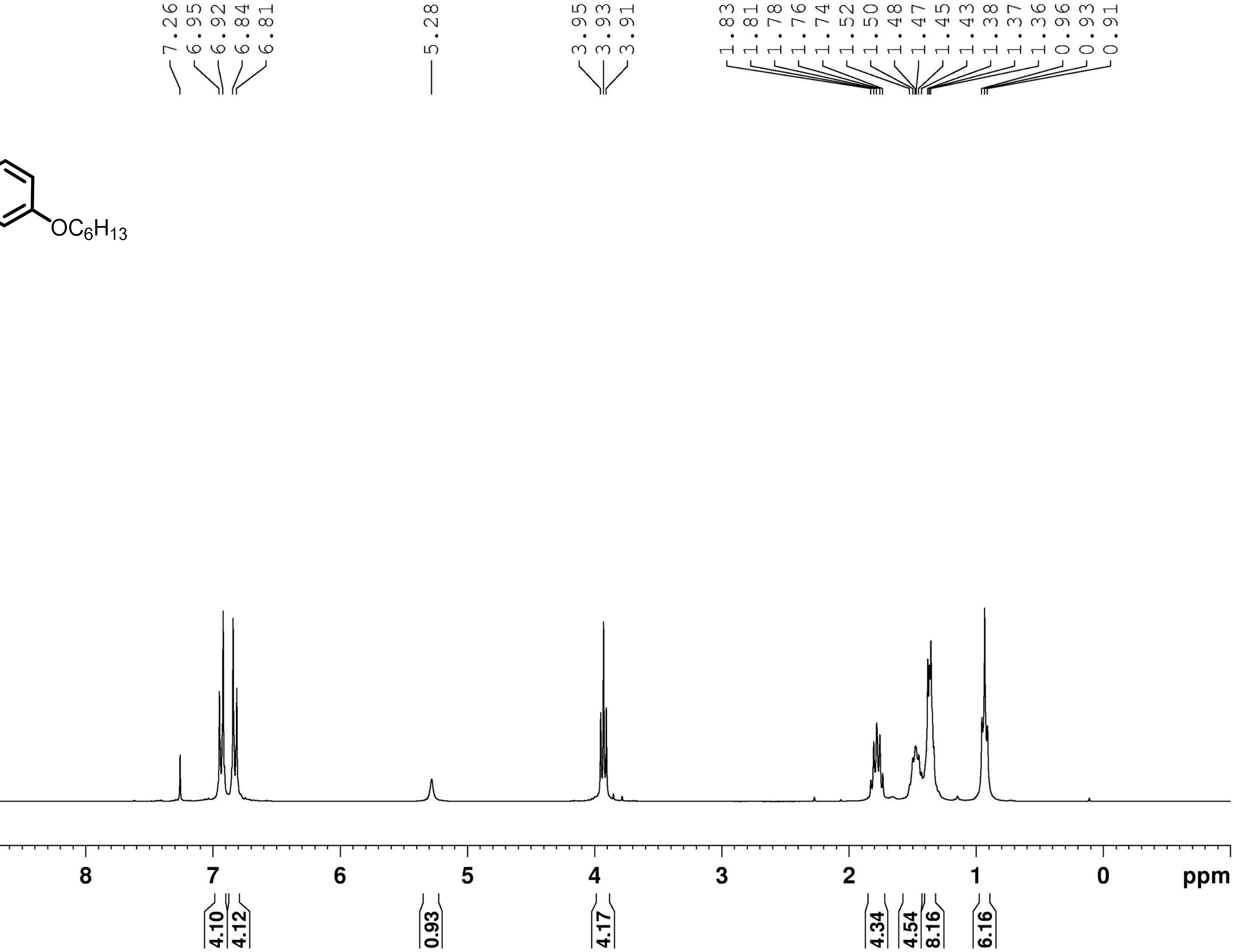

^1H NMR of compound **3b** (300 MHz, $CDCl_3$)

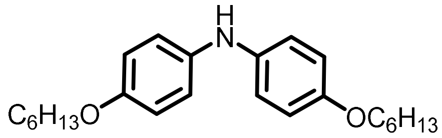

3b

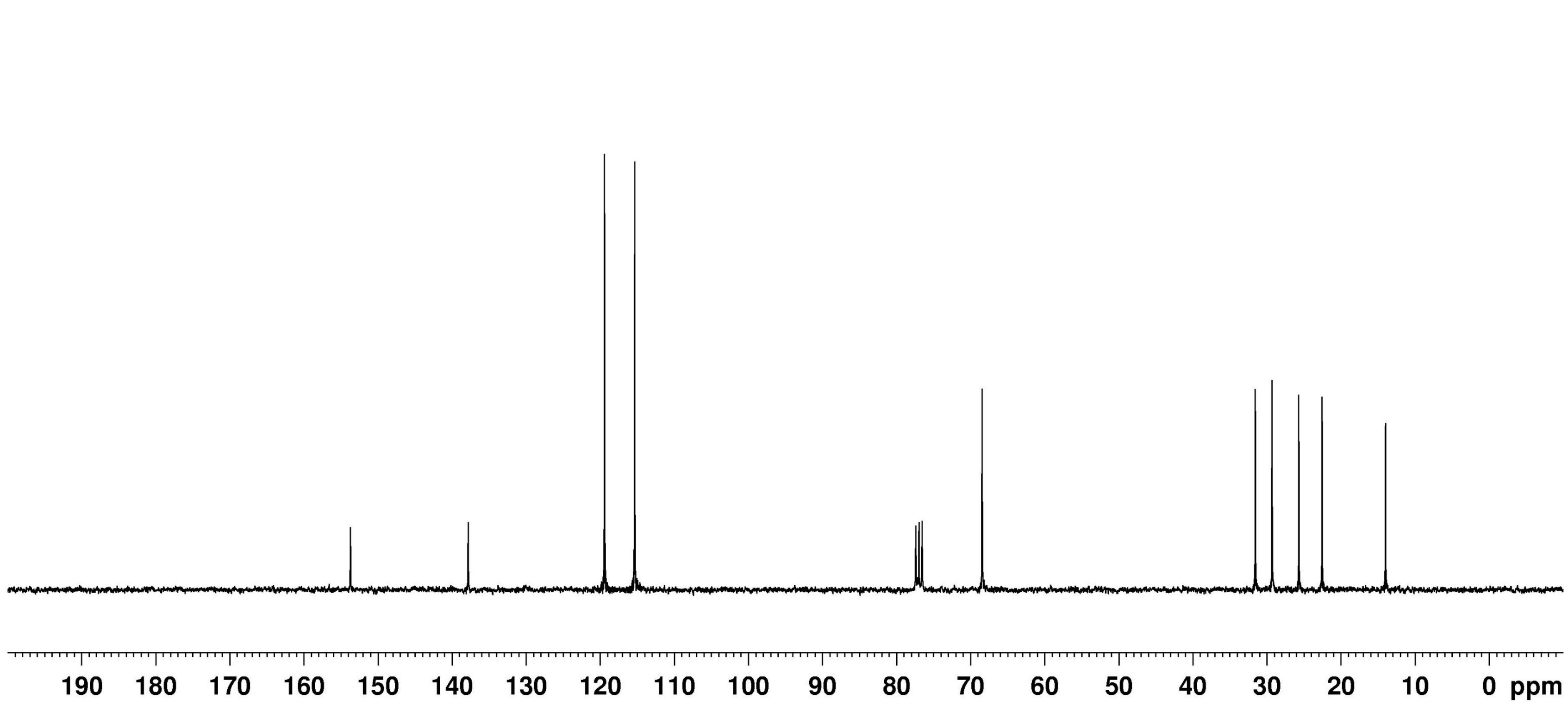

$^{13}C\{^1H\}$ NMR of compound **3b** (75 MHz, $CDCl_3$)

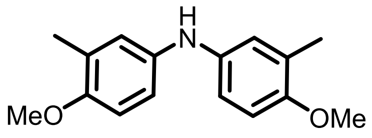

3c

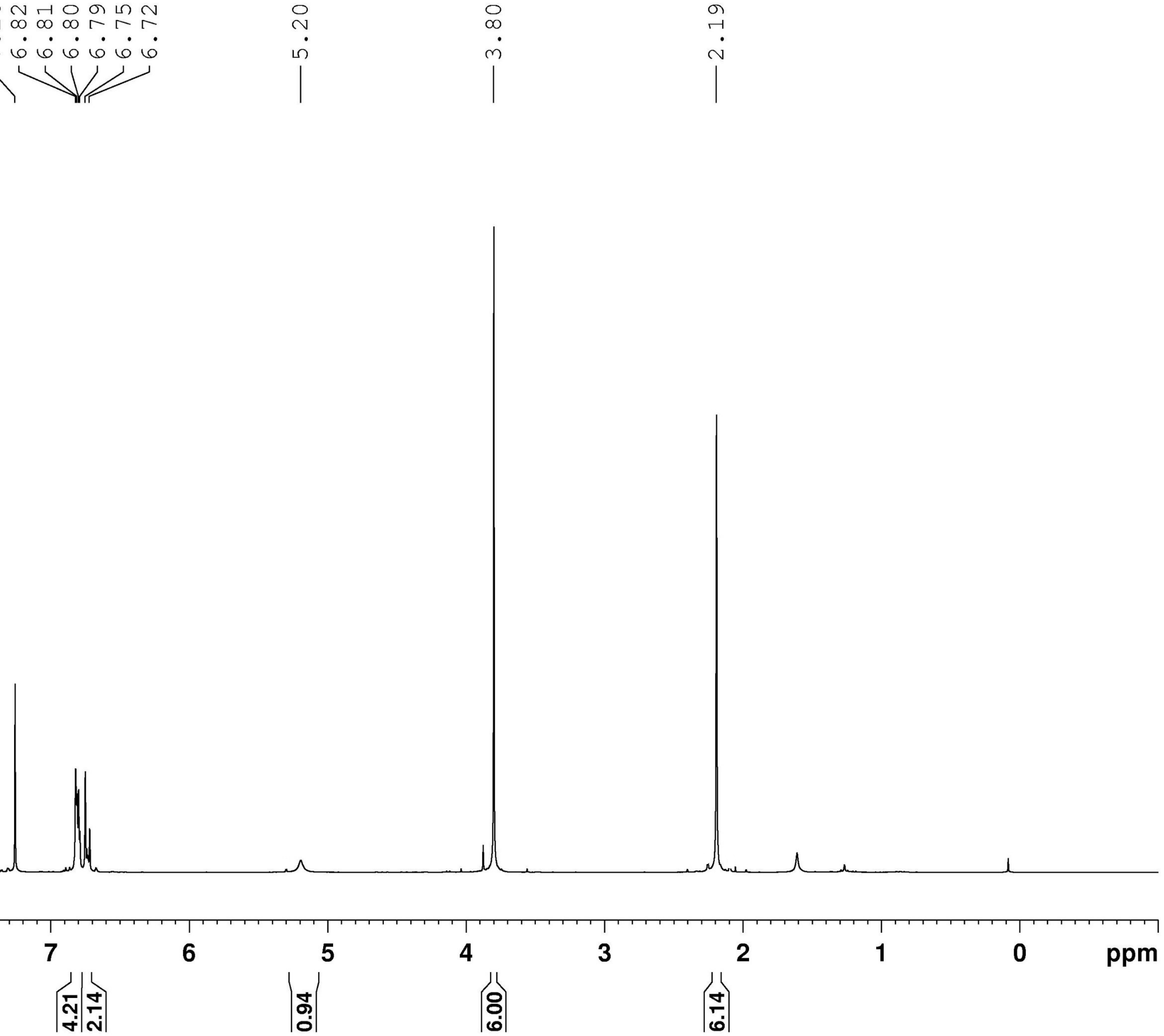

^{1}H NMR of compound **3c** (300 MHz, $CDCl_3$)

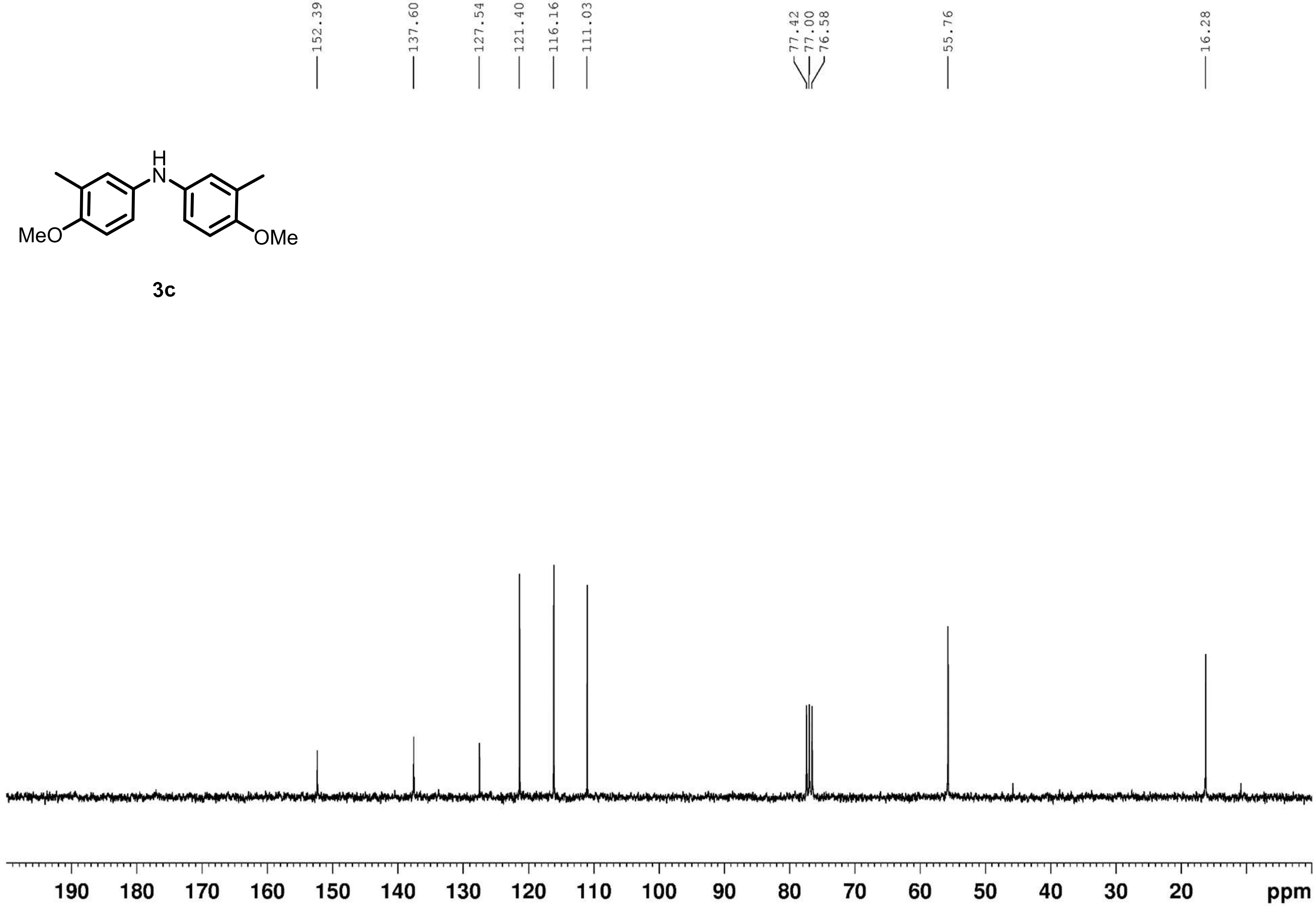

$^{13}C\{^{1}H\}$ NMR of compound **3c** (75 MHz, $CDCl_3$)

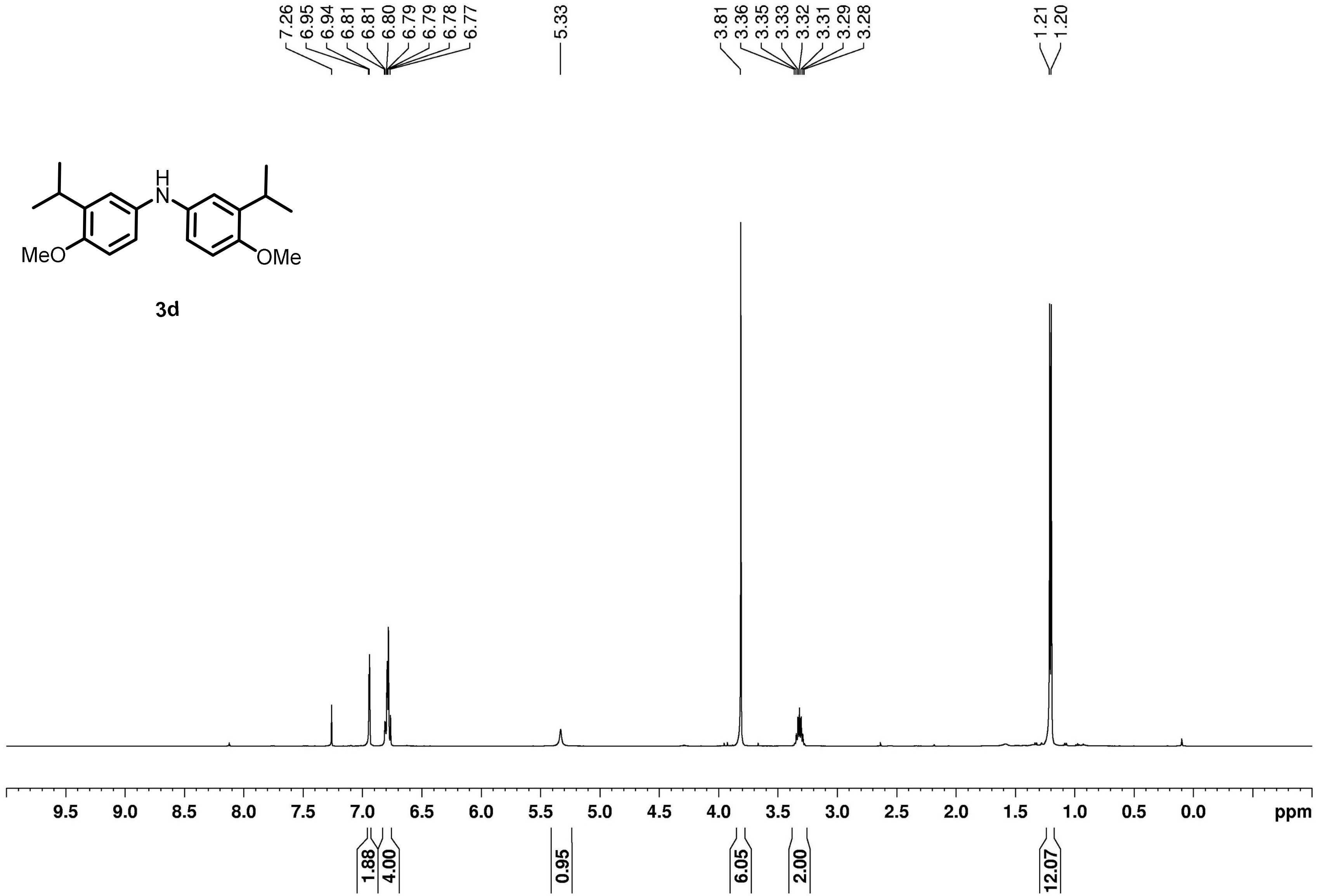

^{1}H NMR of compound **3d** (500 MHz, $CDCl_3$)

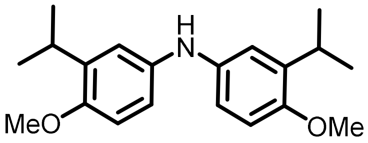

3d

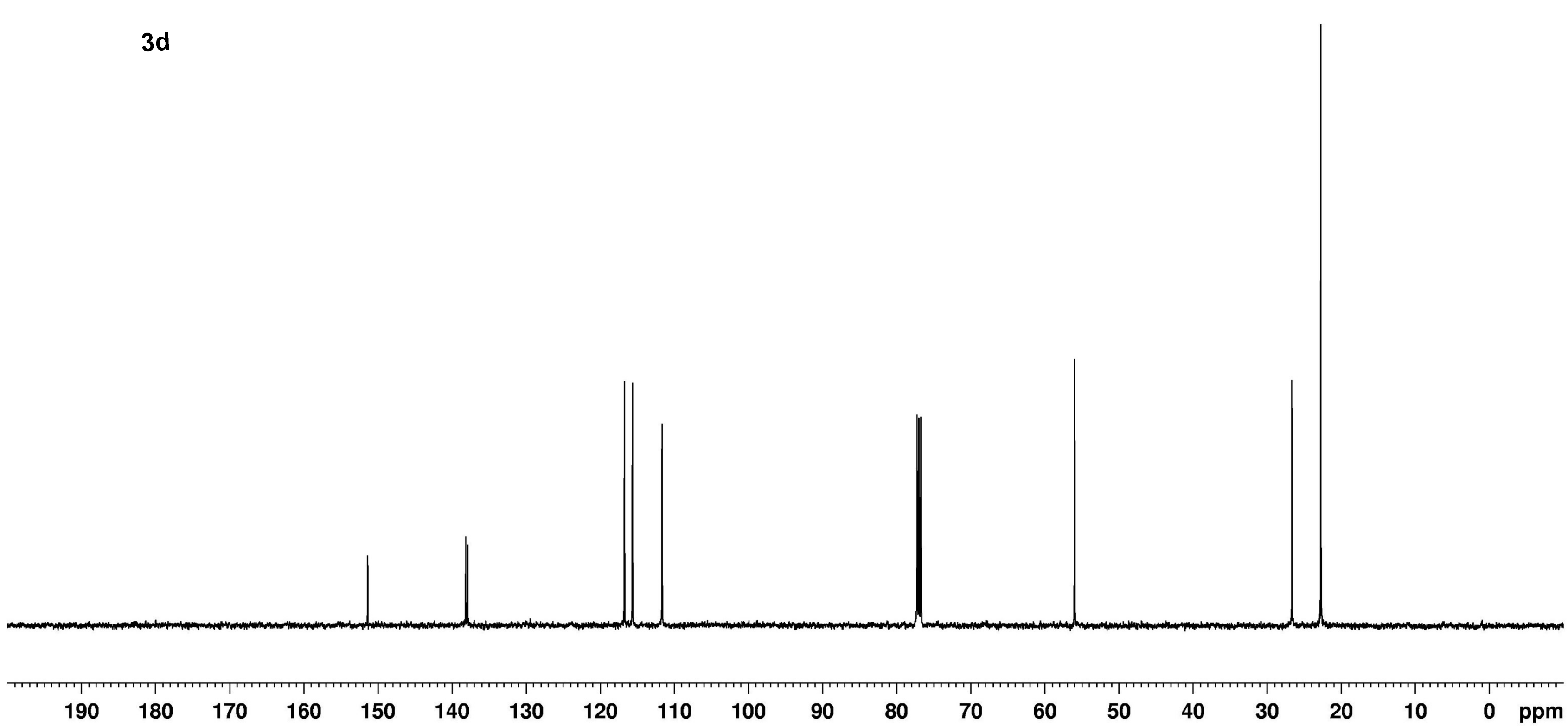

$^{13}C\{^{1}H\}$ NMR of compound **3d** (126 MHz, $CDCl_3$)

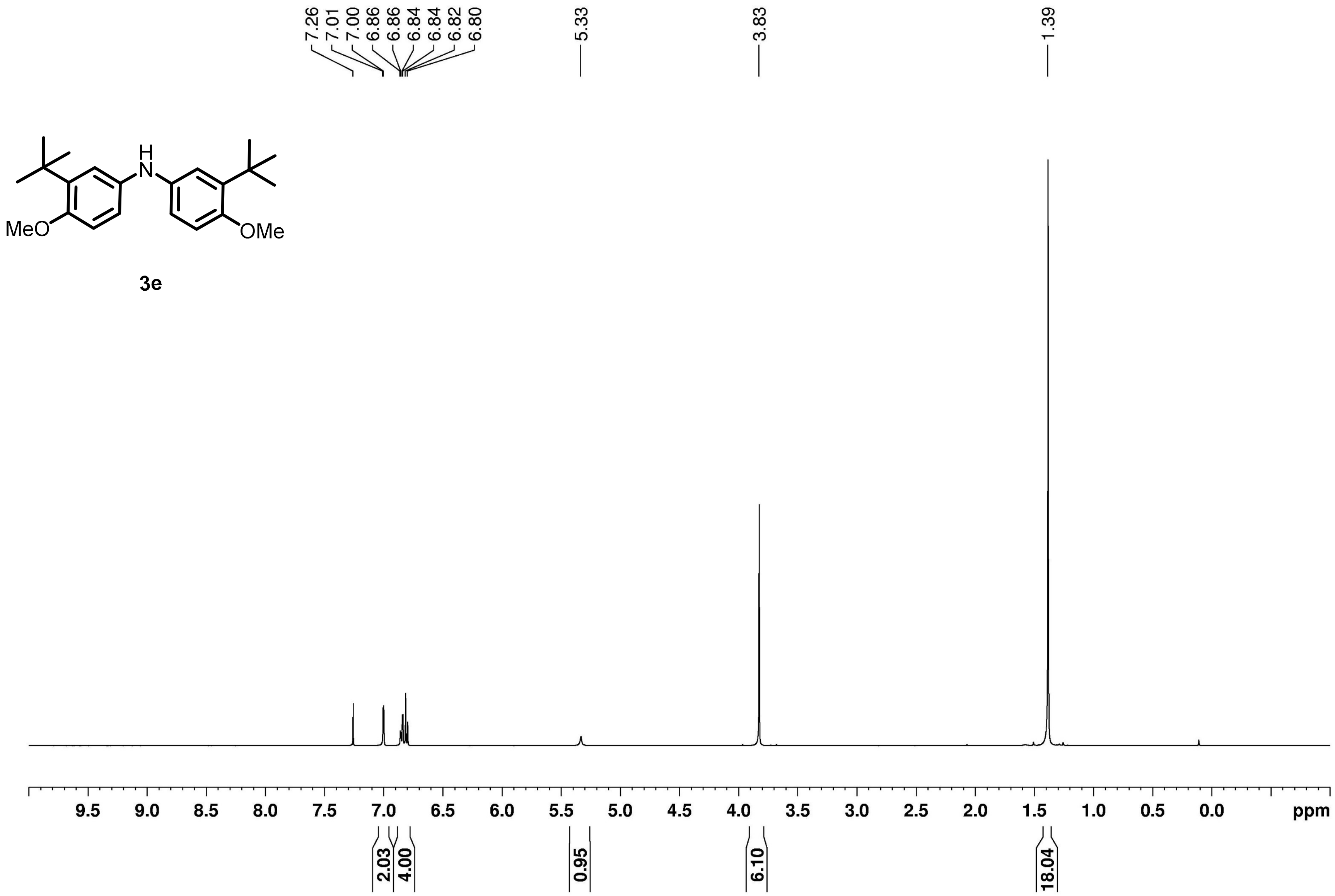

^{1}H NMR of compound **3e** (500 MHz, $CDCl_3$)

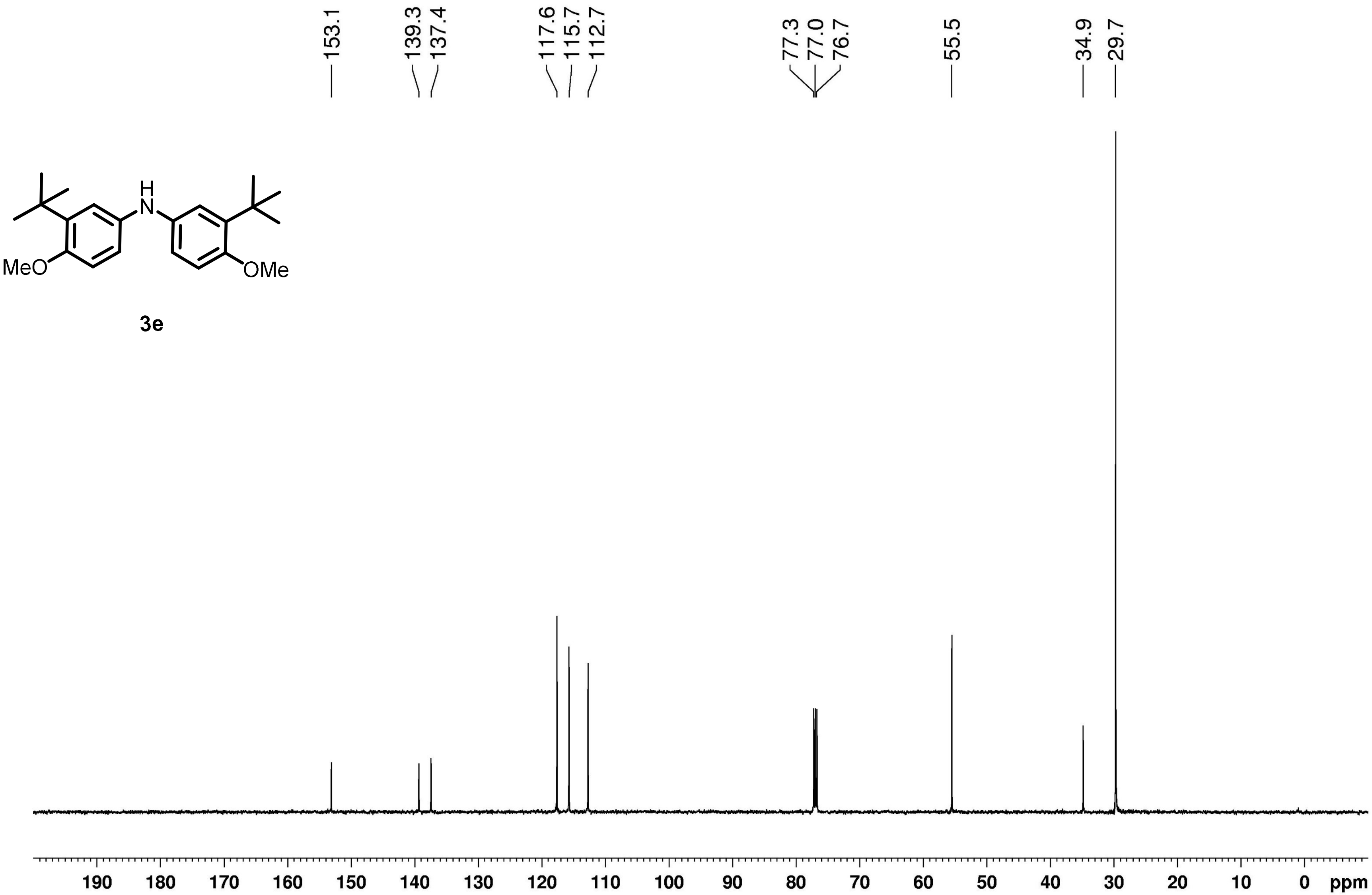

$^{13}C\{^{1}H\}$ NMR of compound **3e** (126 MHz, $CDCl_3$)

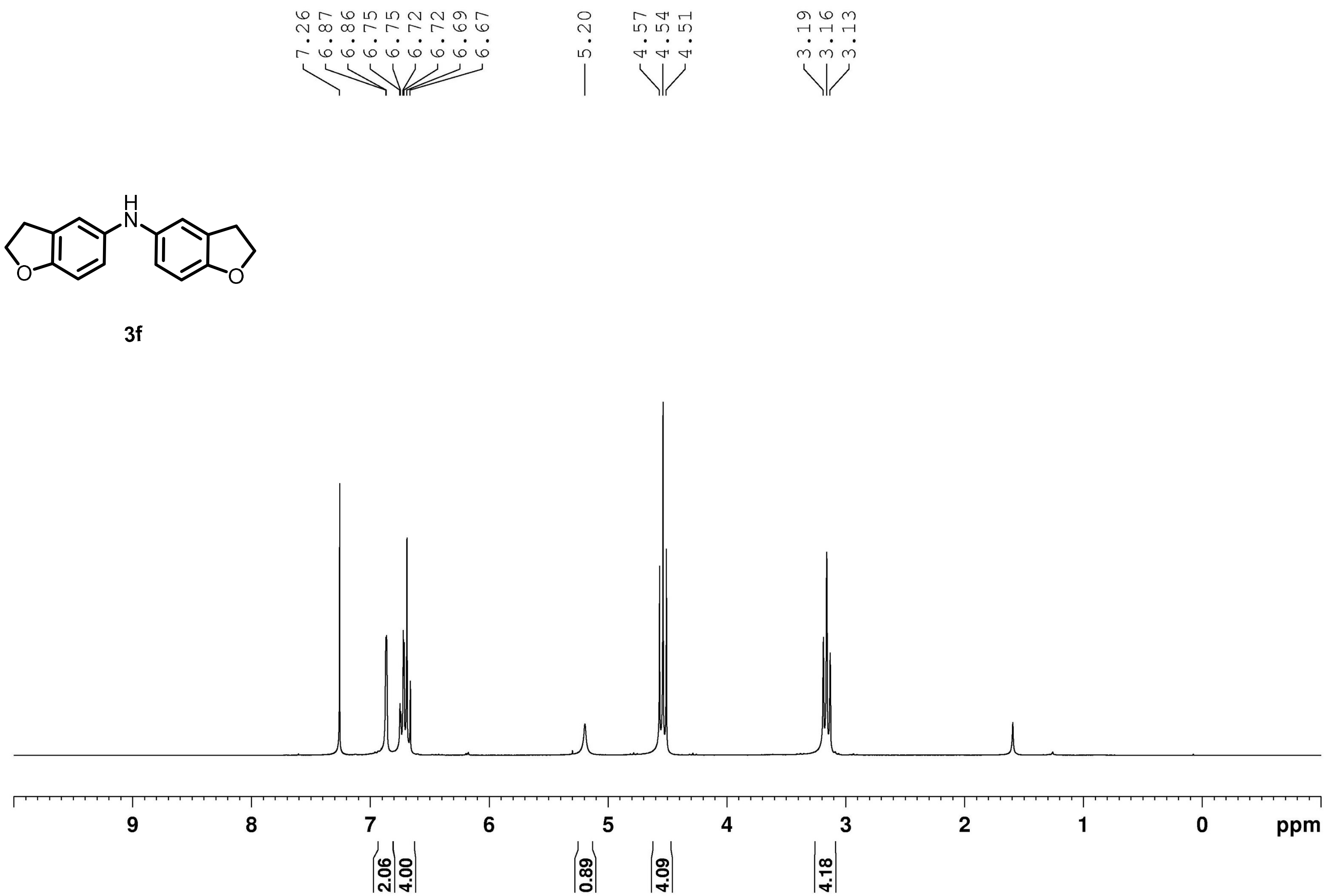

^{1}H NMR of compound **3f** (300 MHz, $CDCl_3$)

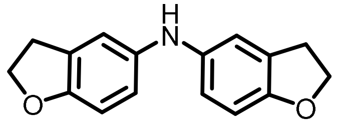

3f

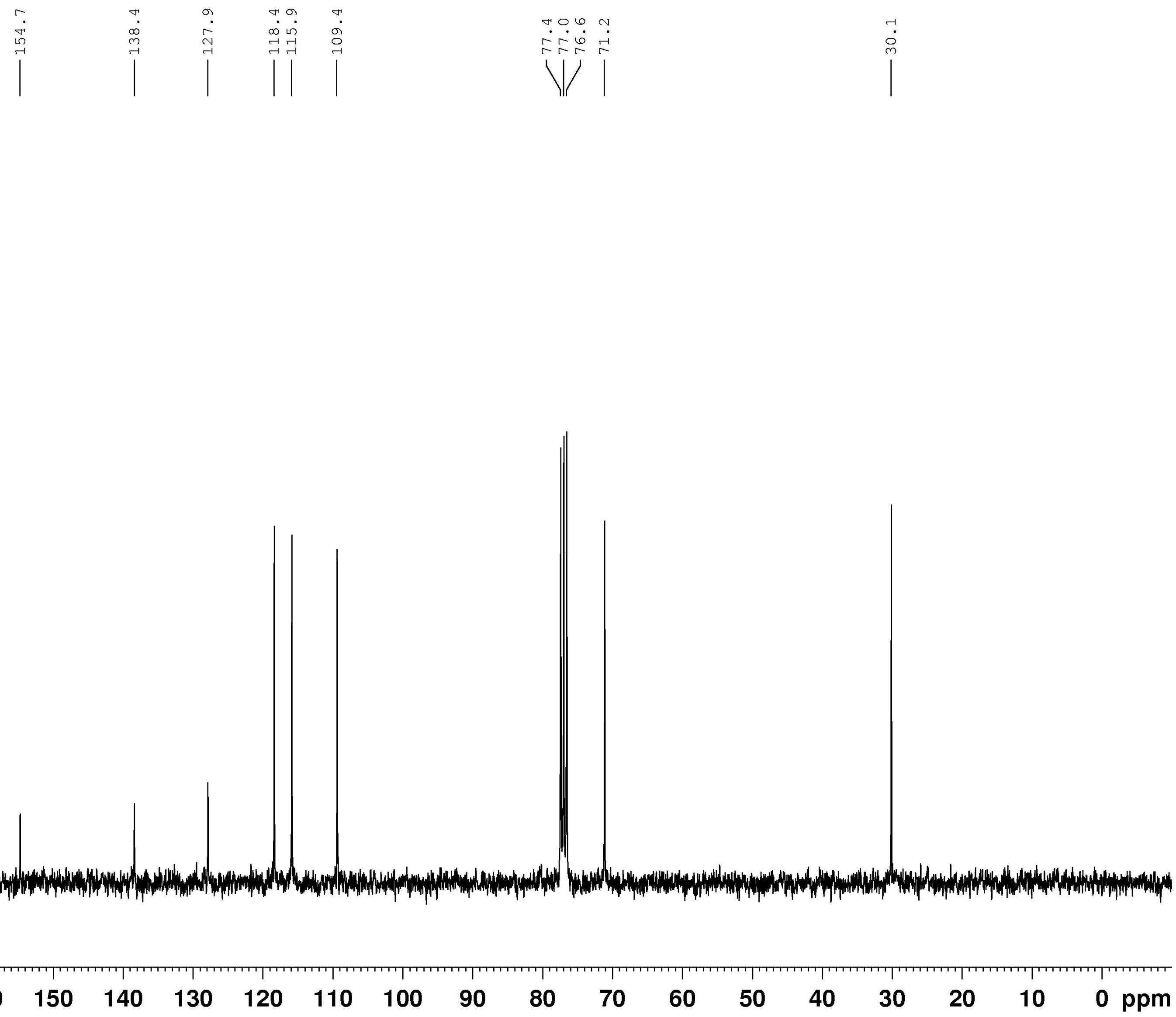

$^{13}C\{^{1}H\}$ NMR of compound **3f** (75 MHz, $CDCl_3$)

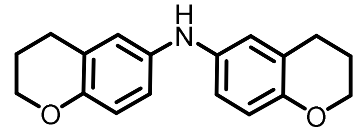

3g

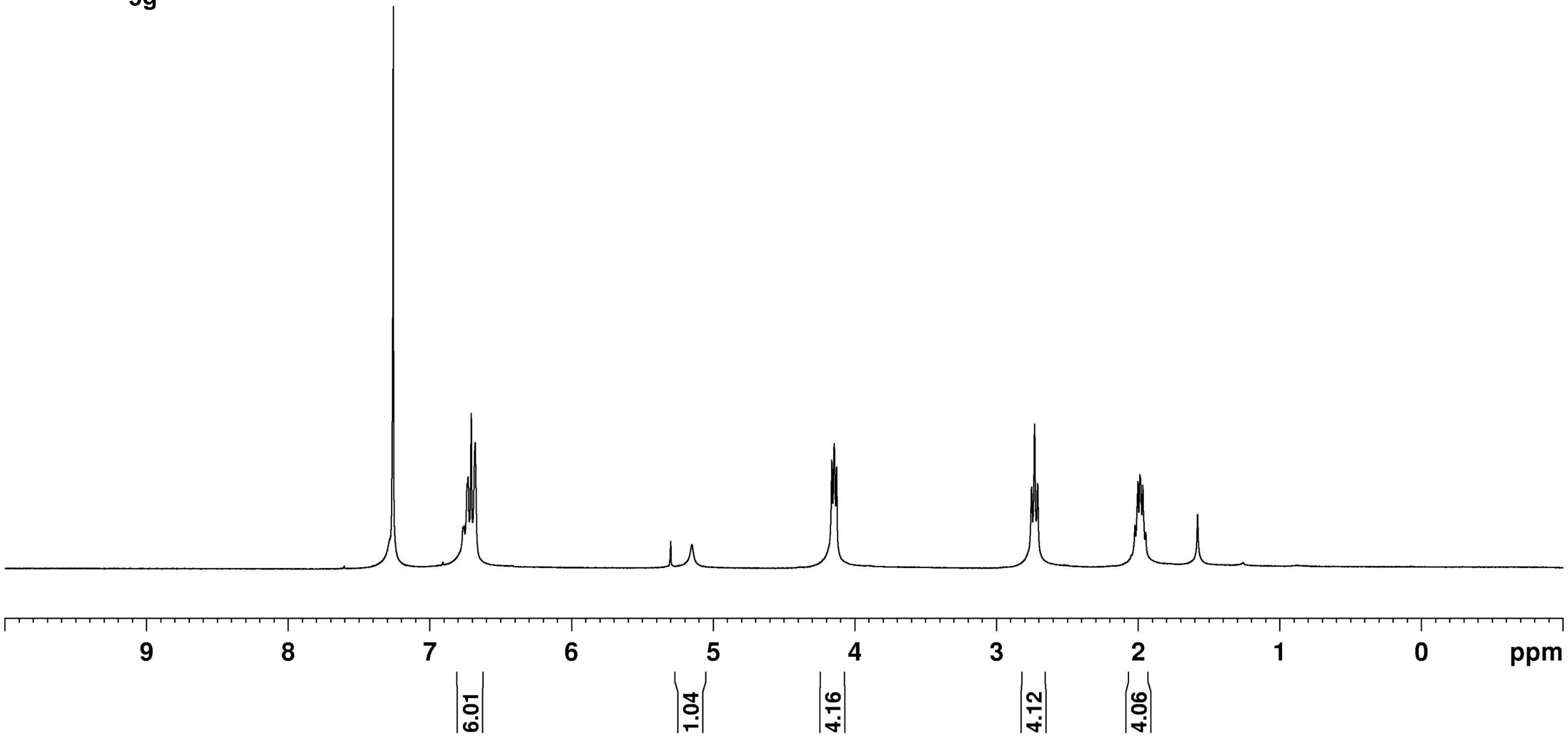

^{1}H NMR of compound **3g** (300 MHz, $CDCl_3$)

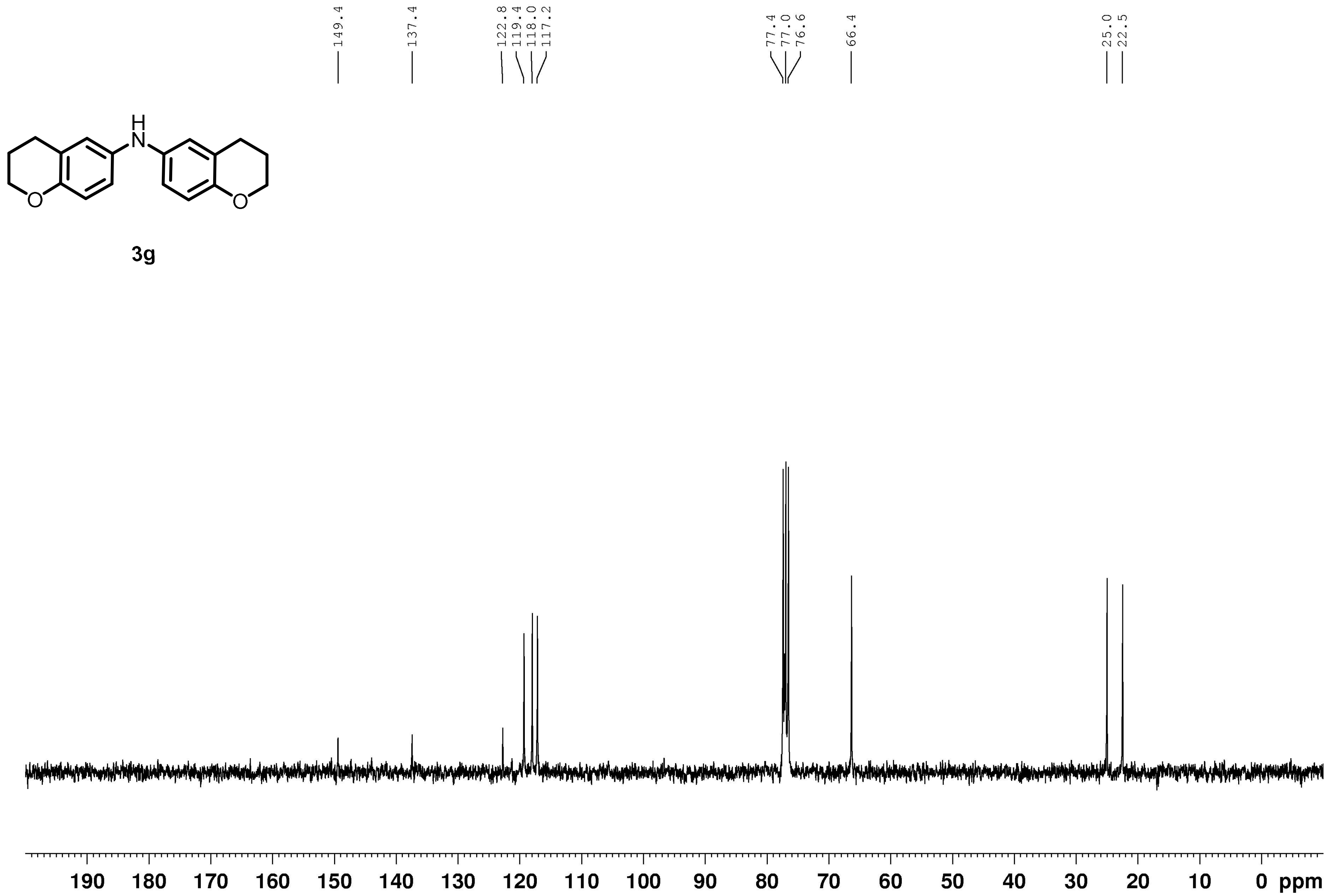

$^{13}C\{^{1}H\}$ NMR of compound **3g** (75 MHz, $CDCl_3$)

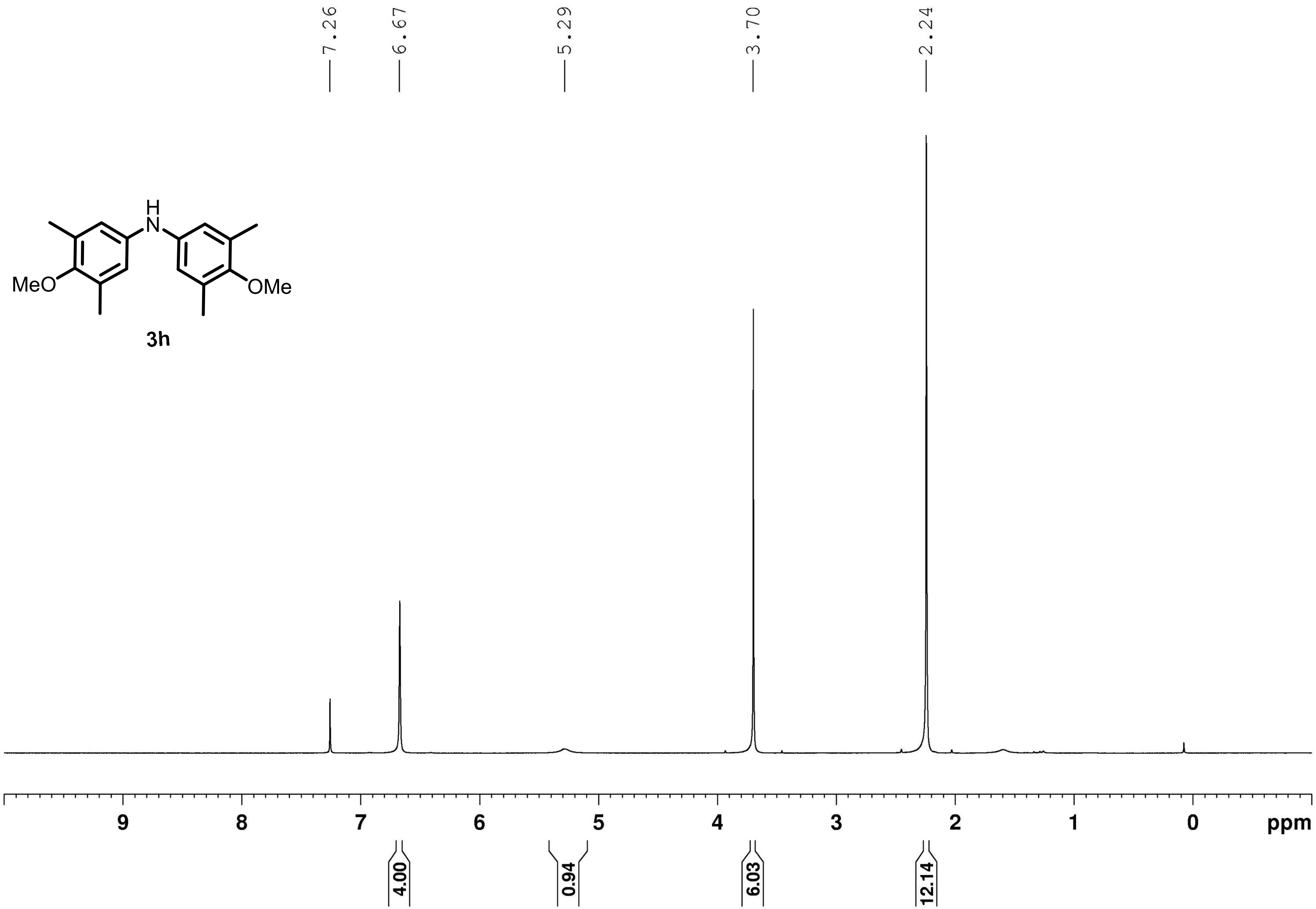

^{1}H NMR of compound **3h** (300 MHz, $CDCl_3$)

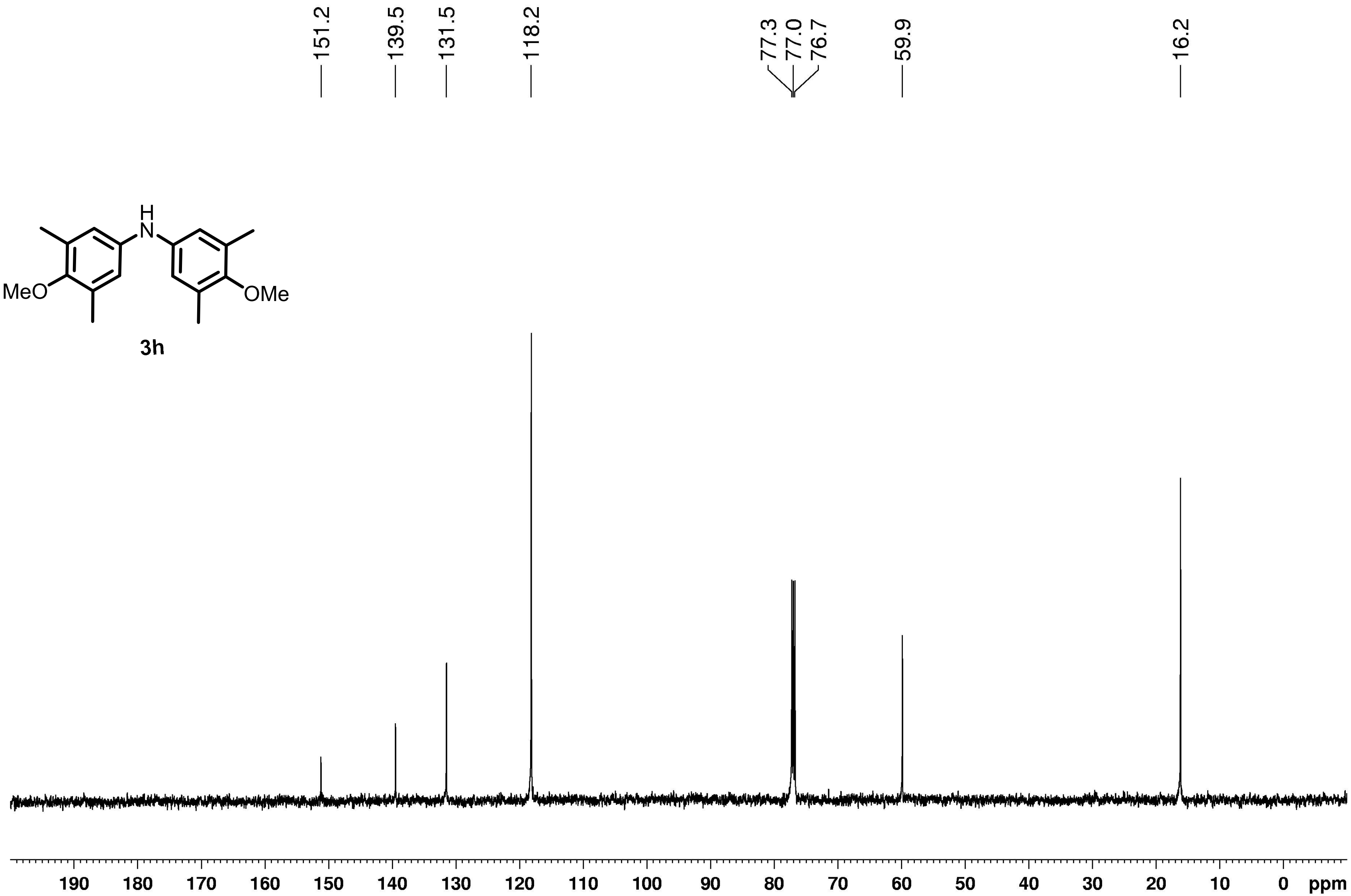

$^{13}C\{^{1}H\}$ NMR of compound **3h** (126 MHz, $CDCl_3$)

SS3231

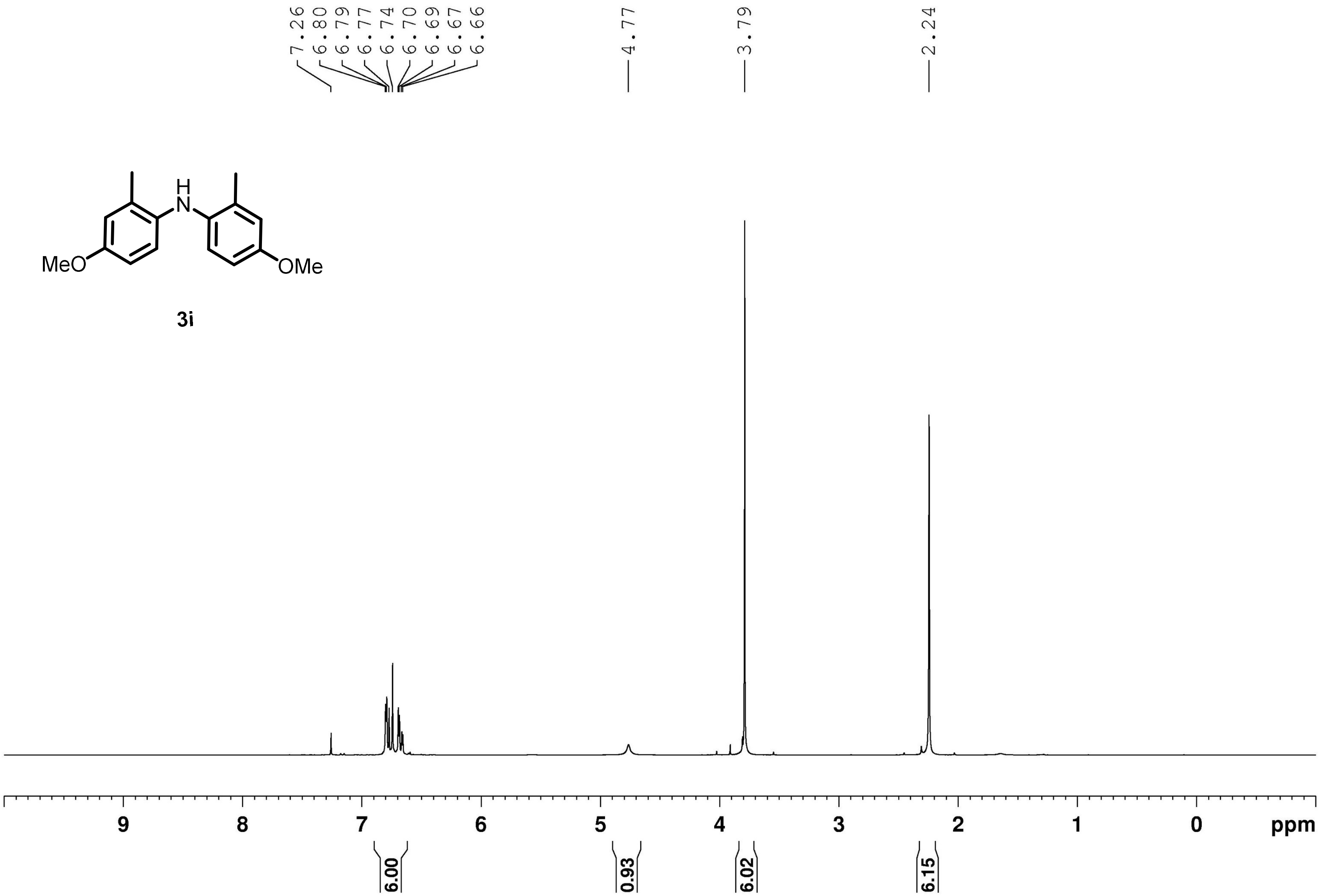

^{1}H NMR of compound **3i** (300 MHz, $CDCl_3$)

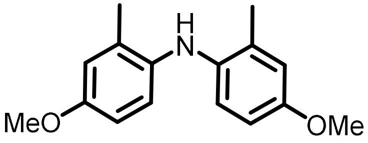

3i

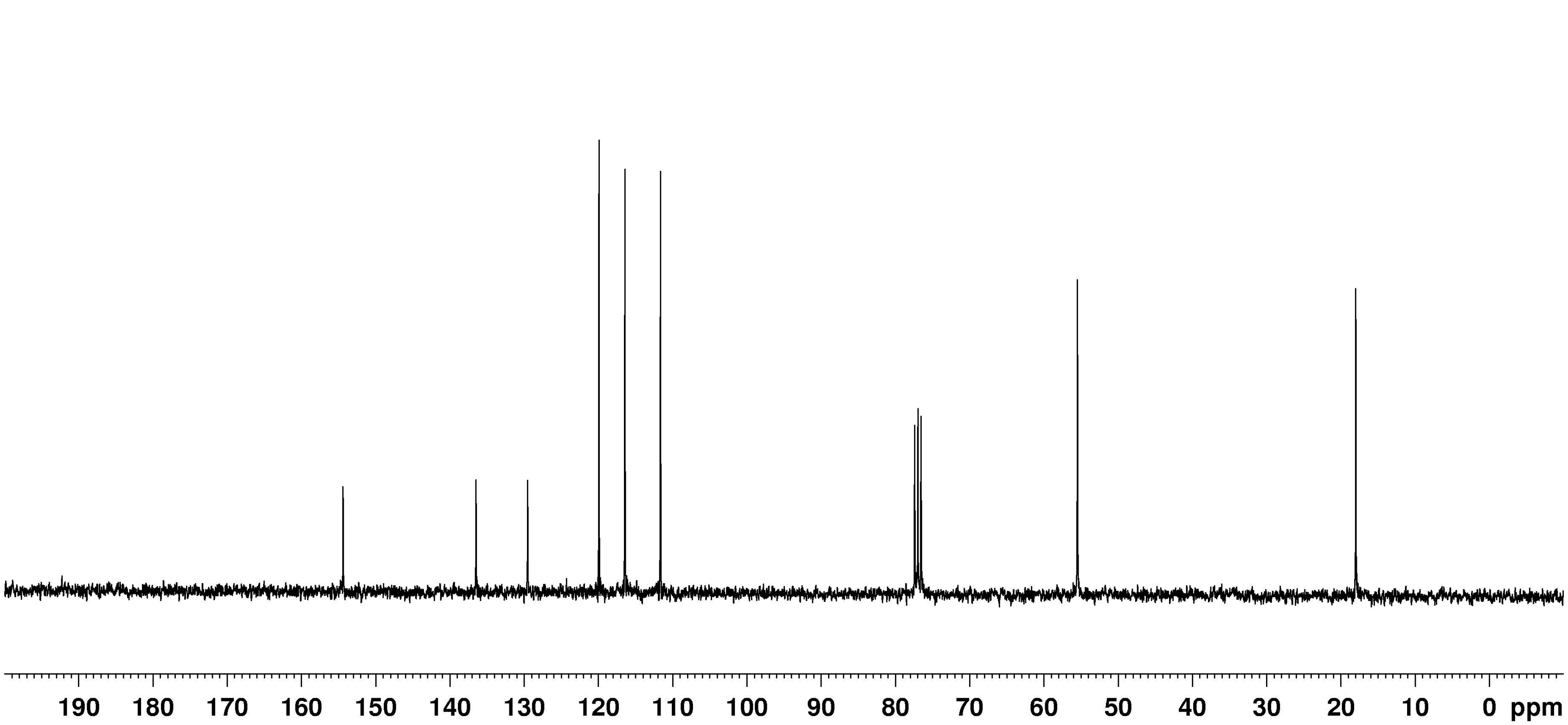

$^{13}C\{^{1}H\}$ NMR of compound **3i** (75 MHz, $CDCl_3$)

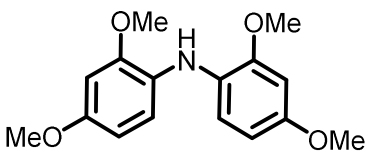

3j

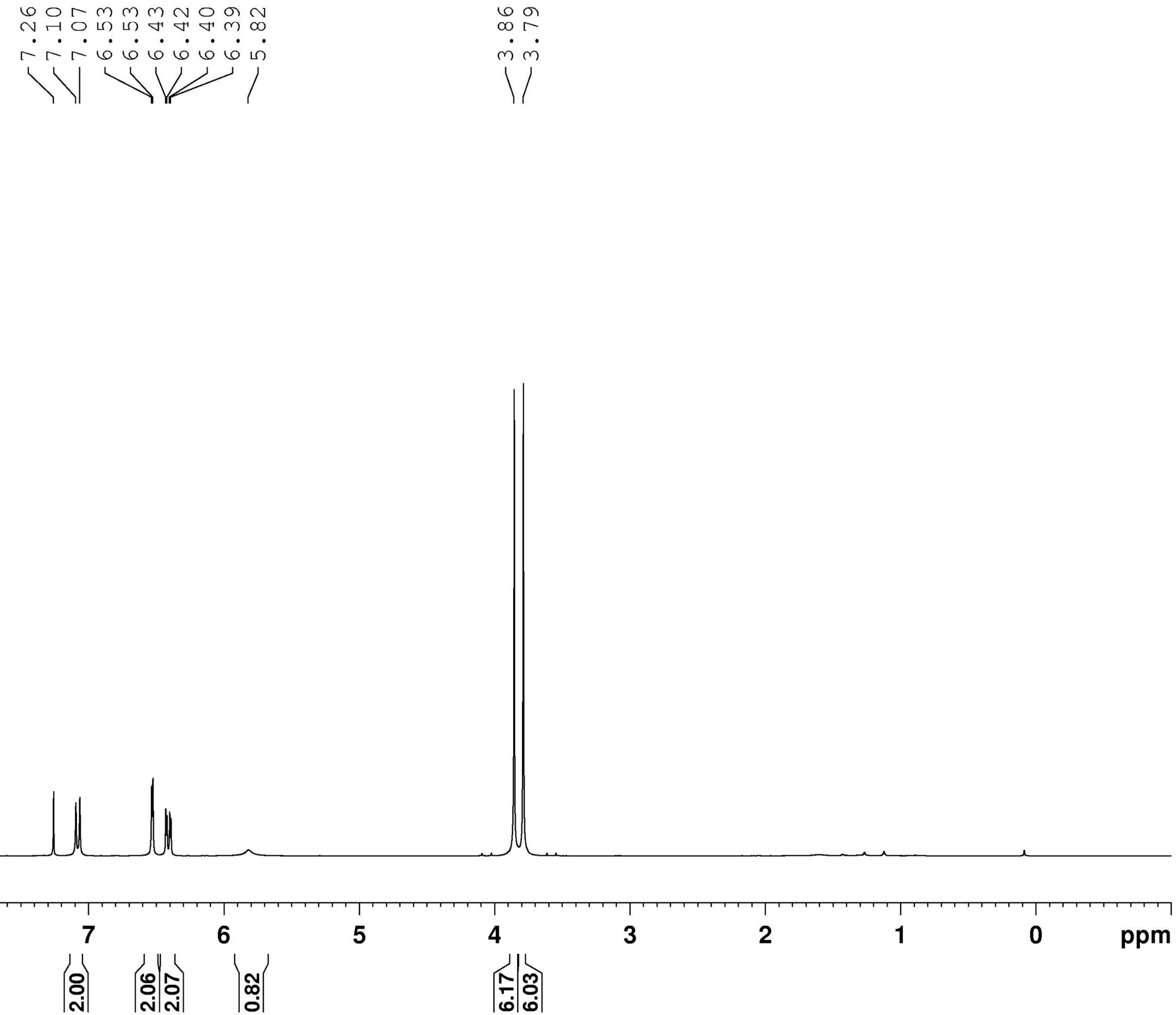

^{1}H NMR of compound **3j** (300 MHz, $CDCl_3$)

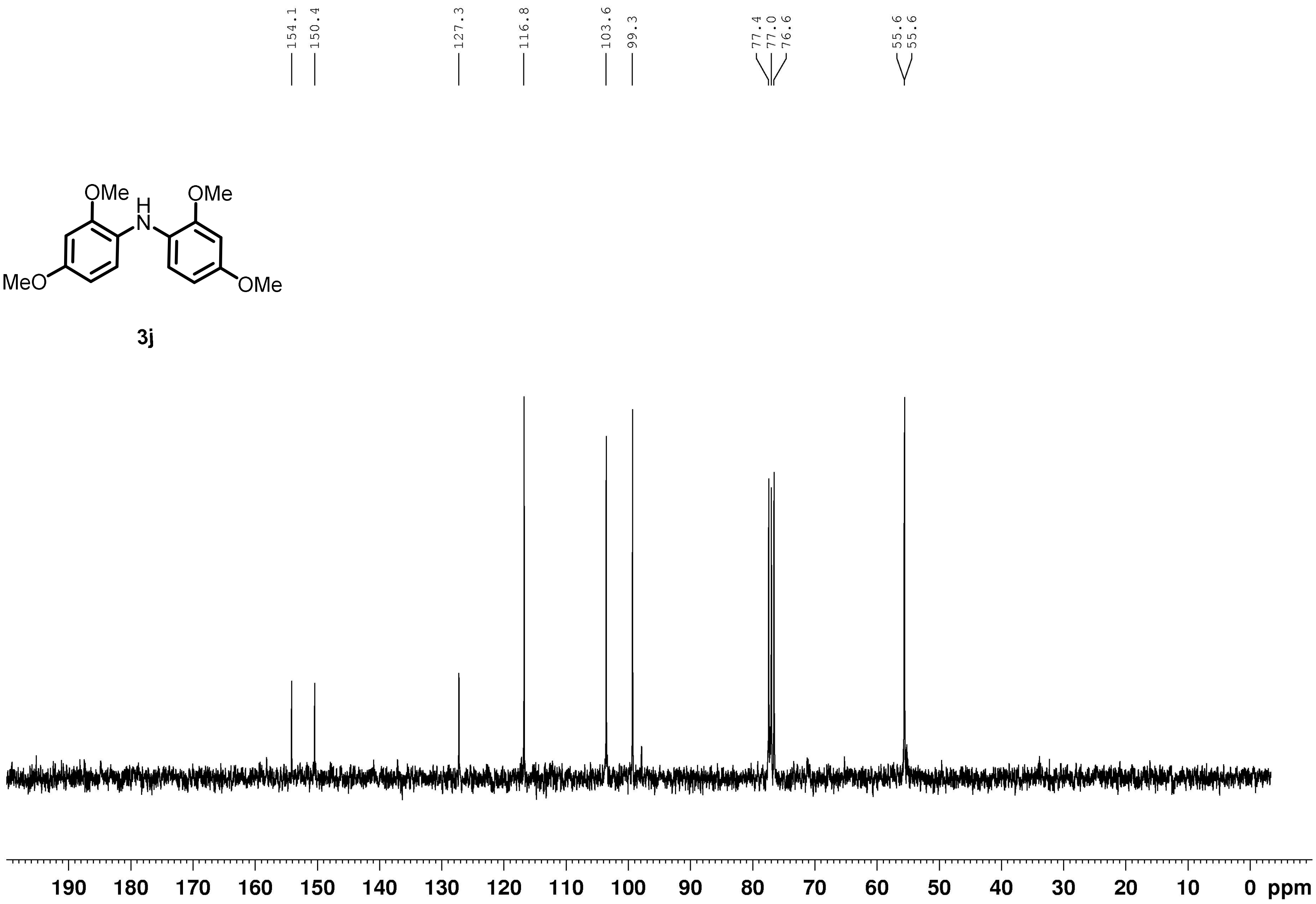

$^{13}C\{^{1}H\}$ NMR of compound **3j** (75 MHz, $CDCl_3$)

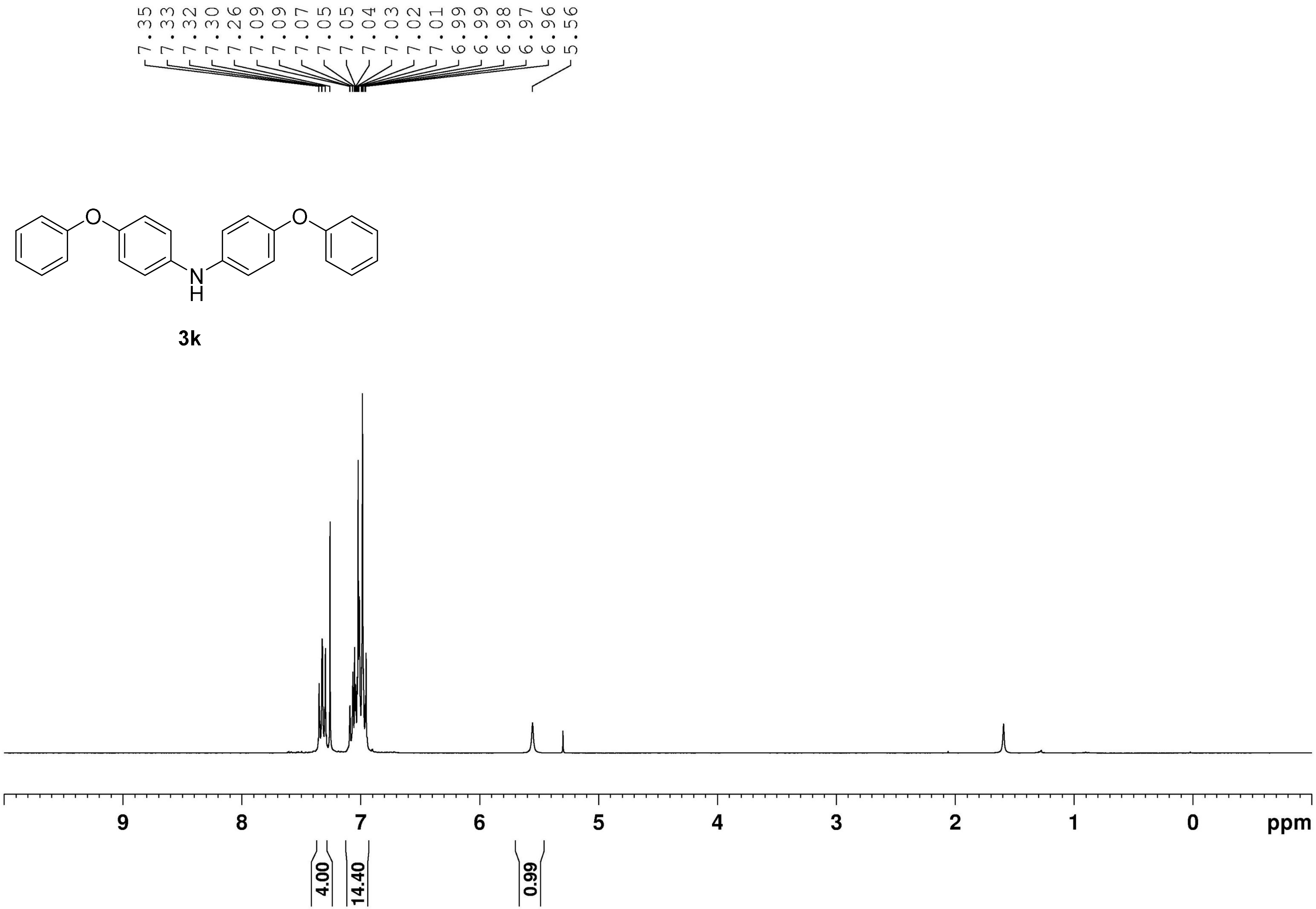

^{1}H NMR of compound **3k** (300 MHz, $CDCl_3$)

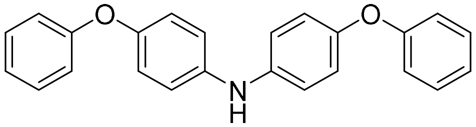

3k

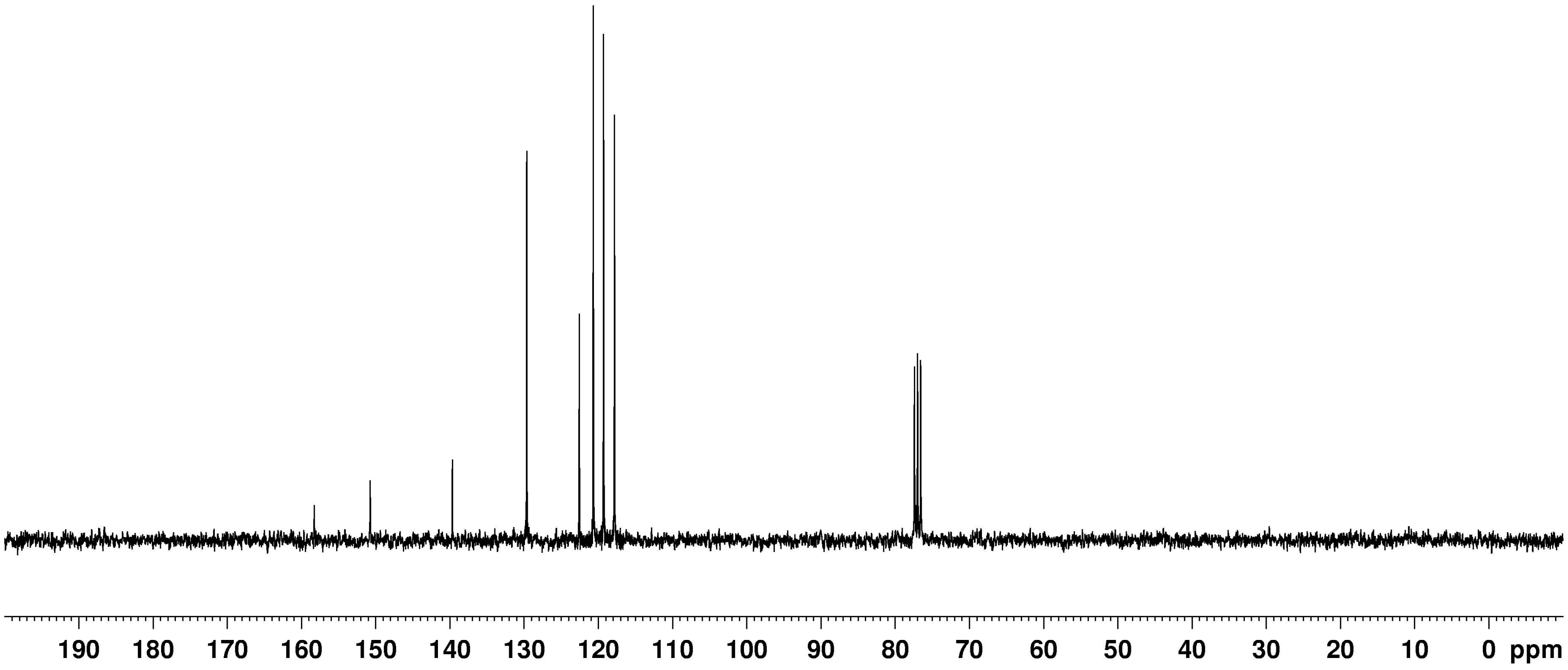

$^{13}C\{^{1}H\}$ NMR of compound **3k** (75 MHz, $CDCl_3$)

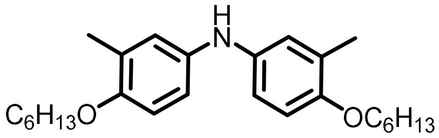

3l

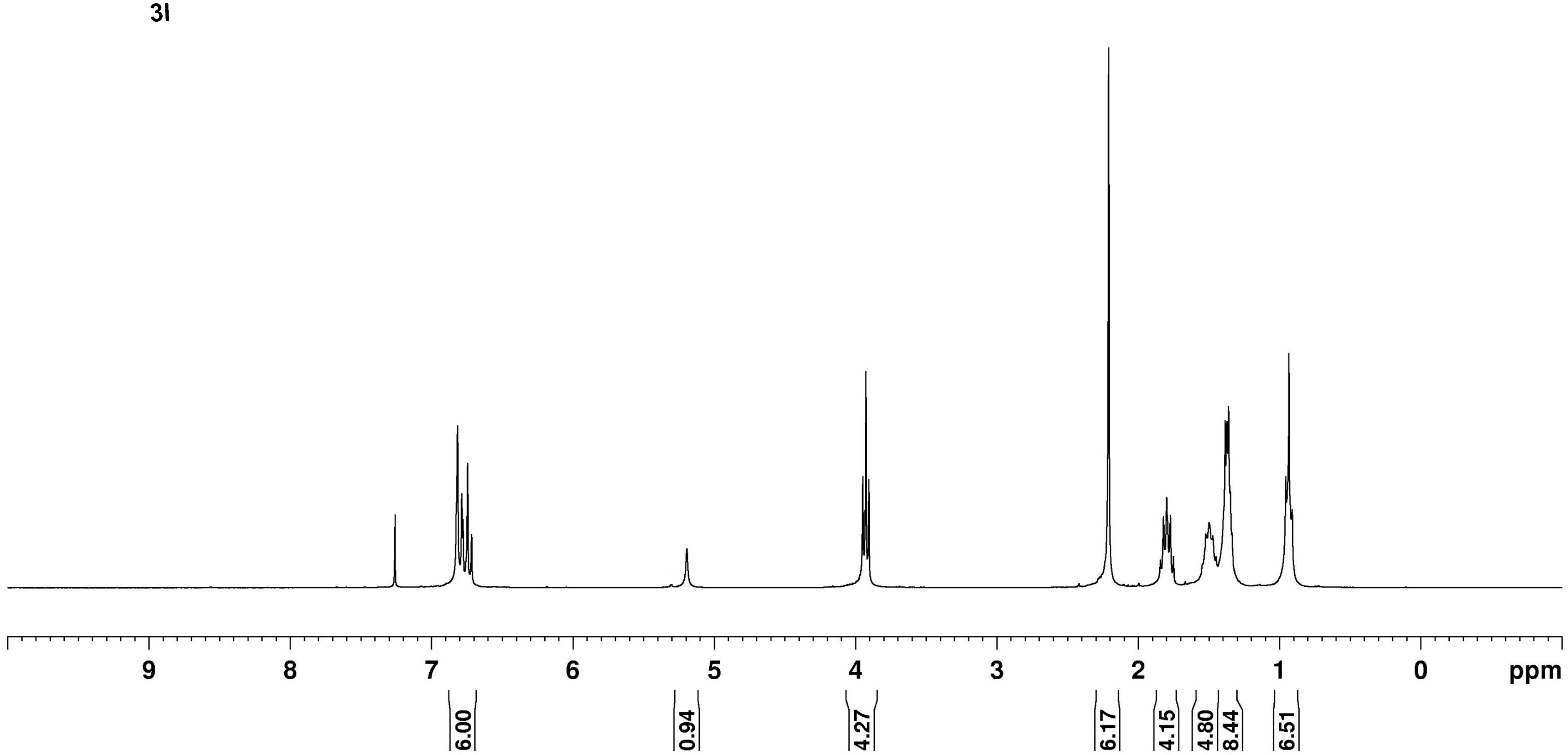

^{1}H NMR of compound **3l** (300 MHz, $CDCl_3$)

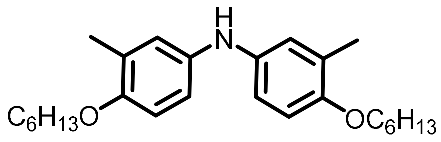

3l

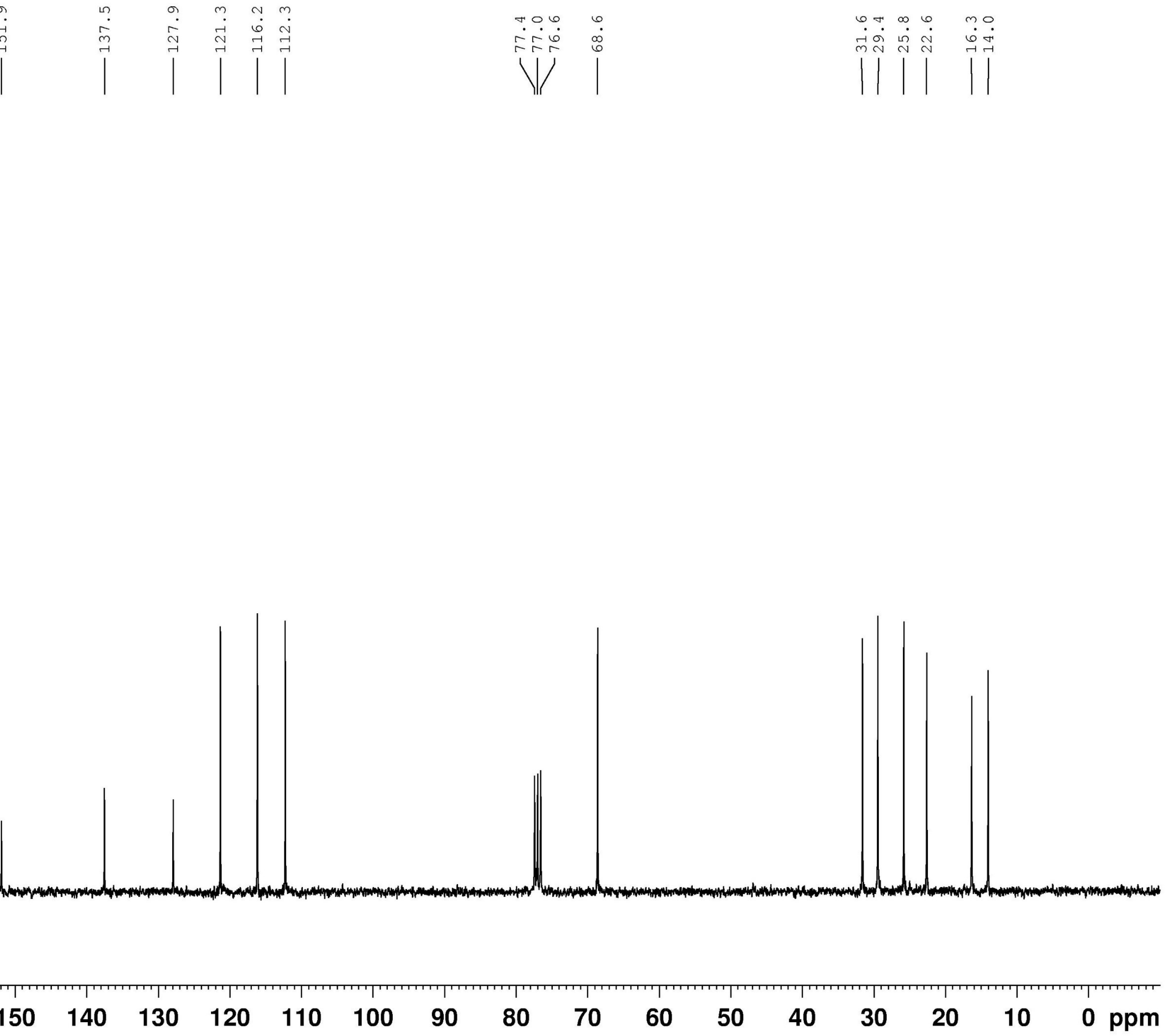

$^{13}C\{^{1}H\}$ NMR of compound **3l** (75 MHz, $CDCl_3$)

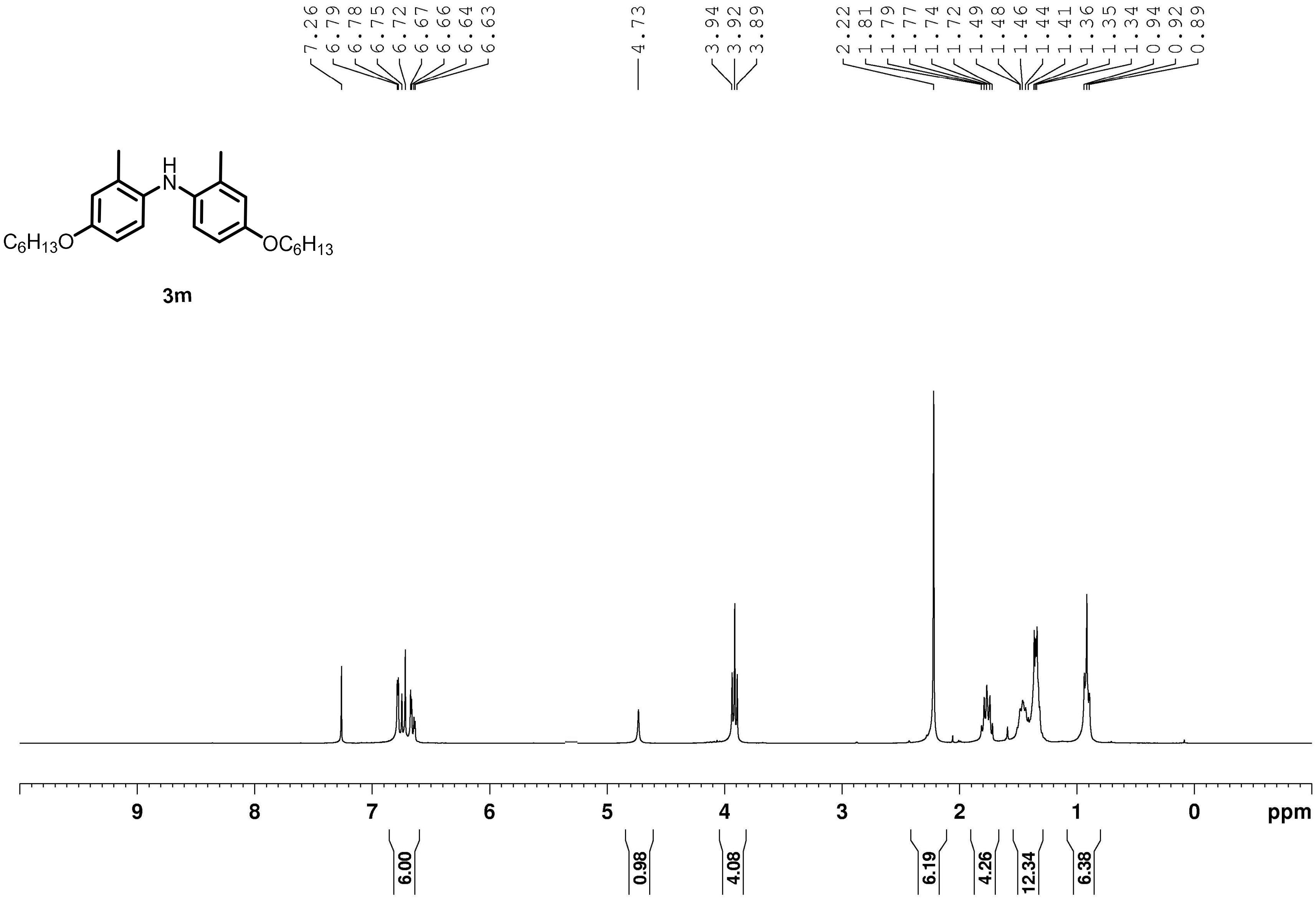

^{1}H NMR of compound **3m** (300 MHz, $CDCl_3$)

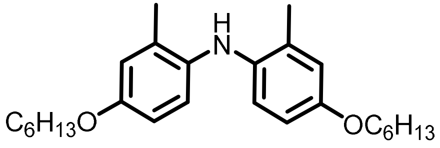

3m

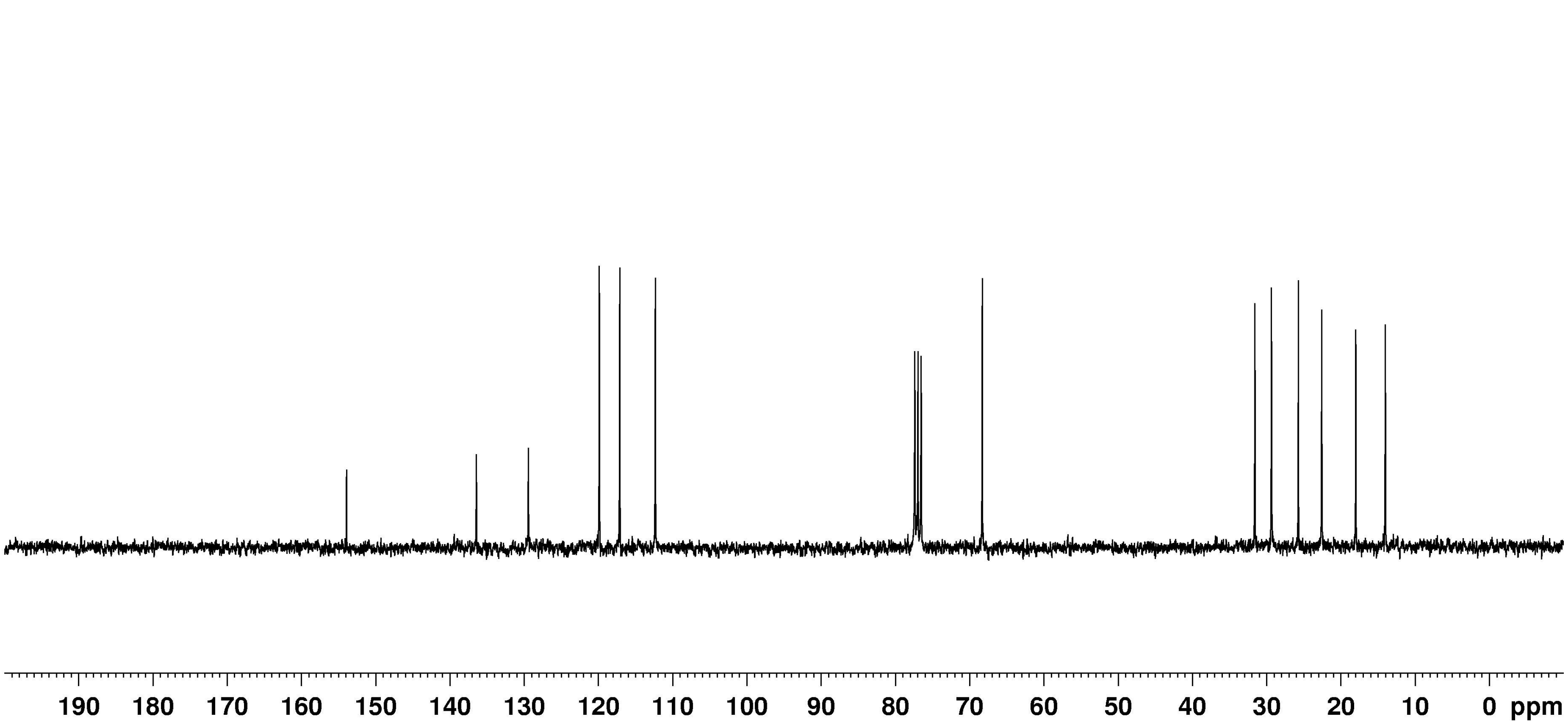

$^{13}C\{^{1}H\}$ NMR of compound **3m** (75 MHz, $CDCl_3$)

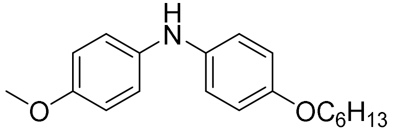

3ab

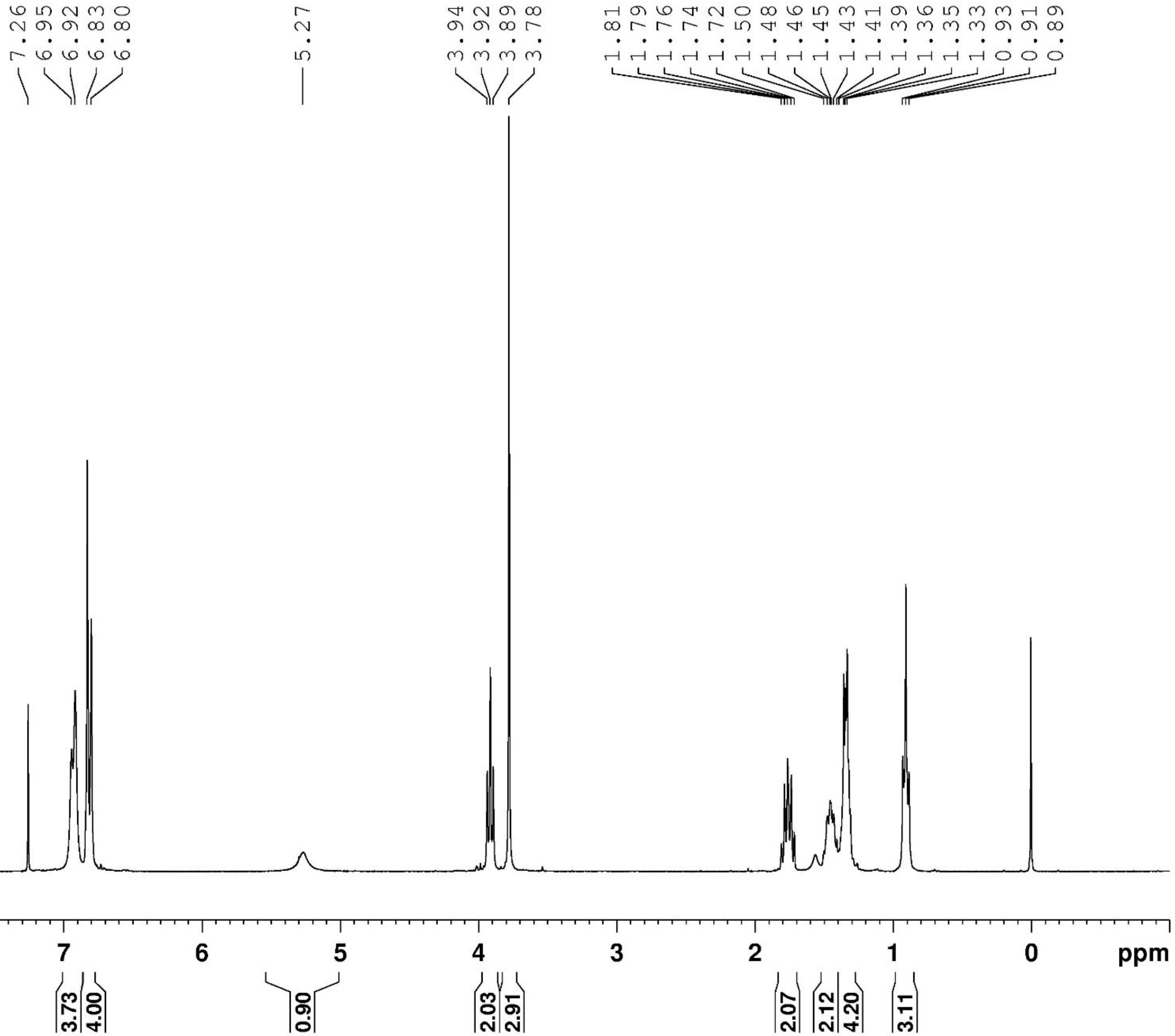

^{1}H NMR of compound **3ab** (300 MHz, $CDCl_3$)

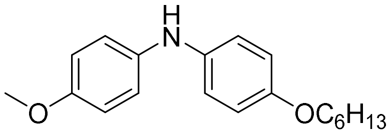

3ab

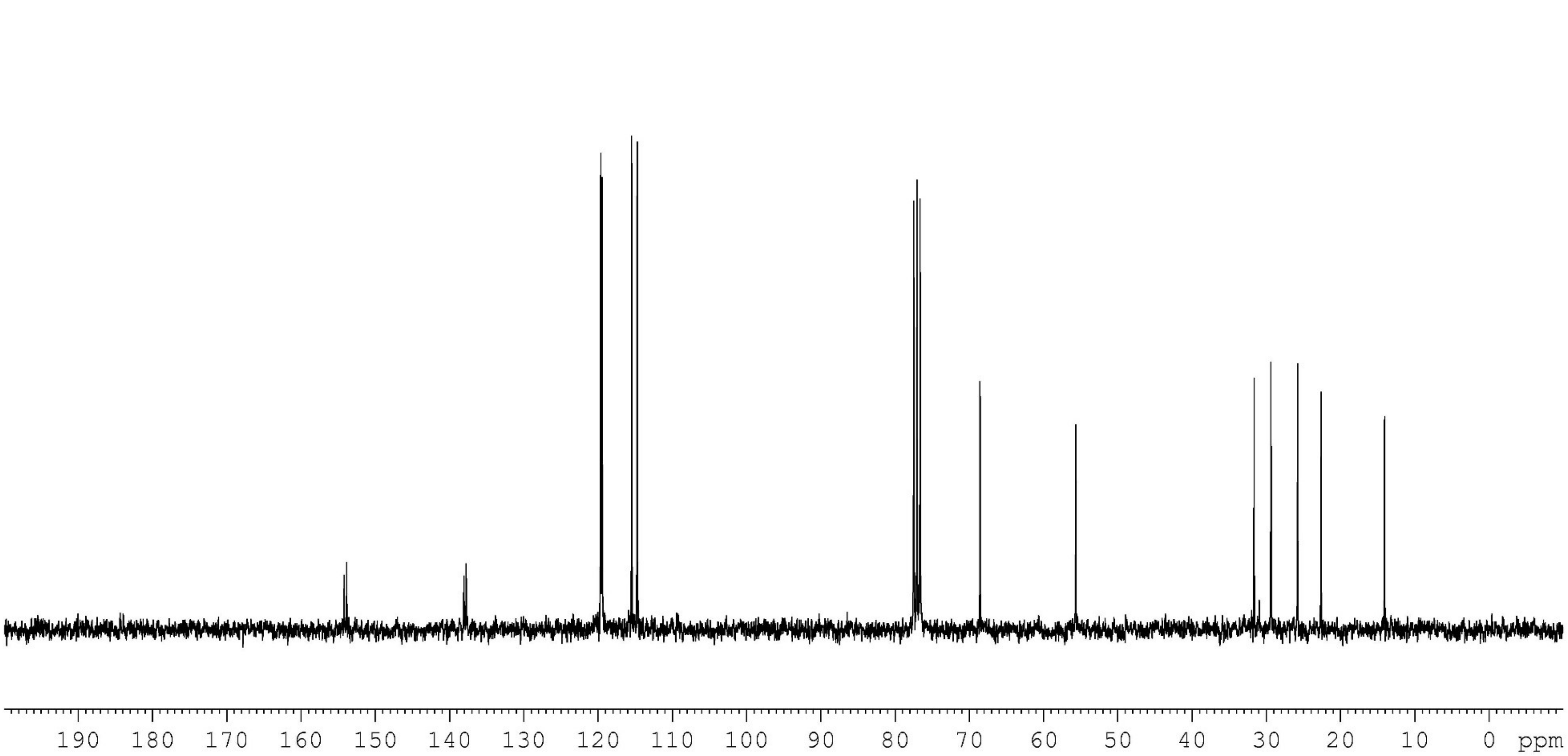

^{13}C{^{1}H} NMR of compound **3ab** (75 MHz, $CDCl_3$)

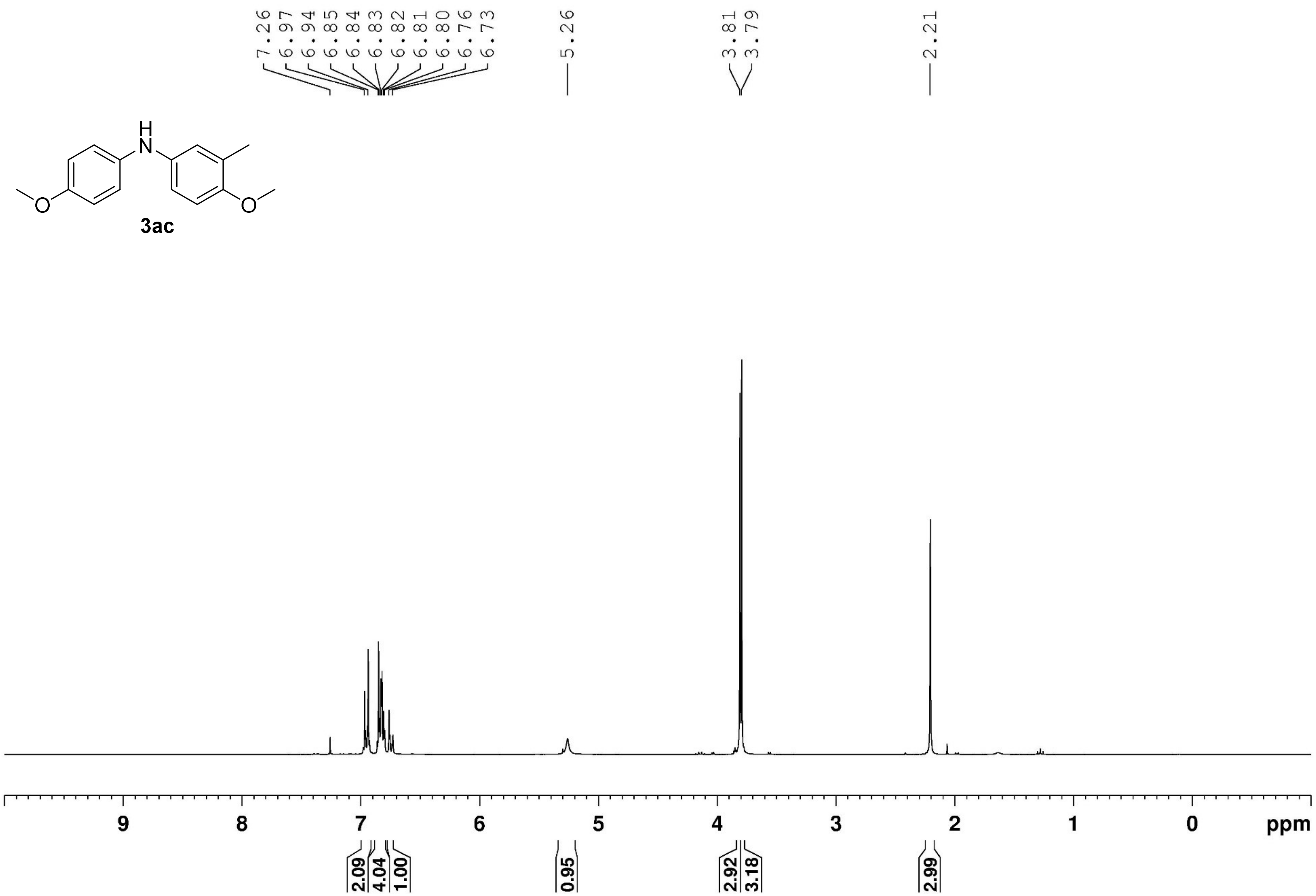

^{1}H NMR of compound **3ac** (300 MHz, $CDCl_3$)

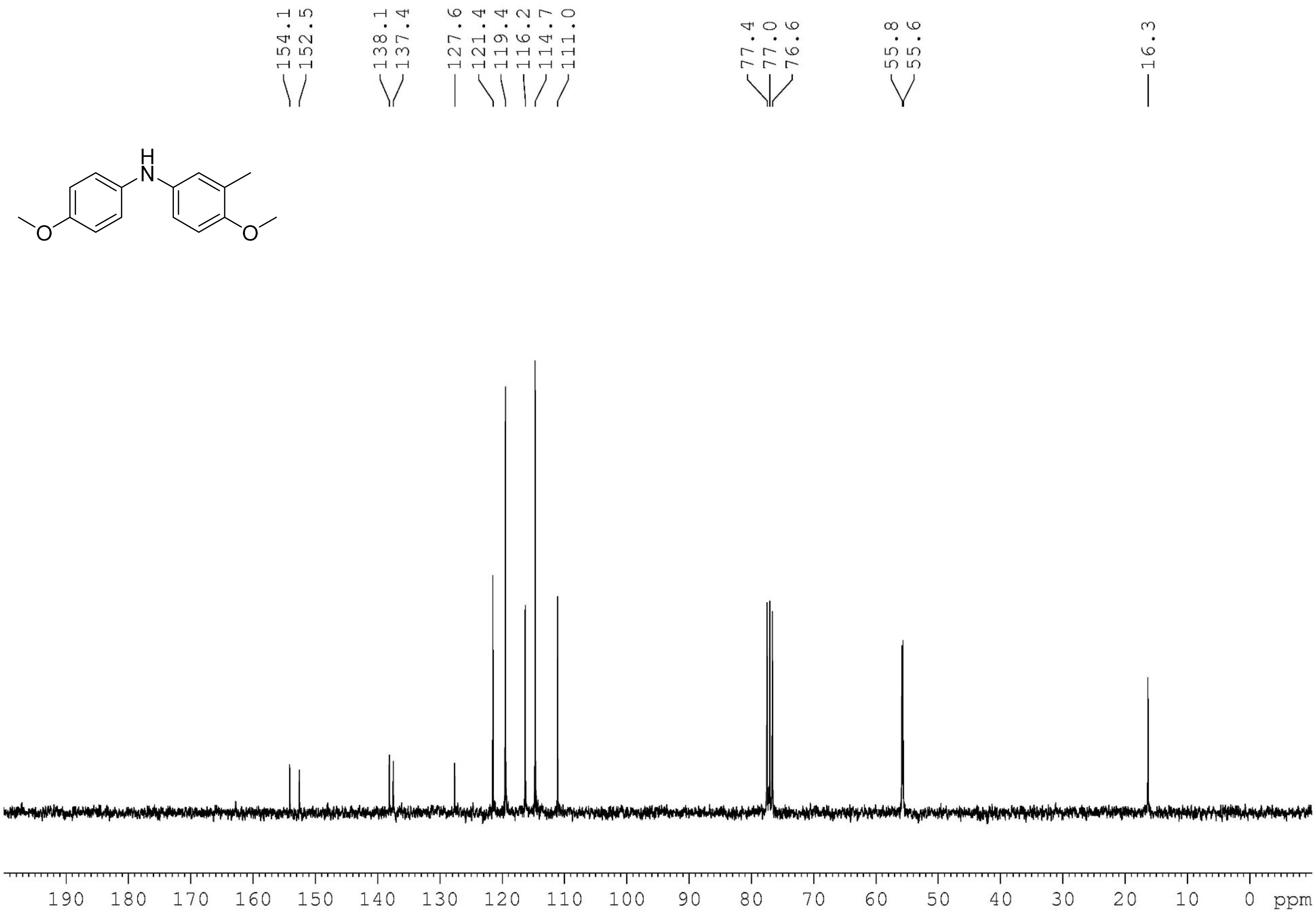

$^{13}C\{^{1}H\}$ NMR of compound **3ac** (75 MHz, $CDCl_3$)

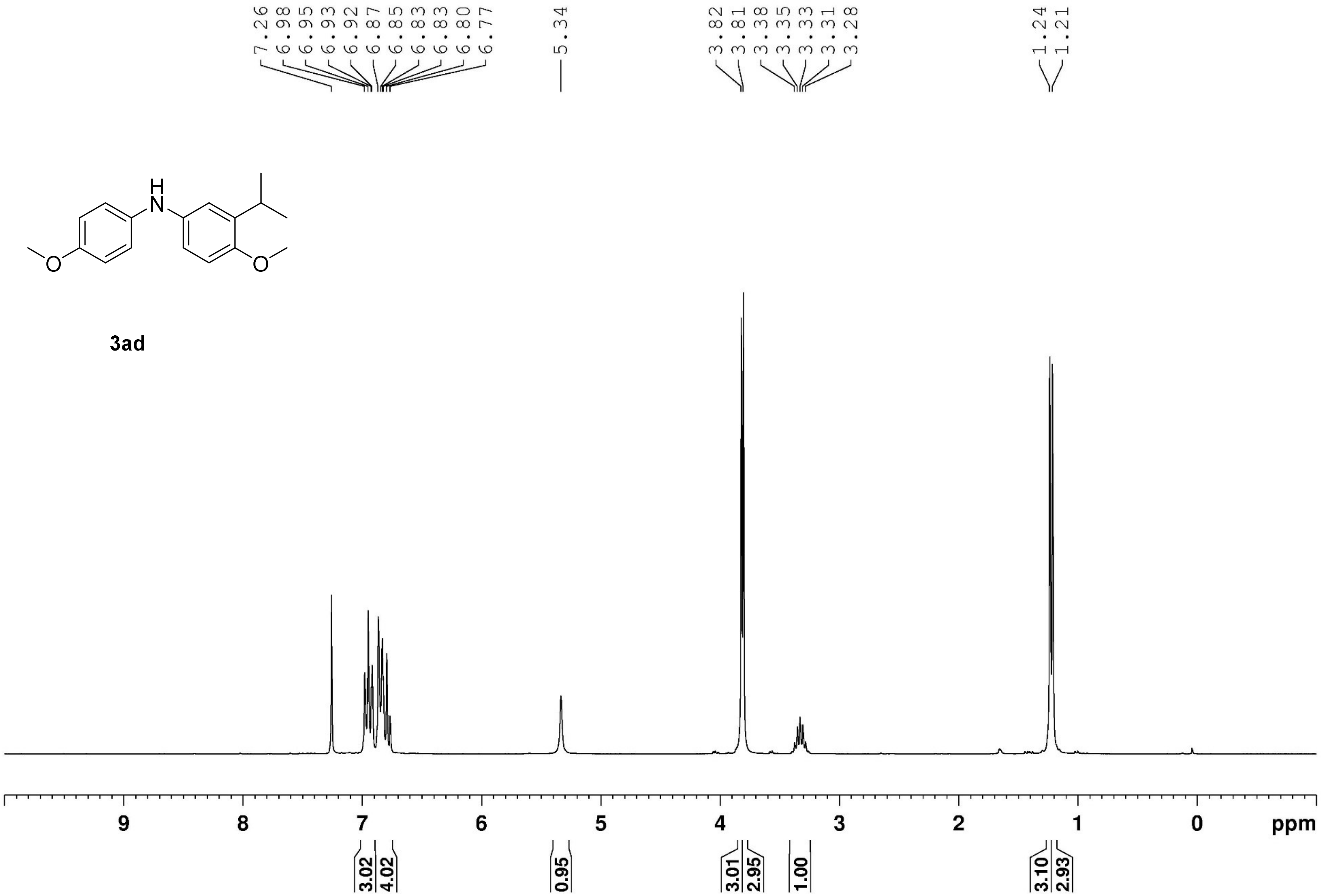

^{1}H NMR of compound **3ad** (300 MHz, $CDCl_3$)

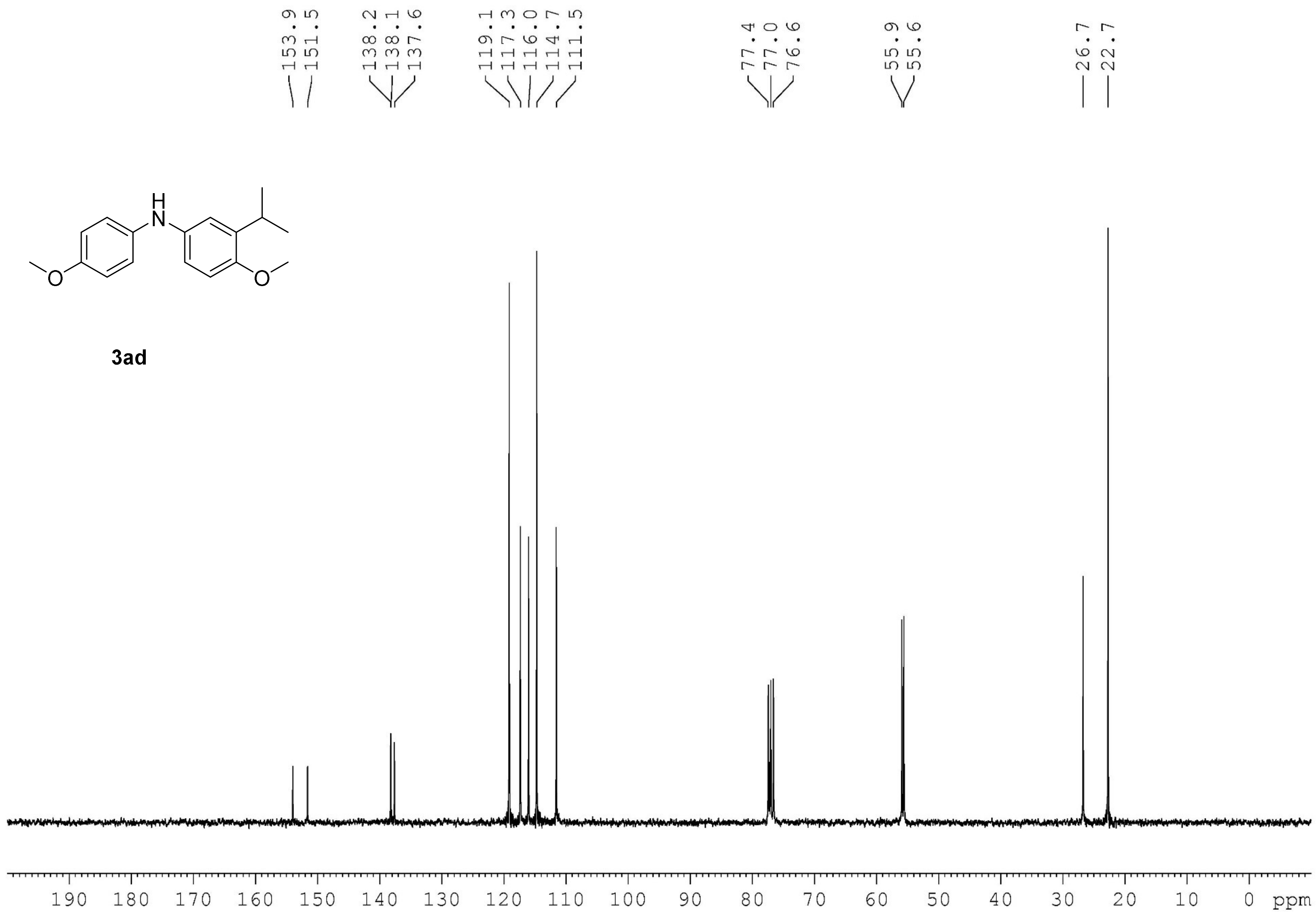

3ad

$^{13}C\{^1H\}$ NMR of compound **3ad** (75 MHz, $CDCl_3$)

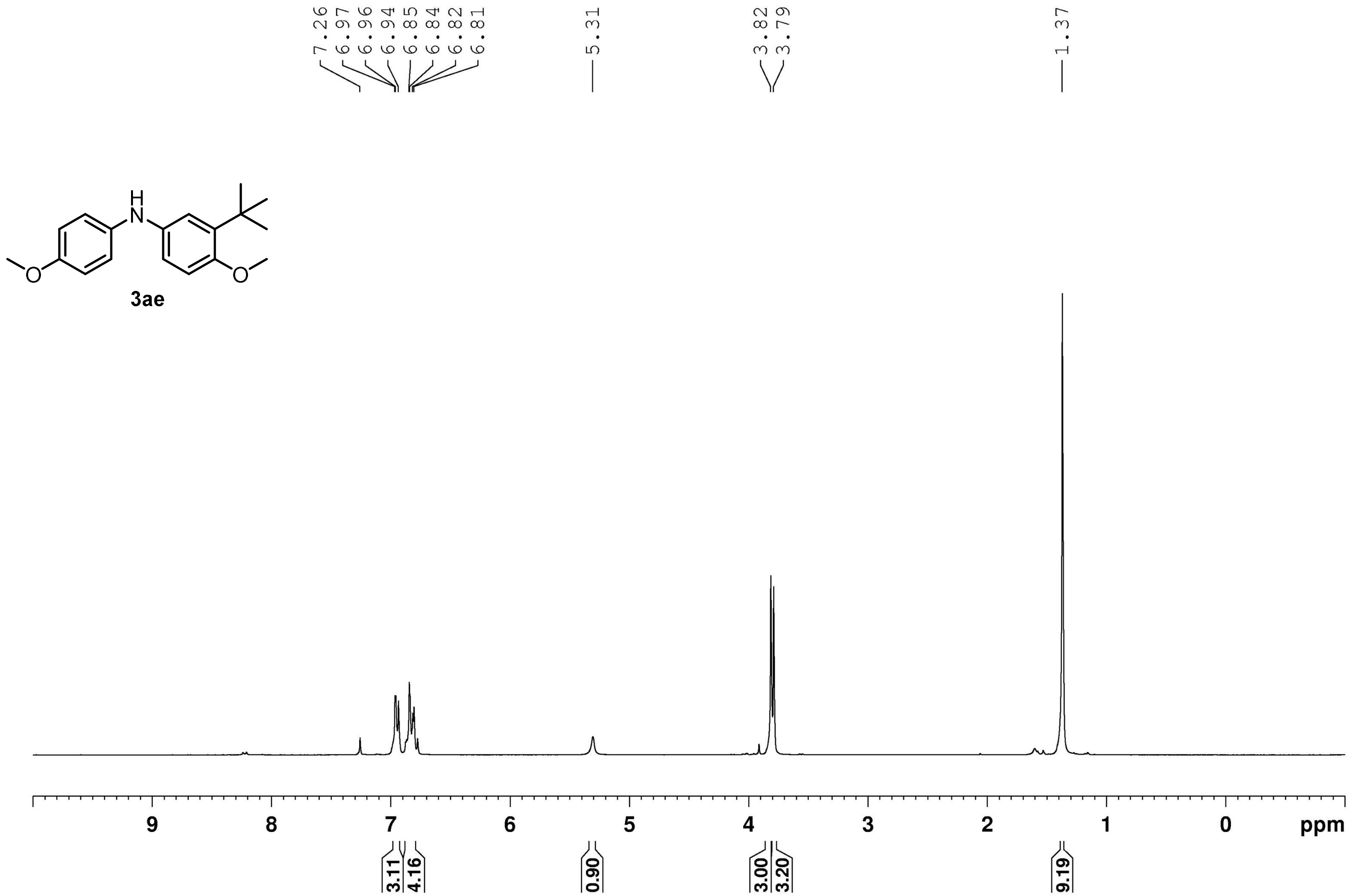

^{1}H NMR of compound **3ae** (300 MHz, $CDCl_3$)

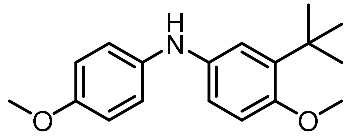

3ae

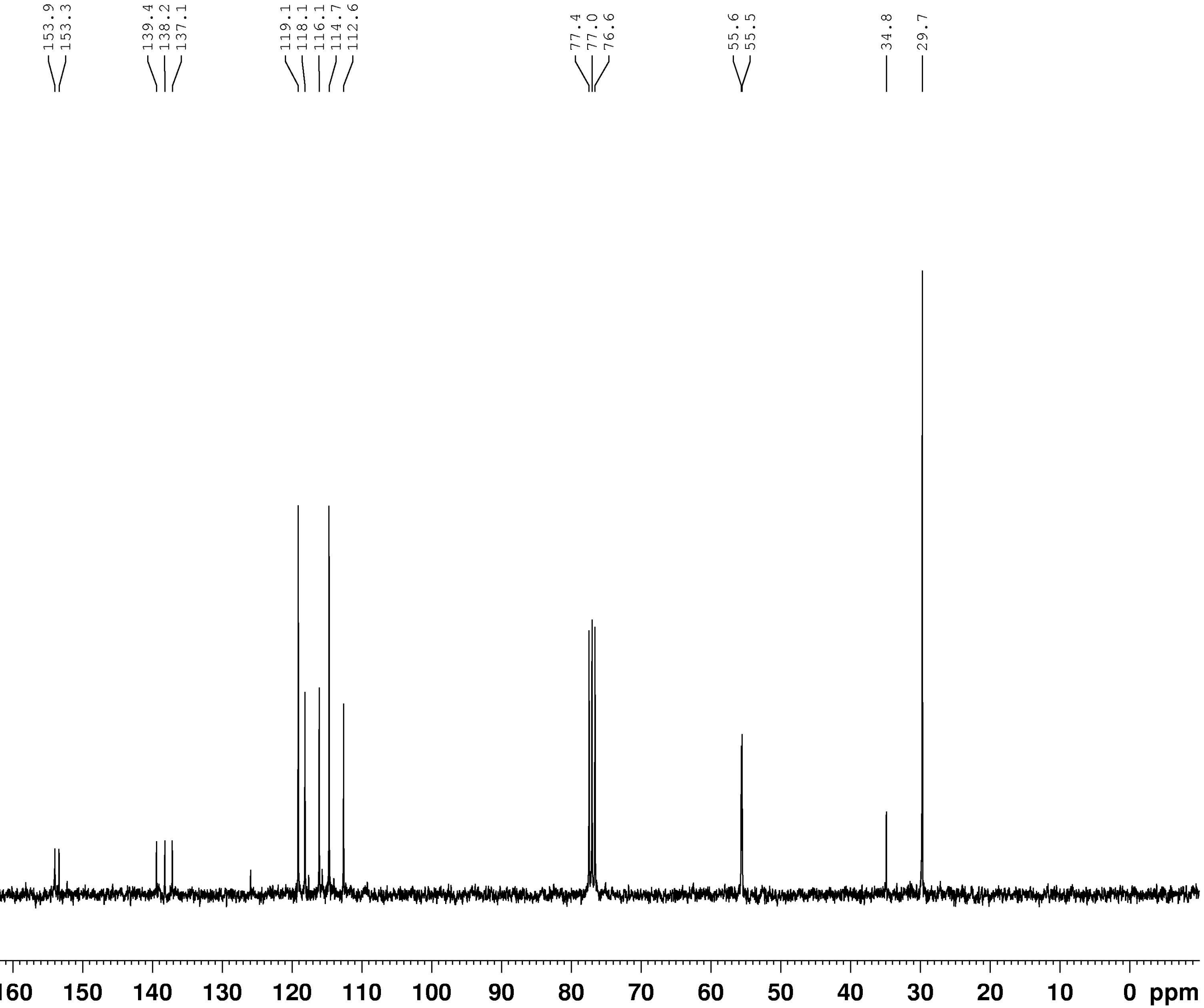

$^{13}C\{^{1}H\}$ NMR of compound **3ae** (75 MHz, $CDCl_3$)

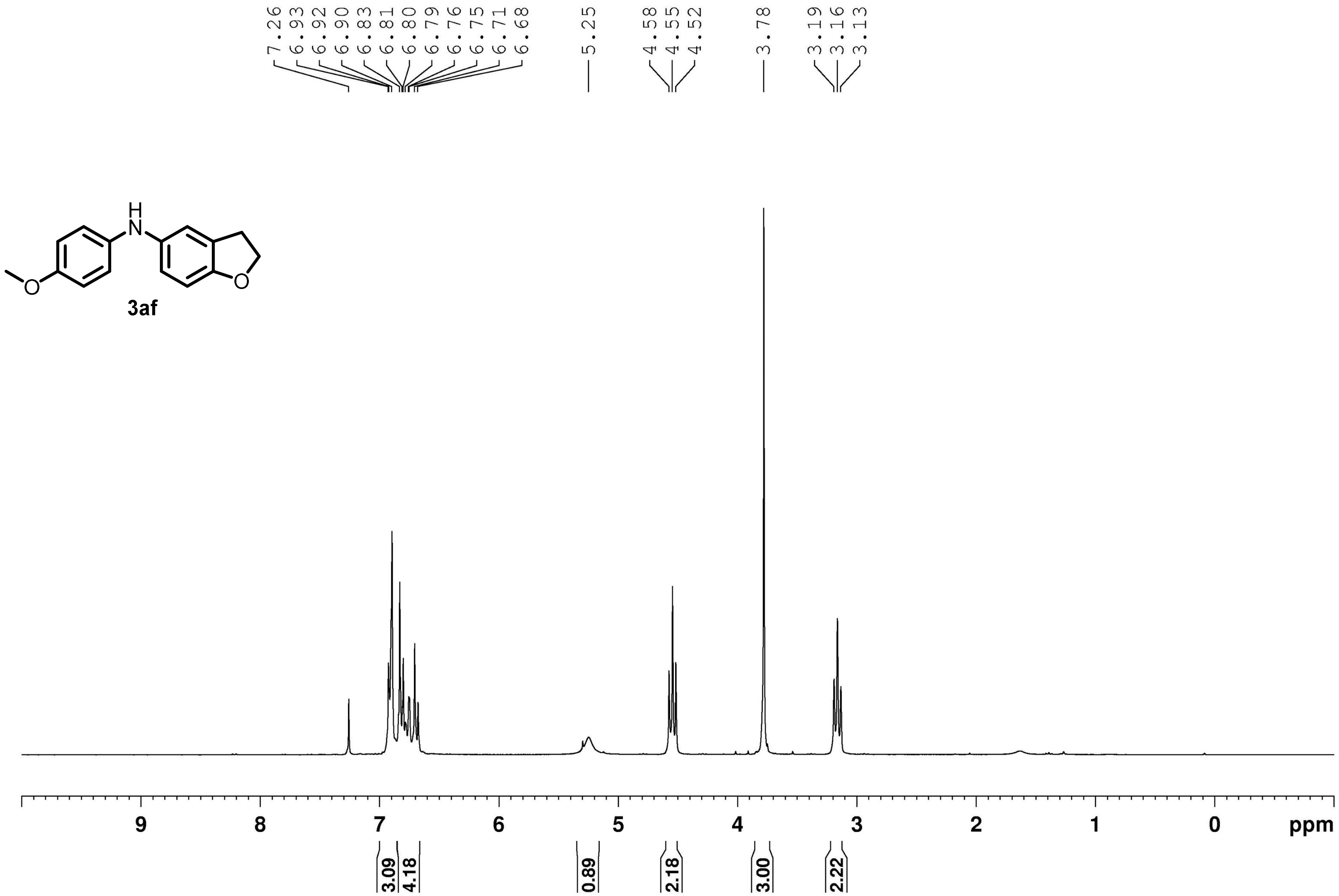

^{1}H NMR of compound **3af** (300 MHz, $CDCl_3$)

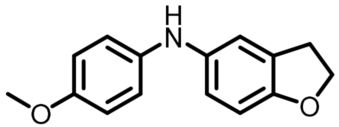

3af

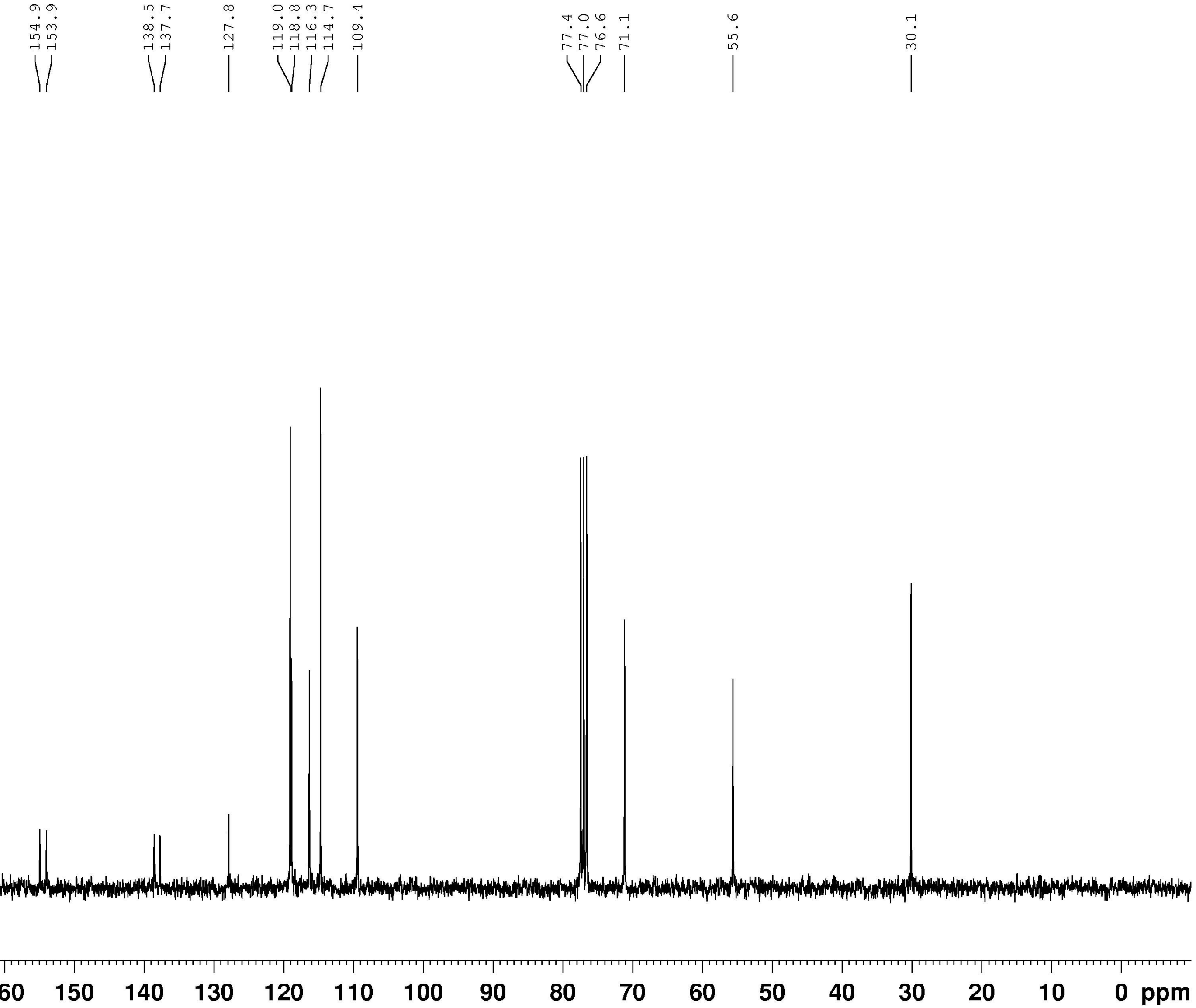

$^{13}C\{^{1}H\}$ NMR of compound **3af** (75 MHz, $CDCl_3$)

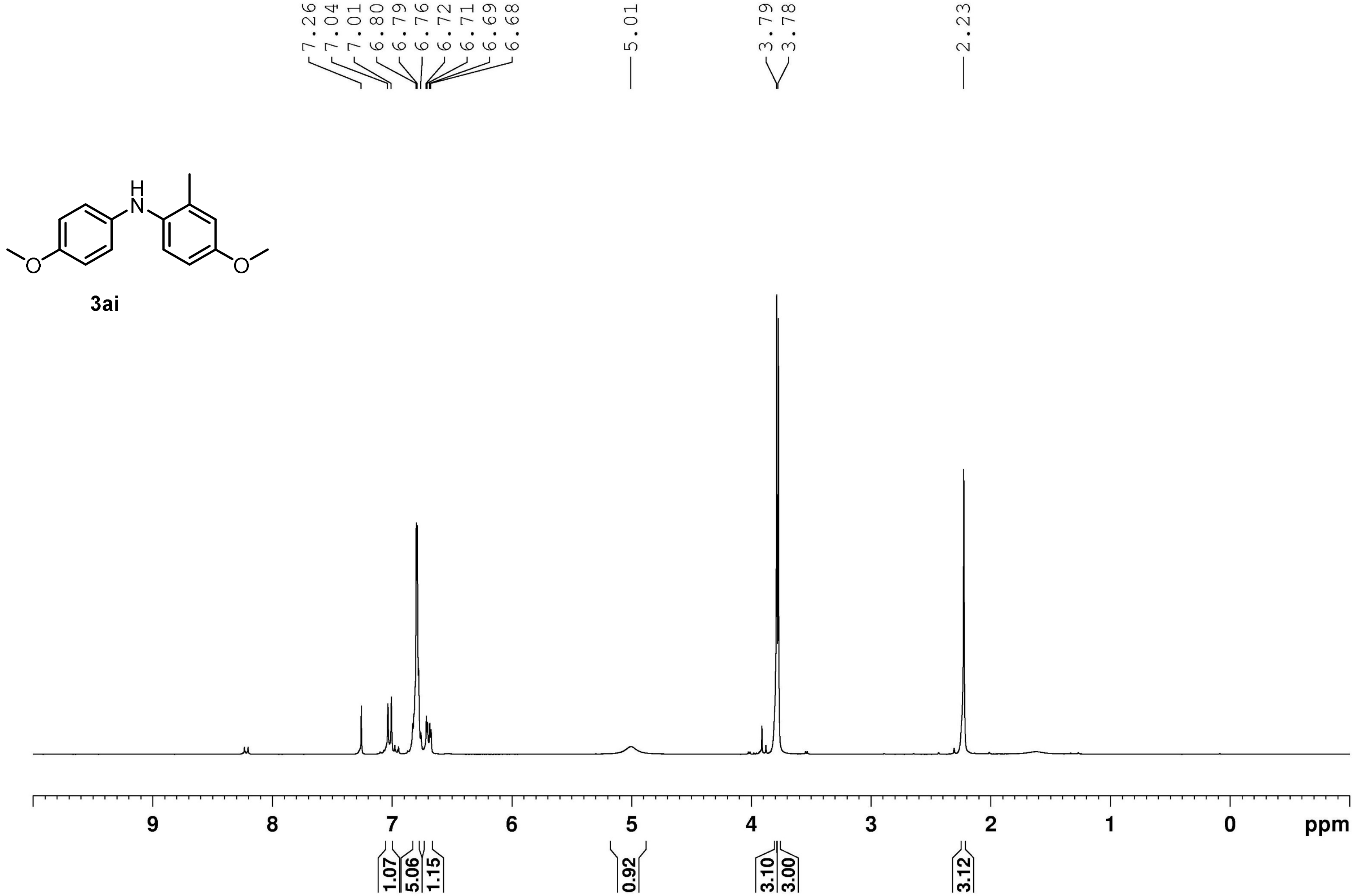

3ai

^{1}H NMR of compound **3ai** (300 MHz, $CDCl_3$)

3ai

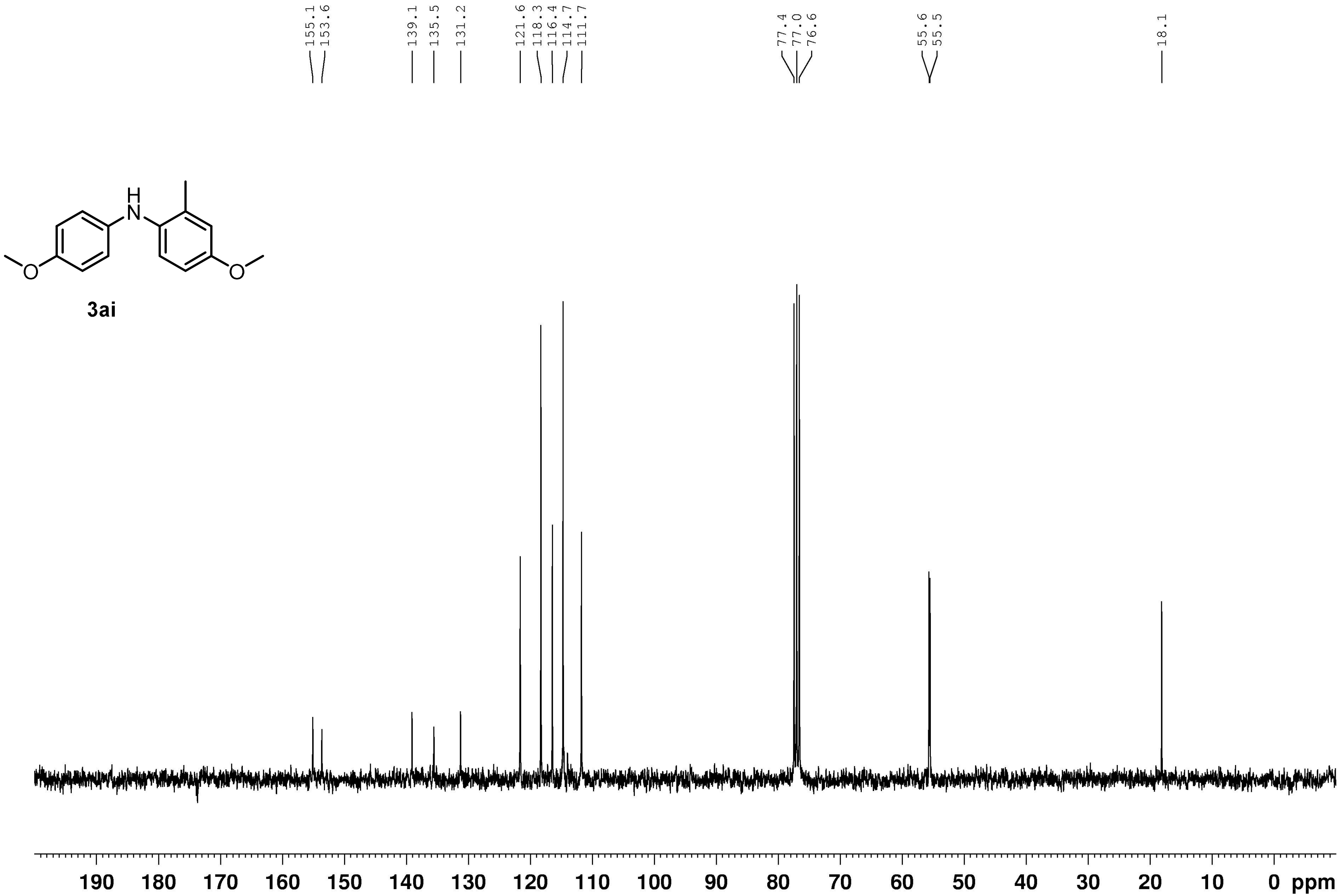

$^{13}C\{^{1}H\}$ NMR of compound **3ai** (75 MHz, $CDCl_3$)

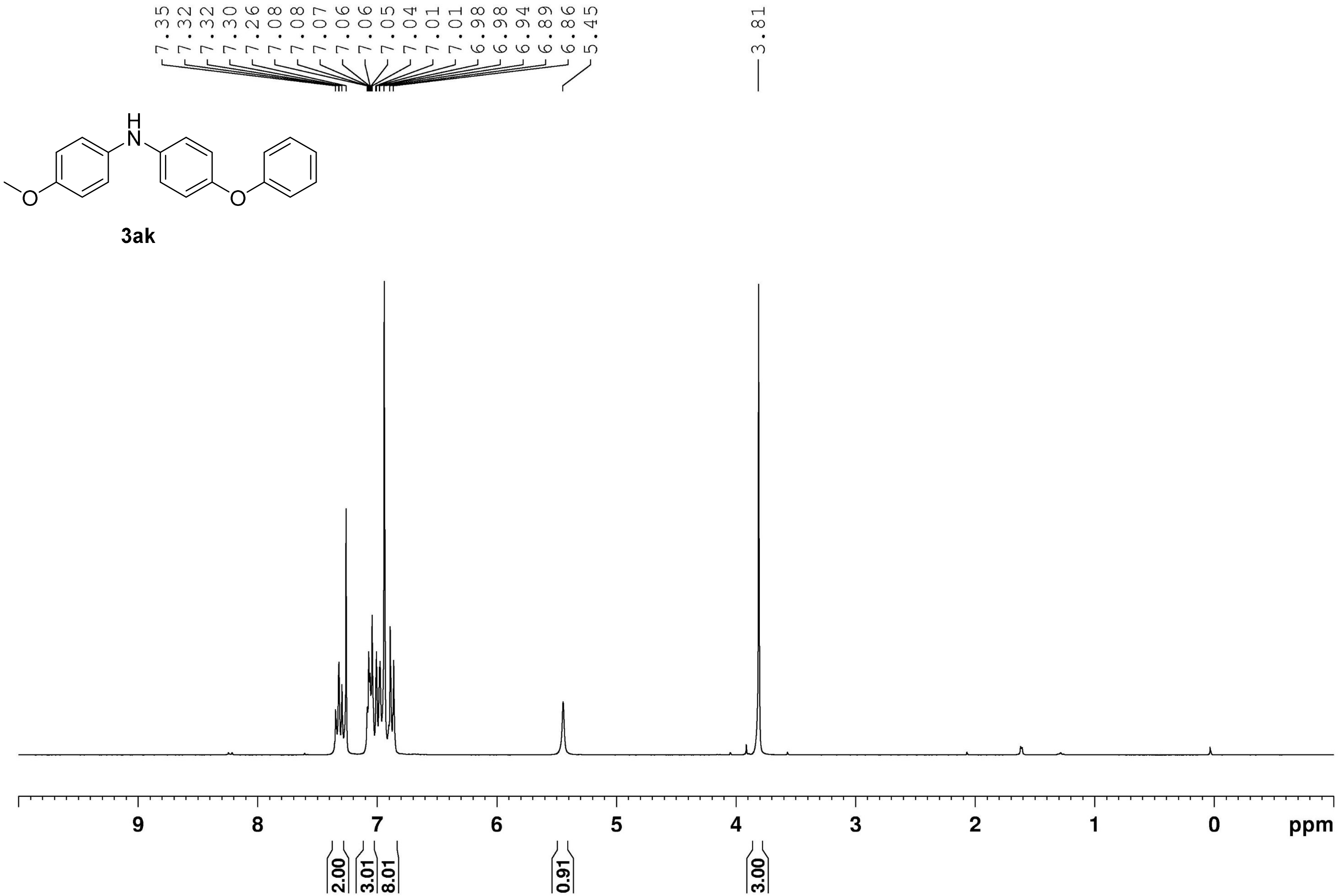

^{1}H NMR of compound **3ak** (300 MHz, $CDCl_3$)

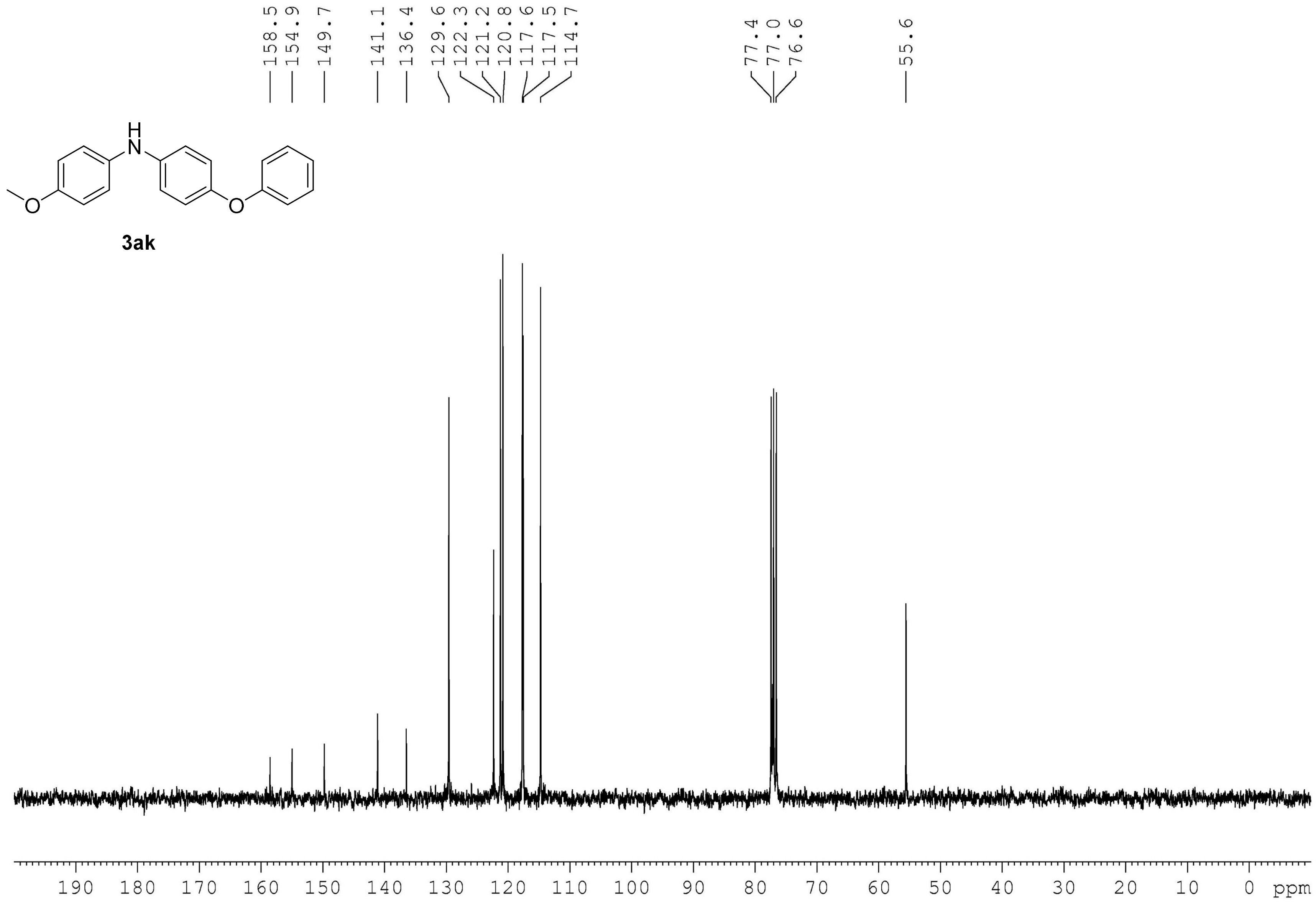

$^{13}C\{^{1}H\}$ NMR of compound **3ak** (75 MHz, $CDCl_3$)

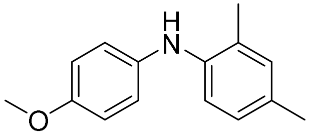

3an

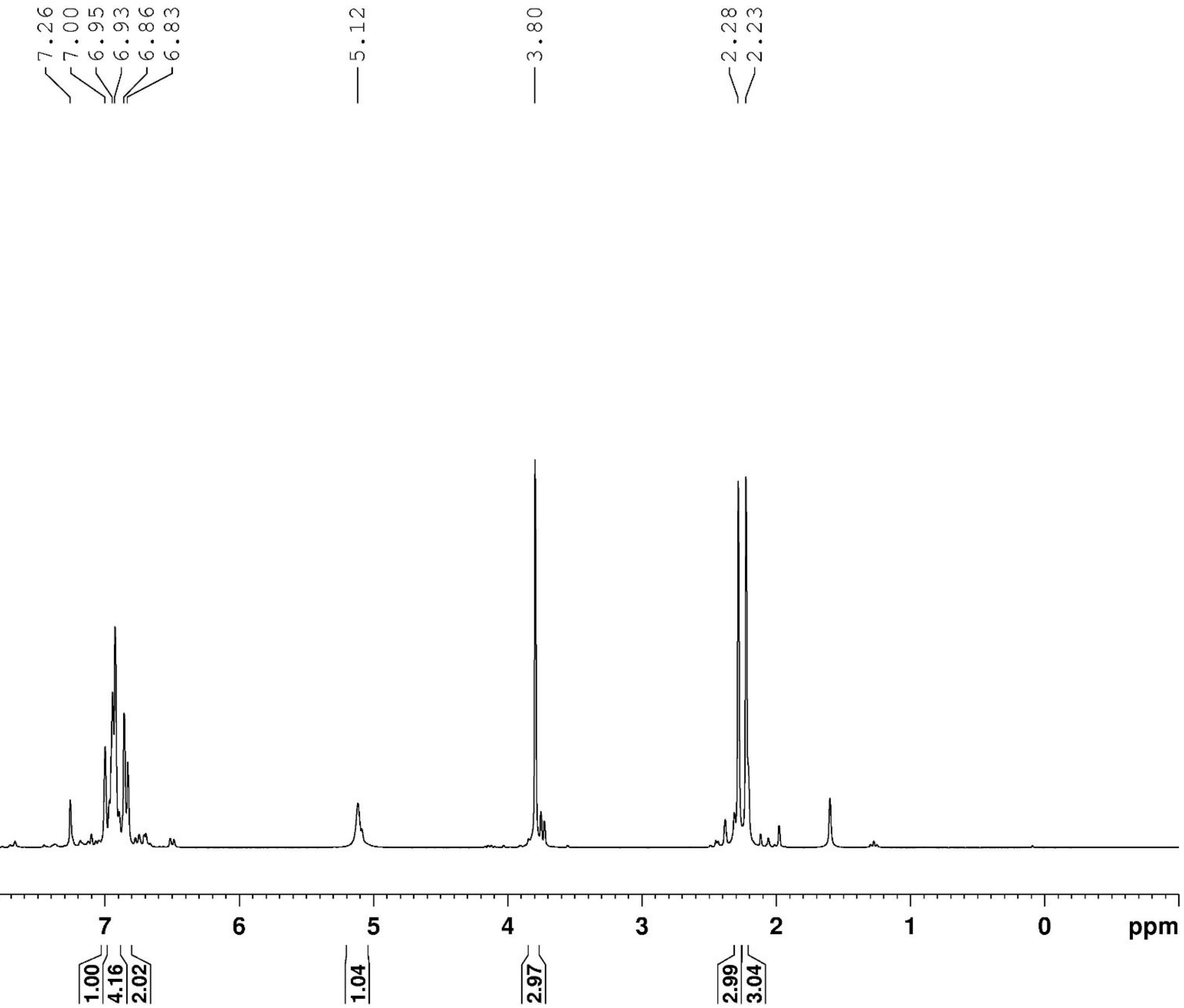

^{1}H NMR of compound **3an** (300 MHz, $CDCl_3$)

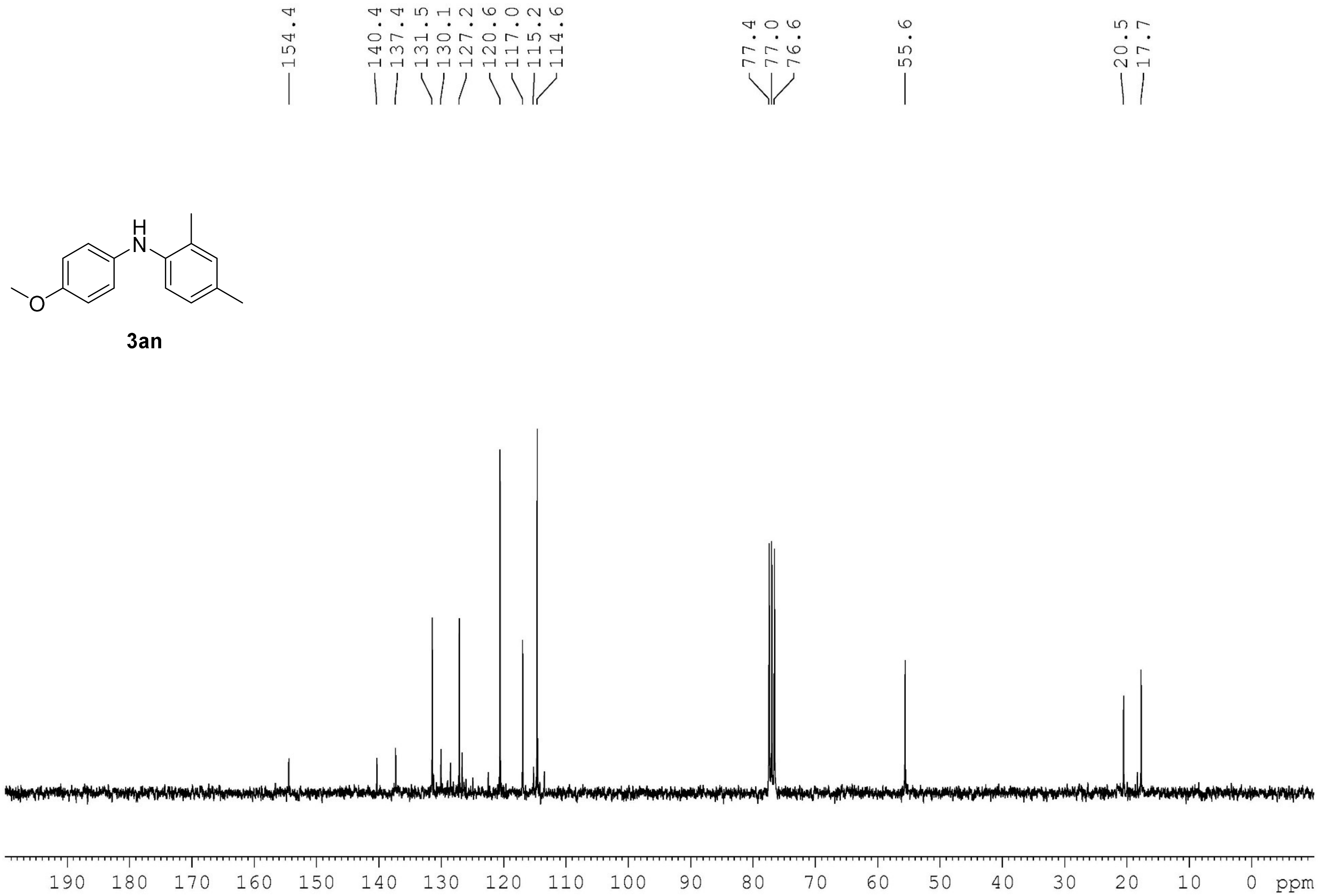

3an

$^{13}C\{^1H\}$ NMR of compound **3an** (75 MHz, $CDCl_3$)

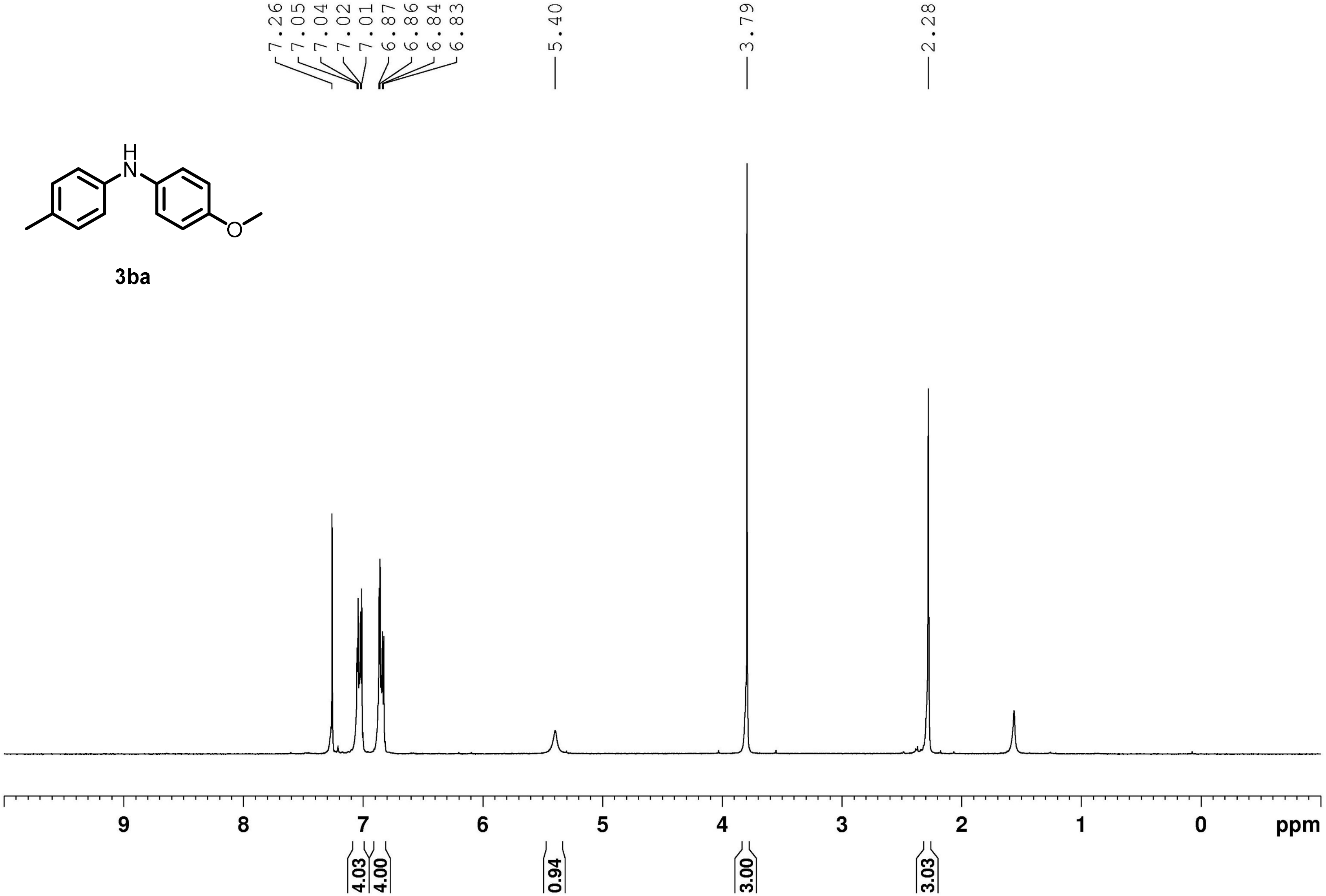

^{1}H NMR of compound **3ba** (300 MHz, $CDCl_3$)

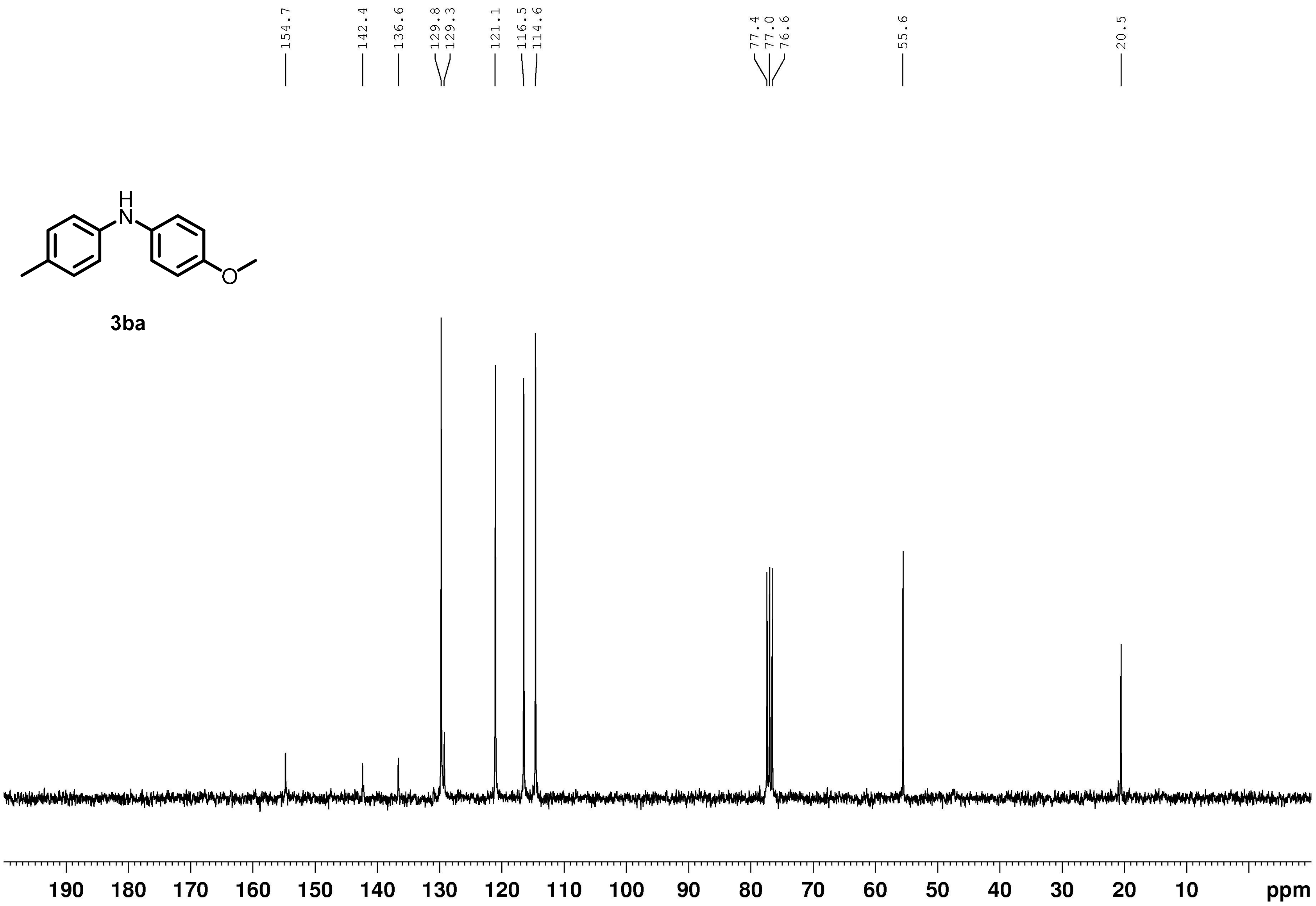

3ba

$^{13}C\{^{1}H\}$ NMR of compound **3ba** (75 MHz, $CDCl_3$)

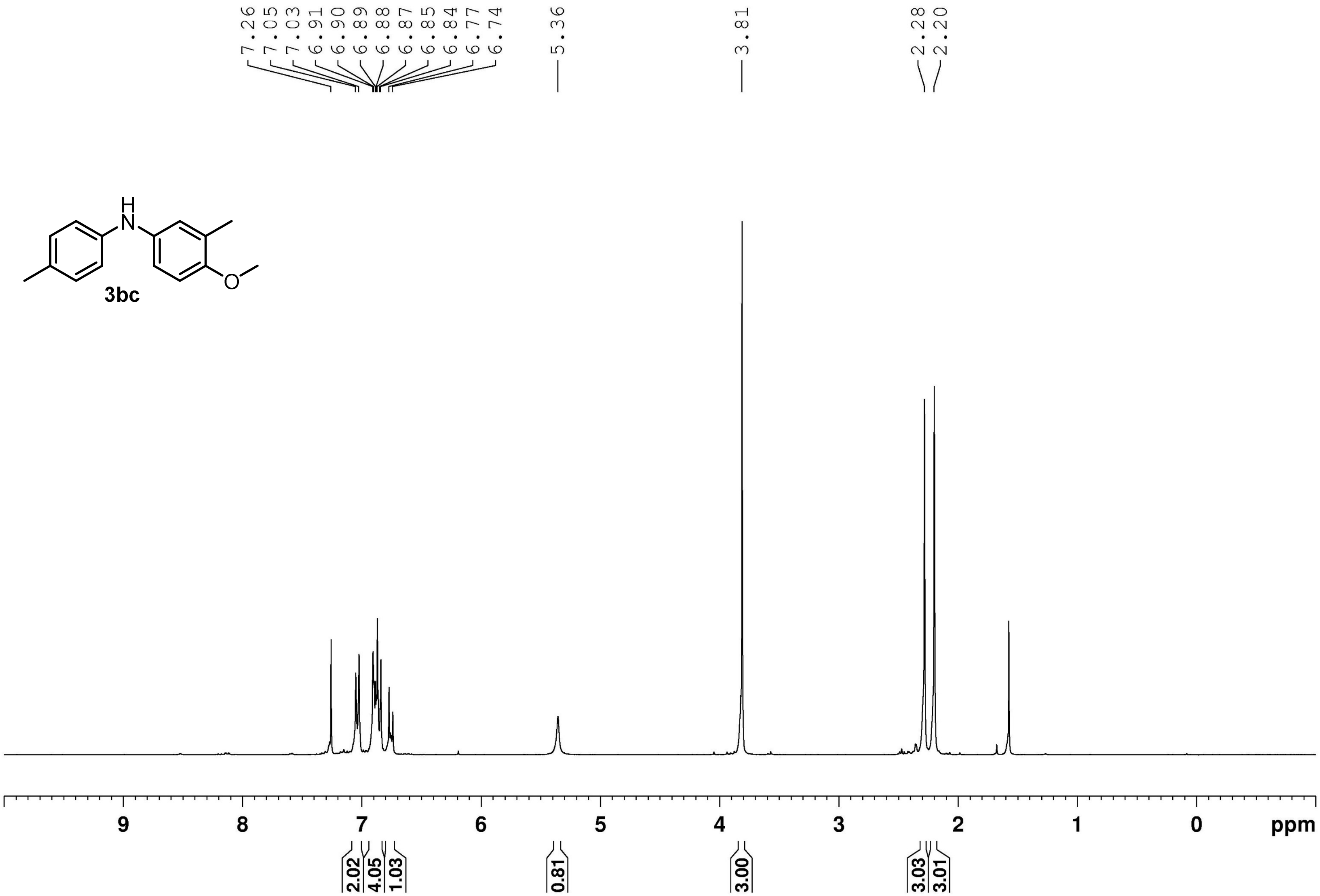

^{1}H NMR of compound **3bc** (300 MHz, $CDCl_3$)

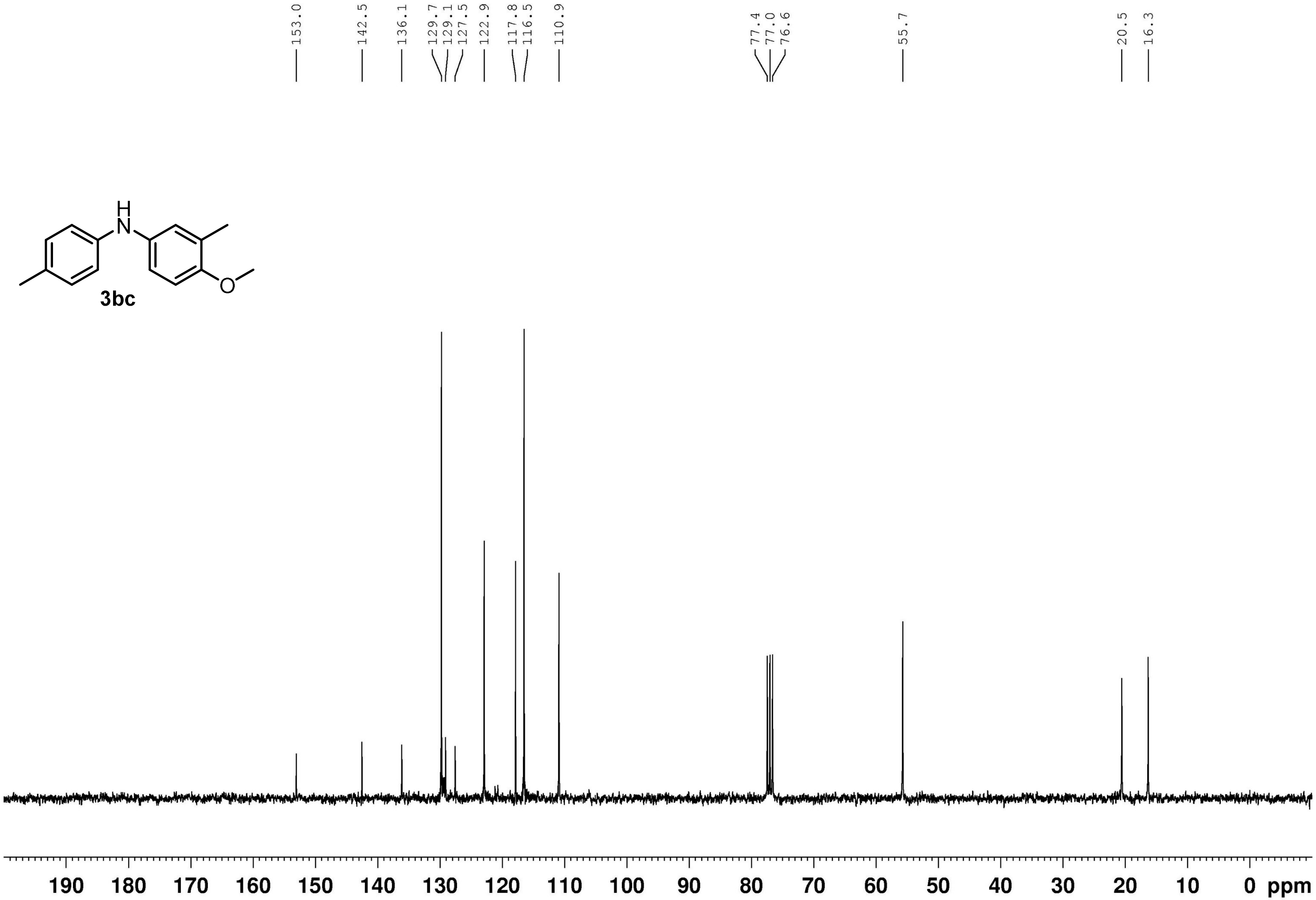

$^{13}C\{^1H\}$ NMR of compound **3bc** (75 MHz, $CDCl_3$)

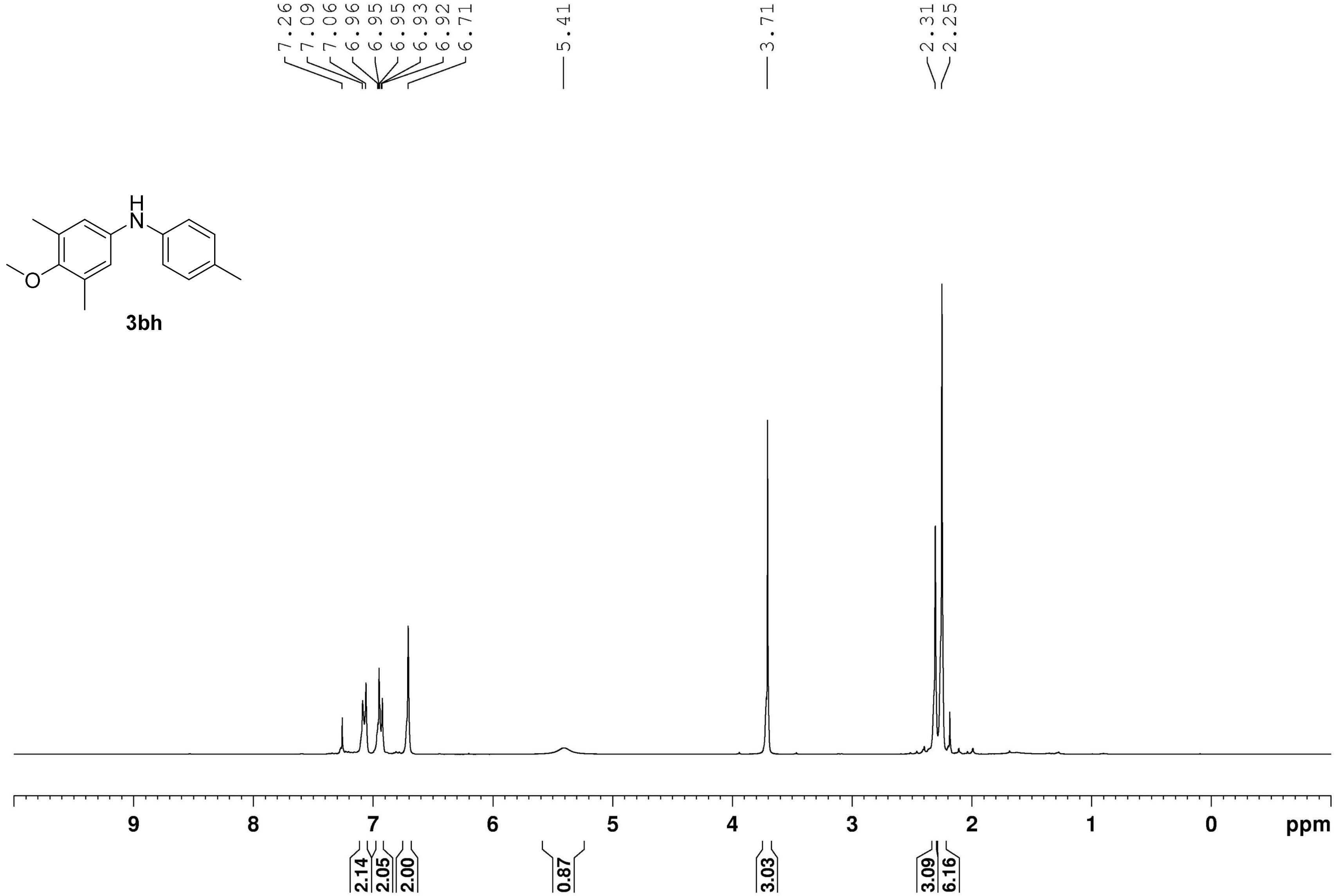

3bh

^{1}H NMR of compound **3bh** (300 MHz, $CDCl_3$)

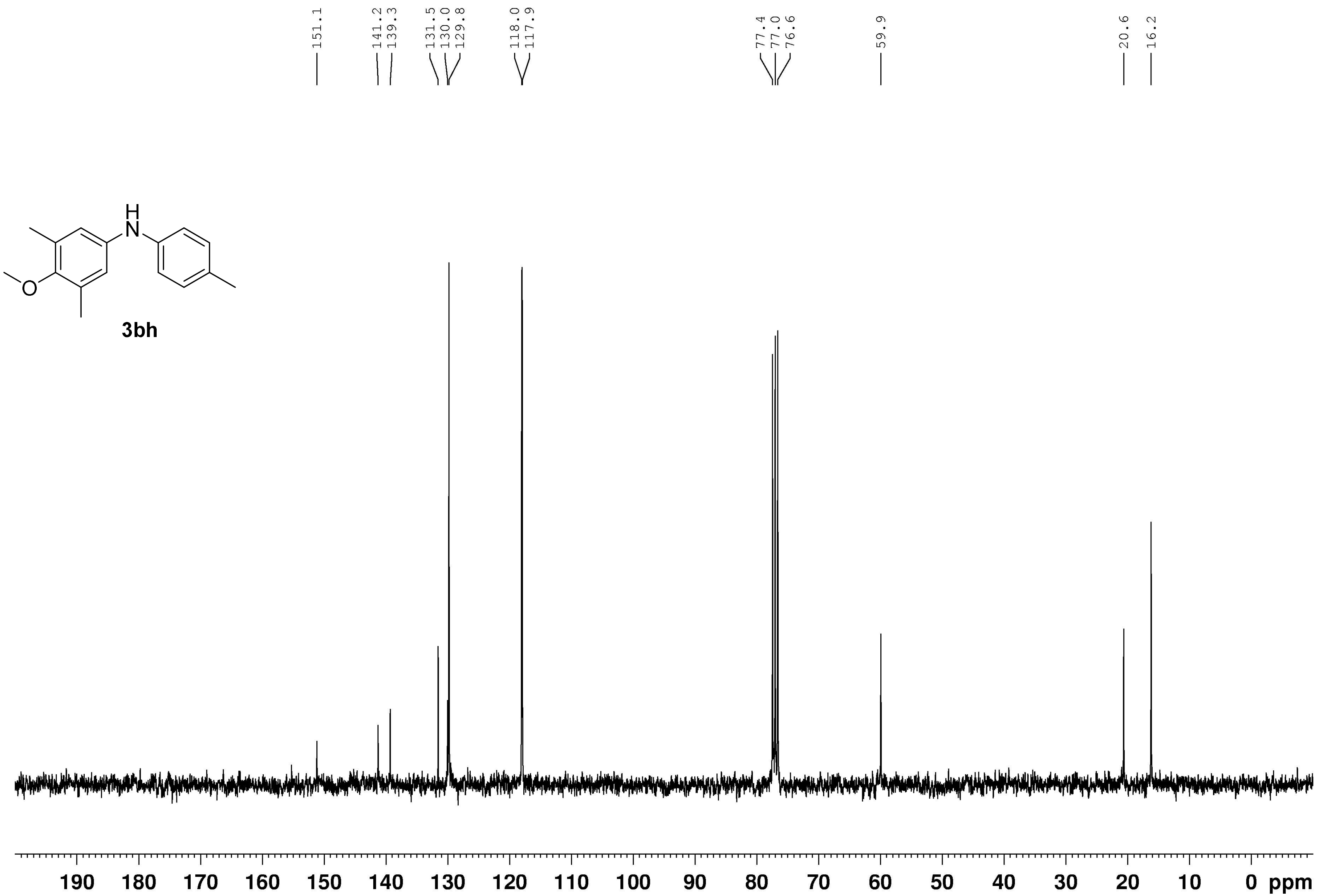

3bh

$^{13}C\{^{1}H\}$ NMR of compound **3bh** (75 MHz, $CDCl_3$)

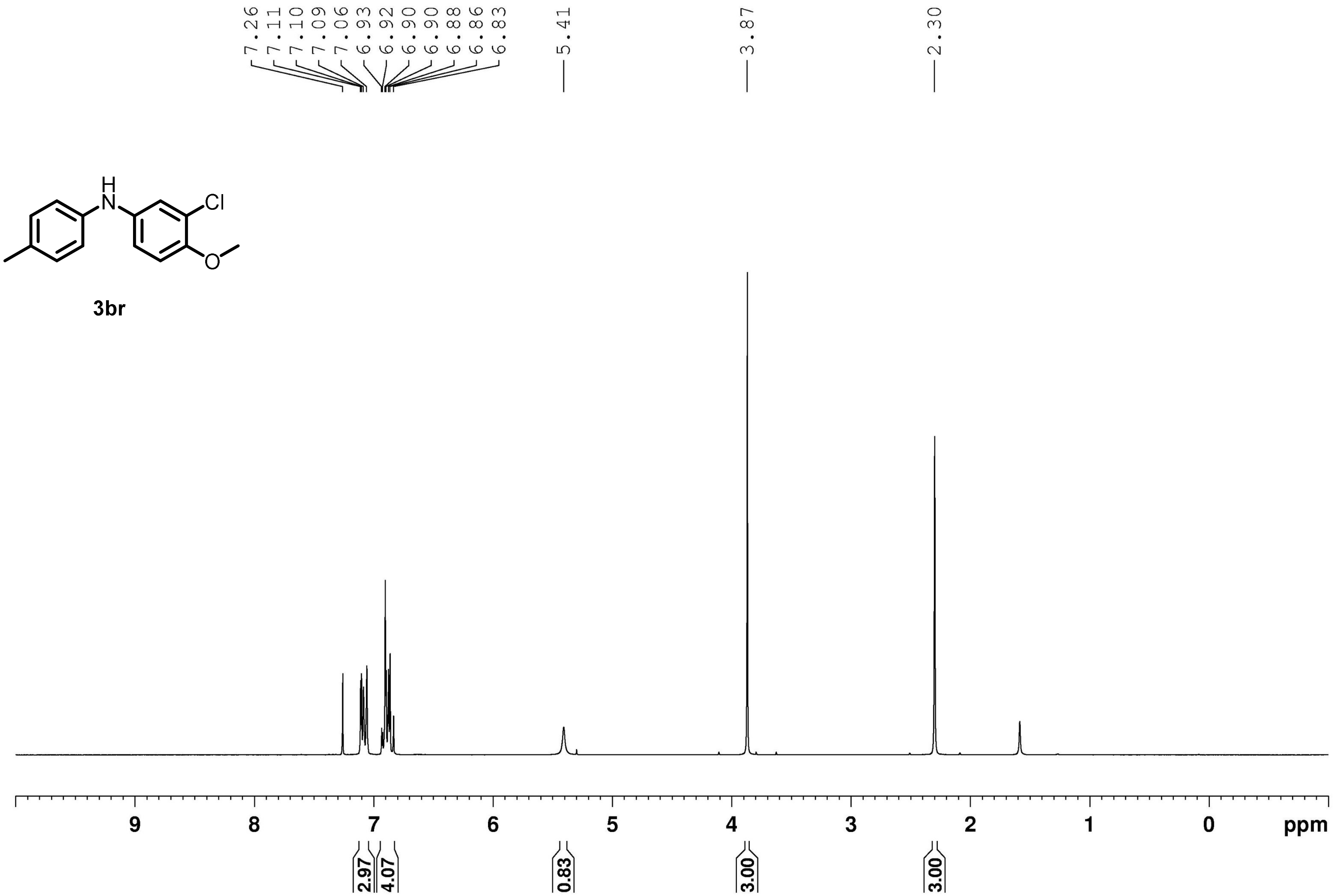

^{1}H NMR of compound **3br** (300 MHz, $CDCl_3$)

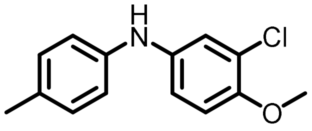

3br

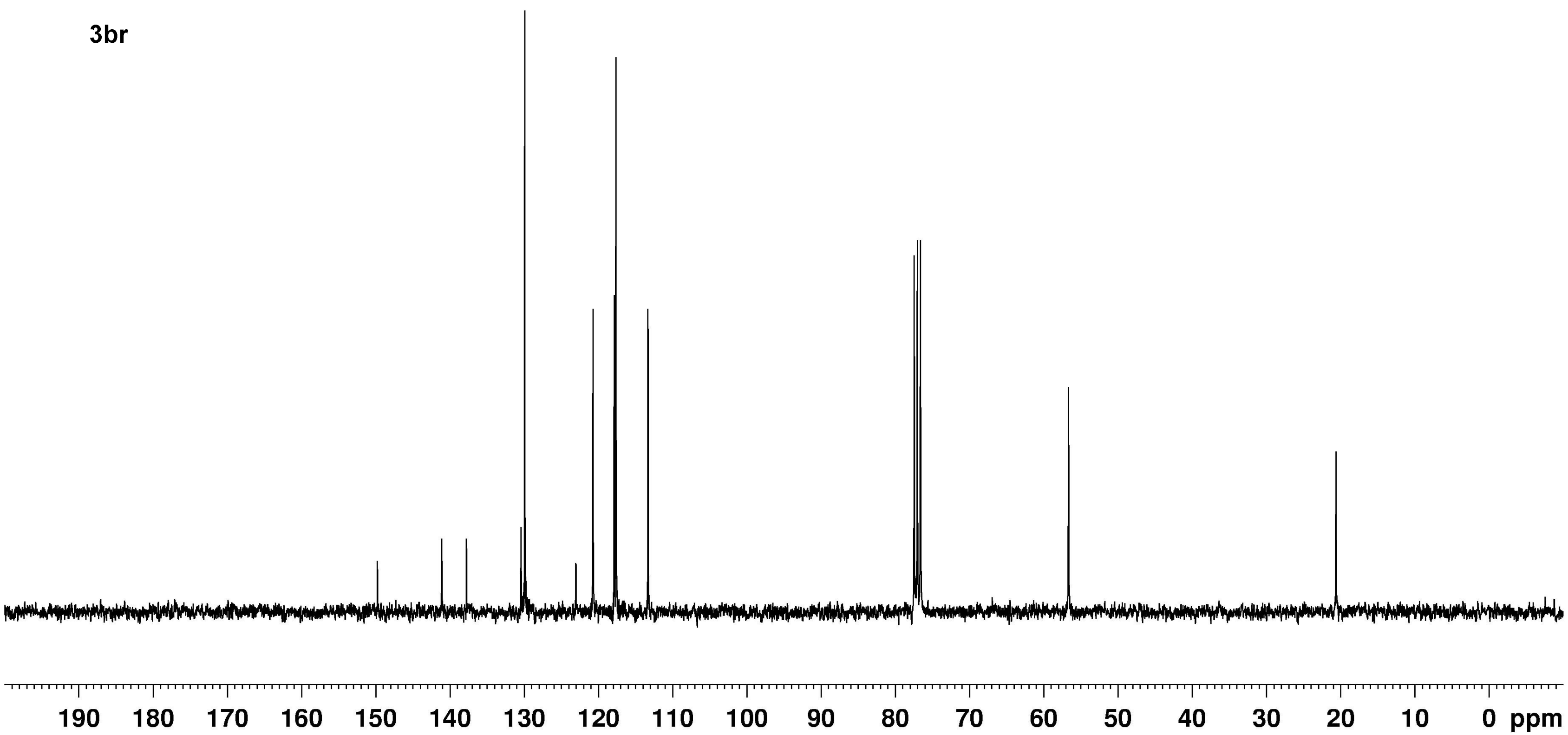

$^{13}C\{^{1}H\}$ NMR of compound **3br** (75 MHz, $CDCl_3$)

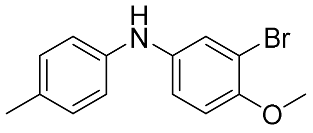

3bs

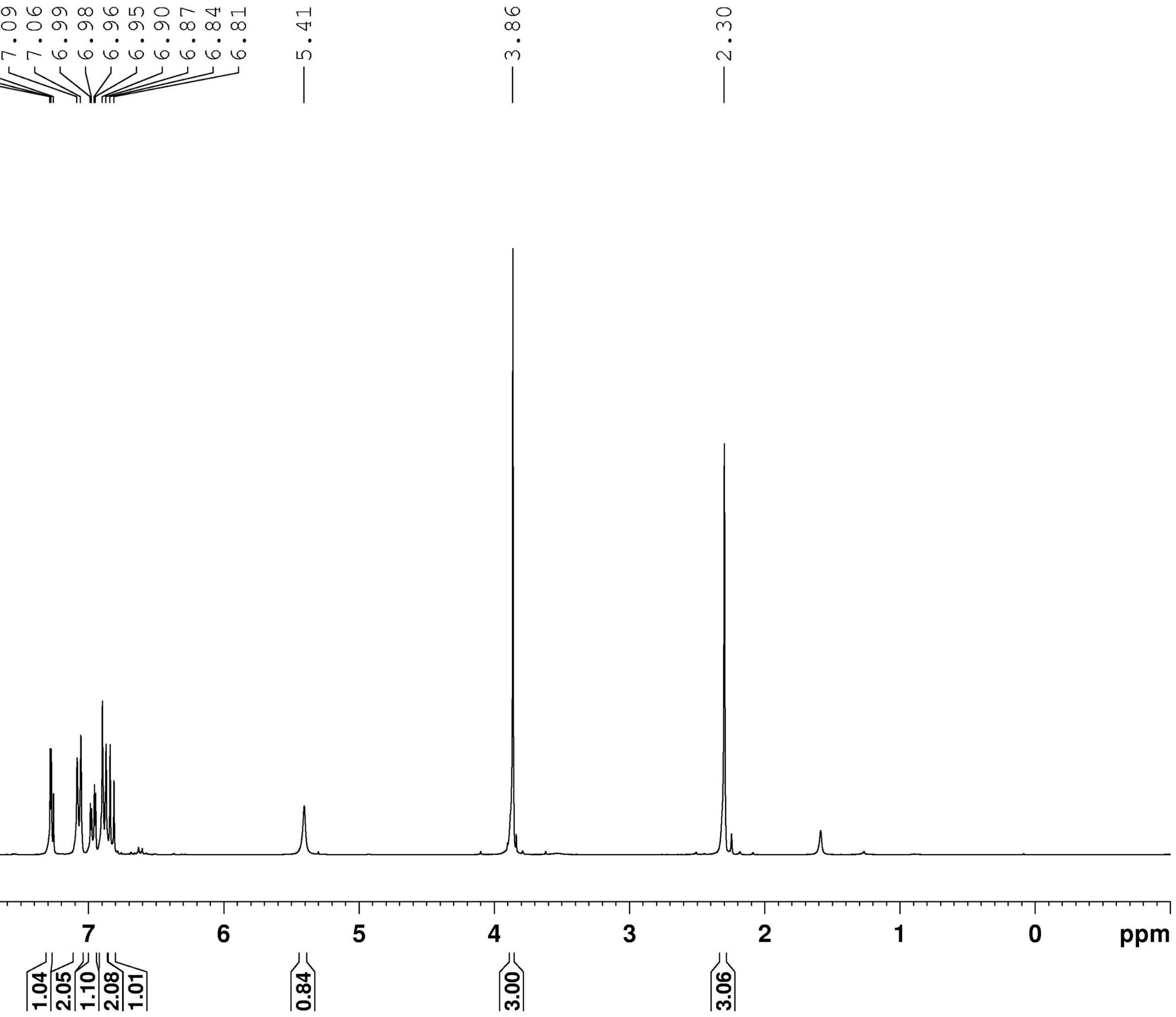

^{1}H NMR of compound **3bs** (300 MHz, $CDCl_3$)

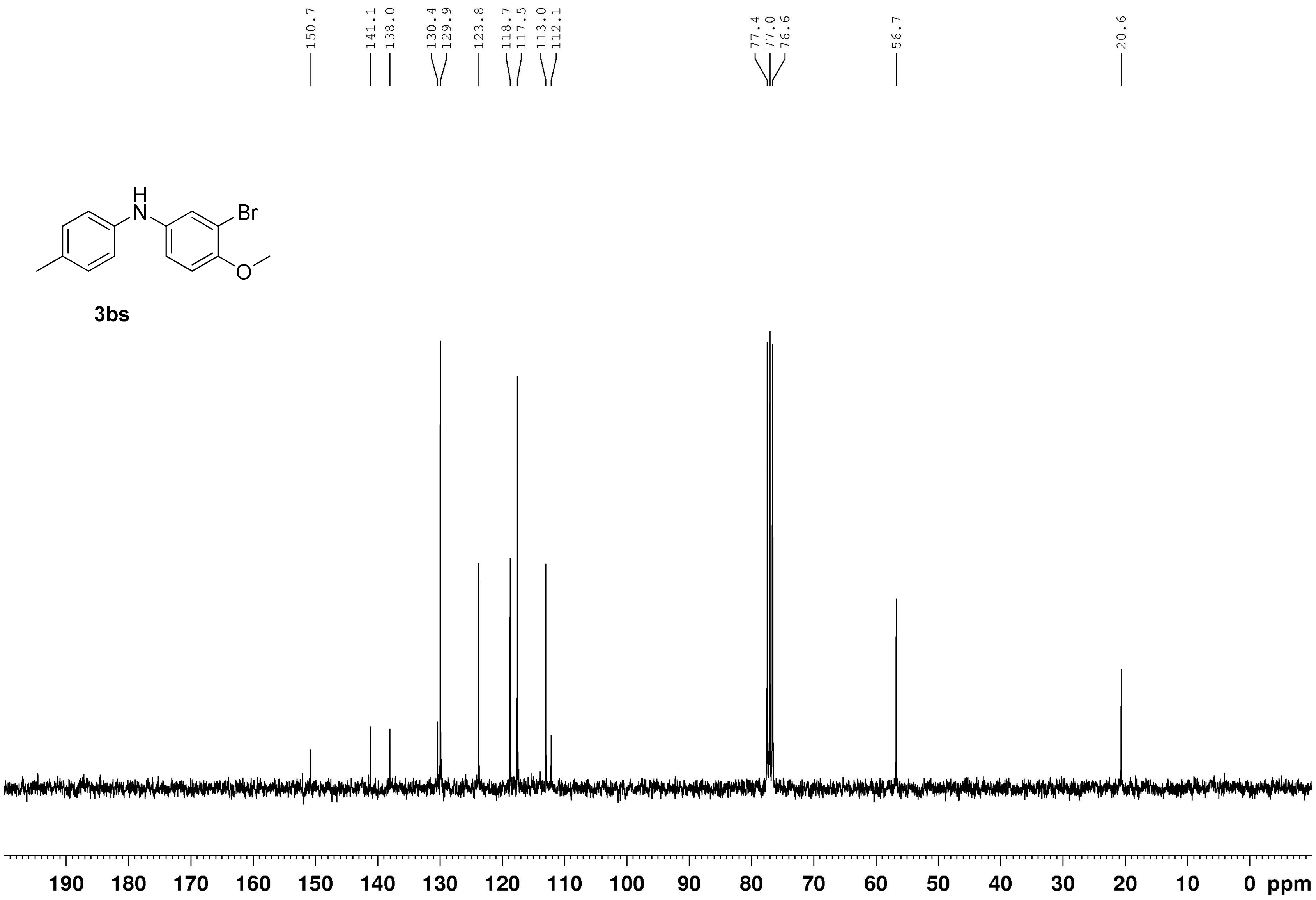

$^{13}C\{^{1}H\}$ NMR of compound **3bs** (75 MHz, $CDCl_3$)

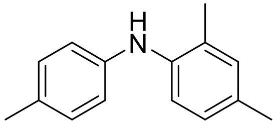

3bn

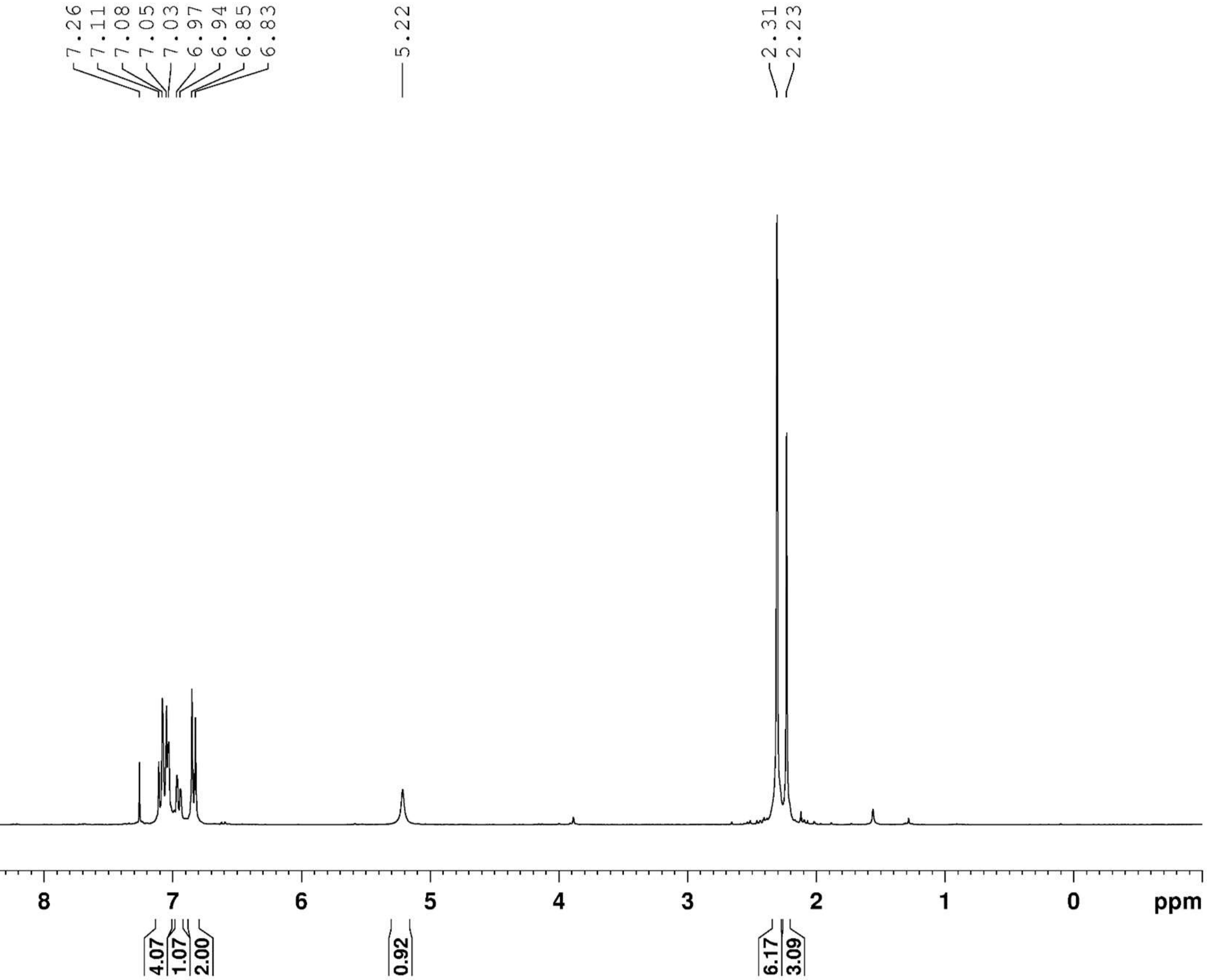

^{1}H NMR of compound **3bs** (300 MHz, $CDCl_3$)

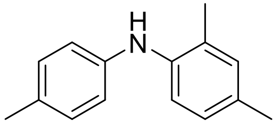

3bn

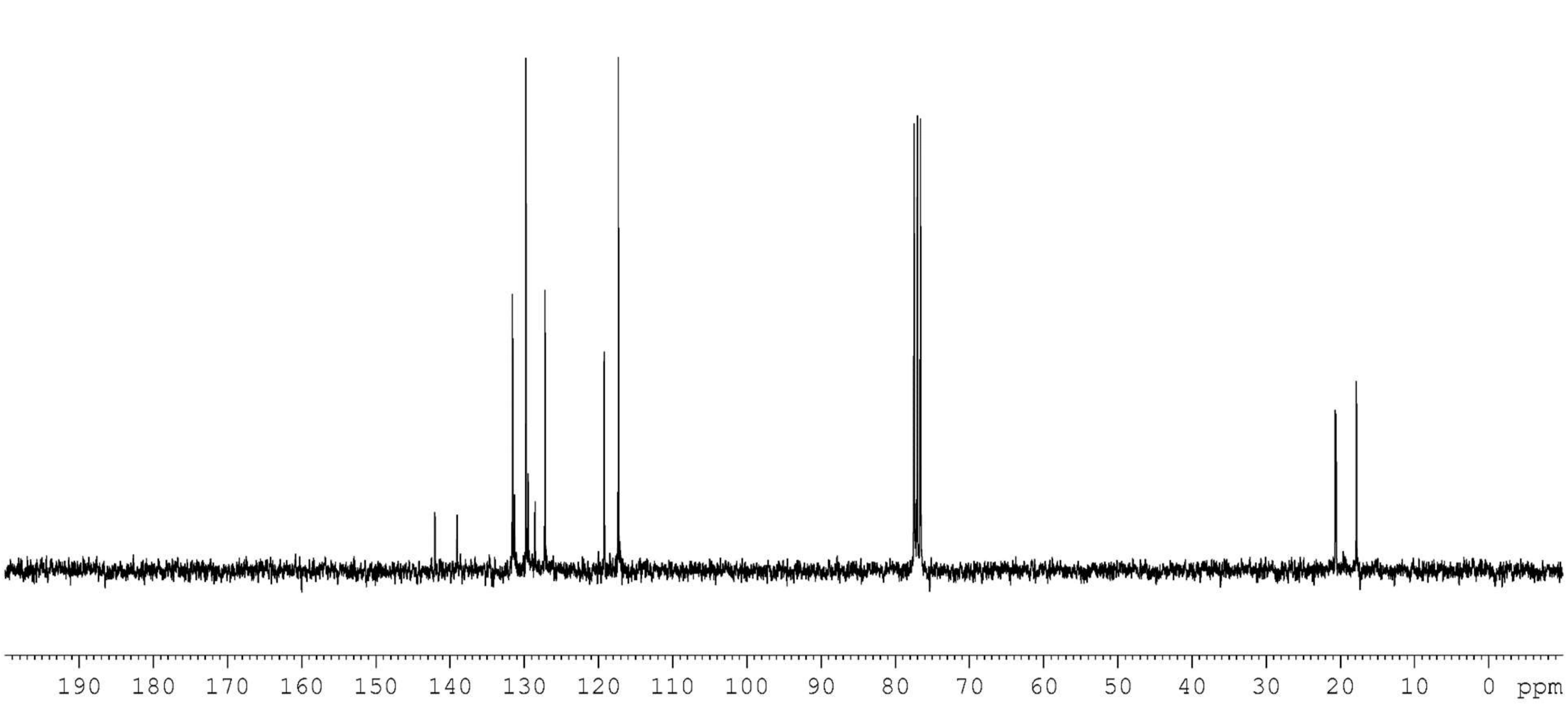

$^{13}C\{^{1}H\}$ NMR of compound **3bn** (75 MHz, $CDCl_3$)
